# Canine genome-wide association study identifies *DENND1B* as an obesity gene in dogs and humans

**DOI:** 10.1126/science.ads2145

**Published:** 2025-03-28

**Authors:** Natalie J. Wallis, Alyce McClellan, Alexander Mörseburg, Katherine A. Kentistou, Aqfan Jamaluddin, Georgina K.C. Dowsett, Ellen Schofield, Anna Morros-Nuevo, Sadia Saeed, Brian Y.H. Lam, Natasha T. Sumanasekera, Justine Chan, Sambhavi S. Kumar, Rey M Zhang, Jodie F. Wainwright, Marie Dittmann, Gabriella Lakatos, Kara Rainbow, David Withers, Rebecca Bounds, Marcella Ma, Alexander J. German, Jane Ladlow, David Sargan, Philippe Froguel, I. Sadaf Farooqi, Ken K. Ong, Giles S.H. Yeo, John A. Tadross, John R.B. Perry, Caroline M. Gorvin, Eleanor Raffan

**Affiliations:** 1 Department of Physiology, Development and Neuroscience, University of Cambridge; Cambridge, CB2 3EG, UK; 2 Metabolic Research Laboratories, Institute of Metabolic Science, University of Cambridge; Cambridge, CB2 0QQ, UK; 3 MRC Metabolic Diseases Unit, Institute of Metabolic Science, University of Cambridge; Cambridge, CB2 0QQ, UK; 4 MRC Epidemiology Unit, Institute of Metabolic Science, University of Cambridge; Cambridge, CB2 0QQ, UK; 5 Institute of Metabolism and Systems Research, University of Birmingham; Birmingham, B15 2TT, UK and Centre of Membrane Proteins and Receptors (COMPARE), Universities of Birmingham & Nottingham, UK; 6 Department of Veterinary Medicine, University of Cambridge; Cambridge, CB3 0ES, UK; 7 INSERM UMR 1283, CNRS UMR 8199, European Genomic Institute for Diabetes, Institut Pasteur de Lille, Lille, France; University of Lille, Lille University Hospital, Lille, France; and Department of Metabolism, Digestion and Reproduction, Imperial College London, London, London, SW7 2AZ, UK; 8 National Institute for Health and Care Research (NIHR) Cambridge Biomedical Research Centre, Hills Road, Cambridge, UK; 9 Institute of Life Course & Medical Sciences & School of Veterinary Science, University of Liverpool; Neston, CH64 7TE, UK; 10 Department of Histopathology and Cambridge Genomics Laboratory, Cambridge University Hospitals NHS Foundation Trust, Cambridge, CB2 0QQ, UK

## Abstract

Obesity is a heritable disease, but its genetic basis is incompletely understood. Canine population history facilitates trait mapping. We performed a canine genome-wide association study for body condition score, a measure of obesity, in 241 Labrador retrievers. Using a cross-species approach, we showed canine obesity genes are also associated with rare and common forms of obesity in humans. The lead canine association was within the gene DENN domain containing 1B (*DENND1B*). Each copy of the alternate allele was associated with ~7% greater body fat. We demonstrate a role for this gene in regulating signaling and trafficking of melanocortin 4 receptor, a critical controller of energy homeostasis. Thus, canine genetics identified obesity genes and mechanisms relevant to both dogs and humans.

Obesity is a complex disease resulting from a multitude of biological and environmental factors and is a major threat to both human and animal health worldwide. Studies in human patients with severe, early onset obesity highlighted the critical role of hypothalamic leptin-melanocortin signaling in the central control of energy balance. This homeostatic pathway integrates peripheral signals of energy status, translating them into alterations in energy expenditure and eating behavior (1). Large-scale population genomic studies in humans have identified >1000 BMI-associated loci, but moving from genetic association to mechanistic insight has been challenging, in part because it is hard to know which small-effect and non-coding loci justify resource-intensive follow-up (2).

Dogs are a compelling model of human obesity because they develop obesity subject to similar environmental influences and, notably, offer the opportunity for genetic discovery due to their distinctive genetic structure. Ancestral dog populations were diverse and genetically heterogenous but narrow population bottlenecks at breed formation mean modern breeds are genetically homogeneous with a relatively long-range linkage disequilibrium (LD) structure which renders even complex trait mapping remarkably tractable (3–7). This population history also resulted in a high frequency of genetic disease in some dog breeds (8, 9), including obesity (10–12). Additionally, there is greater homology between the dog and human genomes than between those of human and mouse (4). Despite these compelling reasons to use dogs for scientific discovery relevant to both canine and human biology, dogs have been underused as a model organism to date.

About 40-60% of pet dogs are overweight or obese (13, 14), predisposing them to a range of health problems (15, 16). Dogs are exposed to similar environmental risk factors for obesity as humans; most have limited exercise and easy access to food (17). Owner management of diet and exercise is important in determining obesity outcomes, as are other risk factors such as sex, gonadectomy status and age (15). Inheritance of obesity in dogs is complex and its genetic basis is poorly understood.

Only one small genome wide association study (GWAS) for obesity has been performed in dogs and it found no significant associations (18). However, a few causative mutations have been identified in candidate gene studies (16). One is a large effect mutation in the pro-opiomelanocortin gene (*POMC* p.P187fs), which is found in a quarter of pet Labrador retrievers and is associated with increased weight, adiposity and hunger, and lower energy expenditure (19, 20). Those canine studies corroborated the role of different *POMC* derived neuropeptides for activation of melanocortin 4 receptor in the hypothalamus, well recognized as a critical nexus of energy homeostasis in humans and other species (21–23). We hypothesized that other large effect genetic variants would influence obesity in the breed.

Labrador retrievers are particularly obesity-prone and tend to be highly food motivated (10, 11, 24). We studied a population of British Labrador retrievers and performed a GWAS which revealed multiple obesity-associated loci. We developed polygenic risk scores which explain previously observed obesity variation in the breed and quantify gene-environment interaction. Comparative genomics identified that canine obesity genes were also associated with human obesity. The gene most strongly associated in dogs was *DENND1B* which we studied in vitro to reveal it has a role in regulating MC4R signaling.

## Results

### Phenotypic characteristics and genetic data

We studied pet and working Labrador retrievers. Only adult dogs (age 1-10 years, mean 6 years) were included, free of known or suspected systemic illness and not being treated with medications likely to affect obesity status. Body fat mass was assessed using a well validated measure of adiposity, Body Condition Score (BCS) which uses a combination of haptic and visual cues to assign dogs to BCS categories 1-9 according to standardized descriptors (Fig. S1). On this scale BCS 4-5 are considered to represent optimal body fat mass and each point increase equates to ~8% increase in body fat mass with BCS 8-9 generally considered obese (25–27). To measure food motivation and owner control of diet and exercise, we used the validated, owner-reported Dog Obesity Risk Assessment questionnaire (DORA) which scores responses to a series of statements about dog behavior related to food in the home environment and owners’ management of diet and exercise (Table S1 and Supplementary Methods) (24).

For the discovery GWAS, we studied 241 pet dogs, all of which lived with their owners and some of which were also used as working dogs (for example, gun-dogs). Since the amount of time ‘working’ was highly variable, we quantified activity levels using the DORA questionnaire, rather than by owner-reported role. Most of the dogs had undergone gonadectomy (female = 82, male = 75) but some were sexually intact (female = 26, male = 58). BCS ranged from 3 (slightly underweight) to 9 (severely overweight) with mean BCS 5.7 (SD = 1.3). Weight ranged from 17 to 59 kg (mean = 32.7kg, SD = 7.0). Further information, including summary statistics in different sub-groups of Labradors, is presented in Table S2.

Direct genotyping was performed on the CanineHD Genotyping BeadChip (Illumina) array and data were then imputed to 9.4 million single nucleotide polymorphisms (SNPs) against a reference panel of genomes from 676 dogs of 91 breeds, including 31 Labrador retrievers. For the GWAS, we retained SNPs called with 70% confidence and which were called in >95% of dogs with an allele frequency > 5% and a Hardy Weinberg equilibrium test *p* >0.001%. There were 4.5 million SNPs included in the GWAS.

### Canine GWAS for body condition score

**Fig. 1:**
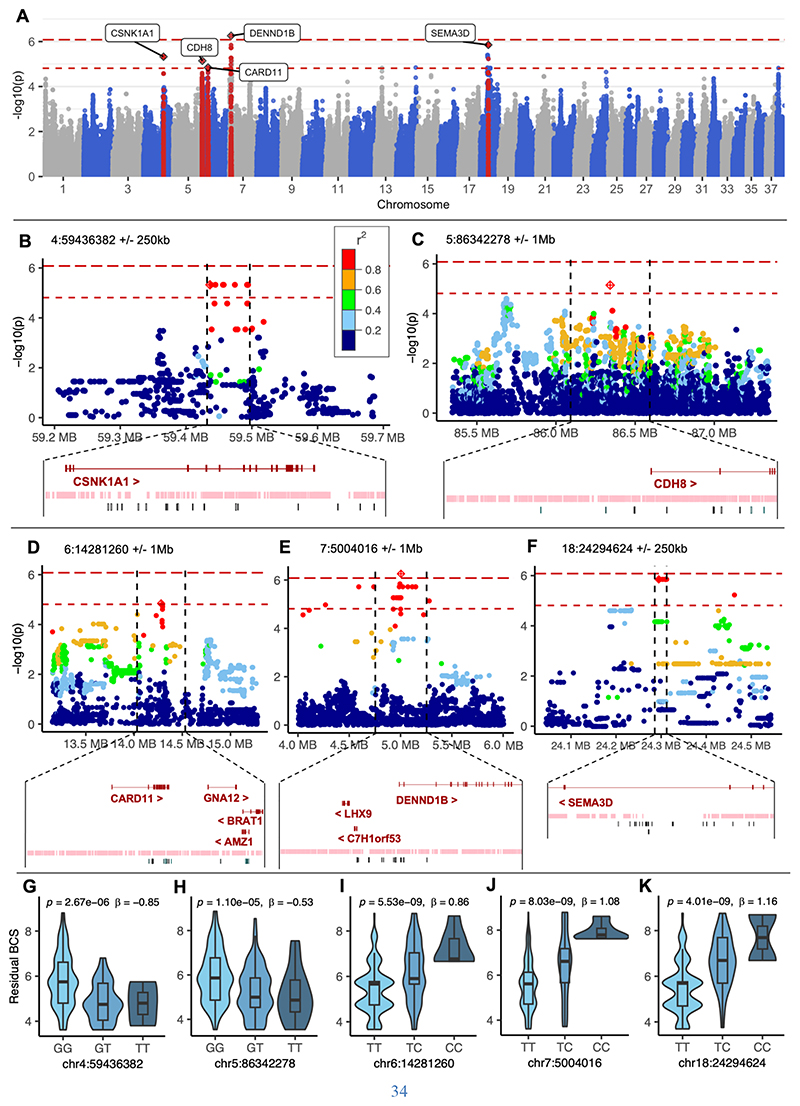
GWAS in Labrador retrievers identifies multiple obesity genes. (A) Manhattan plot for GWAS for body condition score (BCS) in Labrador retrievers (n = 241). Suggestive significance shown with open dashes, Bonferroni-corrected significance *p* < 8.31x10^-7^ shown with closed dashes. Five independent loci which harbored protein coding genes are labelled with the most proximal protein coding genes (lead SNP at 4:59436382, 5:86342278, 6:14281260, 7:5004016, 18:24294624). Regional Manhattan plots shown for (B) Chr. 4, (C) Chr. 5, (D) Chr. 6, (E) Chr. 7, (F) Chr. 18 are colored by *r*^2^ measure of linkage disequilibrium. For chromosomes 4 and 18, the plot extends +/-250kb of the lead SNP and the funnel indicates the boundaries of the mapped haplotype. For other regions, plots extend +/-1Mb and genes in the funnel are those lying +/-250kb of the lead SNP. The lead SNP for each locus is indicated by a diamond, with genes within the region annotated below. Similarity with human genome is indicated by a LASTZ pairwise alignment with GRCh38 shown as the pink track. Variants identified from WGS as segregating with the lead SNP ≥70% of the time are aligned in black below. Partial regression violin plots showing relationship between BCS and lead variant genotype detail large effect sizes at each locus on (G) Chr. 4, (H) Chr. 5, (I) Chr. 6, (J) Chr. 7, (K) Chr. 18. Chr., Chromosome.

We performed a GWAS for BCS in 241 Labrador retriever dogs applying a linear mixed effects model (GCTA MLMA-LOCO). Regression modelling was used to identify factors significantly affecting BCS in the population which were then included as covariates for the GWAS. These included sex, neuter status, and sex:neuter status interaction term (Fig. 1) (28). Our stringent Bonferroni corrected significance threshold (*p* = 8.31x10^-7^) was surpassed by one variant within the gene *DENND1B*, rs24430444. A more lenient nominal significance threshold was determined by the point at which the observed versus expected *p* value diverged outside the 95% confidence interval on a quantile-quantile (QQ) plot, an approach previously applied in canine GWAS studies (29, 30) (Fig. S2). This threshold of *p* = 1.54x10^-5^ was surpassed by a further 109 SNPs (Fig. 1A). Heritability of BCS in this canine cohort, measured using GCTA-LDMS GREML analysis from GCTA, was estimated at 70% (+/-22%).

Conditional analysis identified seven independent, non-overlapping, signals surpassing the suggestive significance threshold. Haplotype mapping and LD structure was used to define regions of interest ranging from 4.5 kb – 2.2 Mb long (mean = 549 kb, median = 65 kb, Table S3), of which five contained protein coding genes (Fig. 1B-K). Further information about lead SNP at each locus is detailed in the Supplementary Text. Three regions contained just one protein coding gene (*CSNK1A1, SEMA3D* and *CDH8*). At the chromosome 6 locus there were two genes (*SDK1, CARD11*) with the lead SNP positioned within an intron of *CARD11*. At the chromosome 7 locus there were seven genes (*NR5A2, PTPRC, ATP6V1G3, NEK7, LHX9, DENND1B, CRB1*) with the lead SNP positioned within an intron of *DENND1B*. We interrogated Labrador whole genome sequences across each locus in dogs carrying both risk and non-risk alleles to search for genetic variants which might be considered candidates for causation. Multiple non-coding variants were identified which are listed in Table S4; no protein coding mutations predicted to have a deleterious consequence were identified and the canine LD structure meant no single causative variant could be defined. None of the genes within these loci were previously well characterized as having roles in obesity, although some had epidemiological or functional data to suggest a plausible role in energy homeostasis (detailed in Table S5).

### Canine obesity genes are implicated in human obesity

To test if regions and genes identified on the canine GWAS were also relevant to human obesity, we identified regions of the human genome that were syntenic to the regions of interest defined in dogs, and examined for BMI association with all annotated genes within those human regions. We hypothesized that if canine candidate genes also regulate human BMI there would be a statistically significant association implicating the gene in one or more of the following analyses: a GWAS for BMI on 806,834 participants from the GIANT study (31); an exome-wide association study (ExWAS) of rare (MAF < 0.1%), deleterious exome variants from 454,787 individuals from the UK Biobank study (UKB) (32–34); and rare variant enrichment tests in the Severe Childhood Onset Obesity Project (SCOOP-UK) (35) (n = 982), specifically testing for enrichment of very rare (MAF < 0.0026%), predicted deleterious (CADD ≥ 25) variants compared to reference exomes of similar ancestry (gnomAD v2.1.1, n = 56,885) (36).

Furthermore, we investigated the Severe Obesity in Pakistani Population (SOPP) cohort which includes patients who presented with severe, early onset obesity and in whom no monogenic causes of obesity were identified with exome sequencing. Since SOPP patients have normal weight parents and come from a highly consanguineous population, they are likely enriched for homozygous carriers of as yet unknown genetic causes of obesity.

**Fig. 2:**
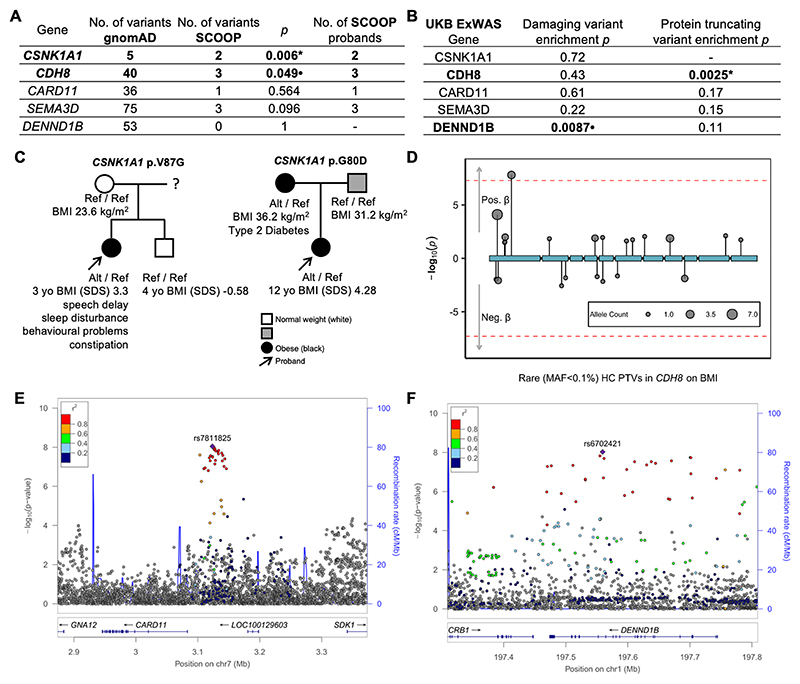
Canine obesity-associated genes are also associated with human obesity. (A) *CSNK1A1* and *CDH8* are enriched for rare, deleterious (gnomAD minor allele count ≤ 3, CADD ≥ 25) variants in the SCOOP cohort of 982 children with severe, early onset obesity compared to 56,885 controls from gnomAD (one sided Fisher’s exact test). (B) ExWAS analysis on exome sequences of ~500k individuals in UK Biobank showed rare protein truncating variants in *CDH8* were associated with BMI and damaging (protein truncating and high CADD) variants in *DENND1B* were nominally associated with BMI (Fig. S3). (C) Genotype segregated with obesity phenotype in pedigrees from families of probands with severe, early onset obesity for two severe, deleterious variants in *CSNK1A1* identified in SCOOP and *DENND1B* p.R501C identified in SOPP (Fig. S3). (D) Lollipop plot shows protein truncating variants in *CDH8* were associated with increased BMI in the UKB ExWAS. Regional Manhattan plots for (E) *CARD11* and (F) *DENND1B* show that in a GWAS of ~800k individuals there were associations with BMI at two human loci orthologous to canine GWAS loci, at which these were called as the likely effector genes using the GWAS2Gene pipeline. Significance: *p* < 0.00023, ‘*’; nominal *p* < 0.05, ‘•’; no variants, -. PTV - protein truncating variants; HC – high confidence; MAF – minor allele frequency; SCOOP - Severe Childhood Onset Obesity Project.

Using this approach, we identified evidence of a genetic association with human obesity for all of the five top canine loci (Fig. 2, Table S6, S7). Full details, including clinical descriptions of the patients identified, are included in the Supplementary Text but, in brief, *CARD11* was associated in the GIANT GWAS for BMI; *CSNK1A1* was enriched for rare, deleterious variants in SCOOP with variants segregating with obesity in two families; *CDH8* was enriched for rare, deleterious variants in both SCOOP and UKB; and a proband with a rare, predicted deleterious homozygous *SEMA3D* mutation was identified in SOPP. Multiple approaches showed a human *DENND1B* association, as expanded below.

### DENND1B is associated with canine and human obesity

The top canine association was within the *DENND1B* gene which encodes DENN Domain Containing 1B, a guanine nucleotide exchange factor for Rab35 that binds to the adaptor protein 2 (AP2) complex, and has a critical role in clathrin-mediated endocytosis of membrane proteins (37). Each allele of the intronic 7:5004016:T>C variant in dogs conferred a 0.94 increase in BCS (Fig. 1J). This association was replicated in regression modelling of its effect in a large population of golden retrievers (n = 1793; n = 2229) for BCS (*p* = 0.029) and body weight (*p* = 0.0022).

Canine *DENND1B* has high homology with human (89.4%) and mouse (82.7%) orthologues, particularly in functionally important domains (Fig. S3). In humans, GWAS on 806,834 participants from the GIANT study (31) showed significant association with BMI within the human region syntenic to the canine association signal (Table S7). The lead signal rs6702421 (0.011 kg/m^2^ increase per copy of the T allele, 24% frequency, *p* = 9.42x10^-9^) is intronic to *DENND1B* (Fig. 2F), while the secondary signal rs1009188 (0.012 kg/m^2^ increase per copy of T allele, 71% frequency, *p* = 7.15x10^-11^) is further upstream. We used activity-by-contact (ABC) enhancer maps (38) to identify whether these GWAS SNP or their proxies fell within regulatory elements for any of their proximal genes (promoters/enhancer/etc.), restricting our investigations to tissues where our candidate genes were actively expressed.

For the *DENND1B* signal, rs6702421, we found that SNPs in high LD with the signal (*r*^2^ > 0.8) lay within two regulatory elements identified by the ABC enhancer maps. One was the DENND1B promoter itself as identified using the HAP1 human cell line from ENCODE. The other was an enhancer element active in bipolar iPSC neurons from ENCODE. Colocalization analyses using expression QTL data showed alleles for decreased DENND1B expression in blood also associated with decreased BMI (Table S7). This corroborates the hypothesis that the BMI GWAS signal and its closely correlated SNPs alter the sequence of established enhancers of DENND1B and consequently the expression of DENND1B.

All of those data were integrated as part of the GWAS 2 Gene (G2G) pipeline (39),and further information in supplementary methods) which predicted *DENND1B* as the most likely causal gene at this locus and in the 96th centile of likely causal prioritized genes in the BMI GWAS (Table S7). Furthermore, rare damaging variants in *DENND1B* are nominally associated with BMI in UKB (*p* = 0.0087, β = 0.35 kg/m^2^, Fig. 2B, Fig. S4).

### DENND1B is co-expressed with hypothalamic receptors involved in energy homeostasis

Since DENND1B has a role in clathrin mediated endocytosis of signaling receptors (40), we hypothesized that variation in DENND1B activity would affect the internalization, cell surface expression and/or recycling of receptors involved in energy homeostasis. We focused initially on *MC4R*, mutations in which cause human obesity (23). Canine RNAseq data from BarkBase (41) confirmed *DENND1B* is expressed in the canine brain (cortex 0.26, cerebellum 0.27, and pituitary 1.01 fragments per kilobase of transcript per million read pairs, other brain regions not available) (Table S8).

**Fig. 3:**
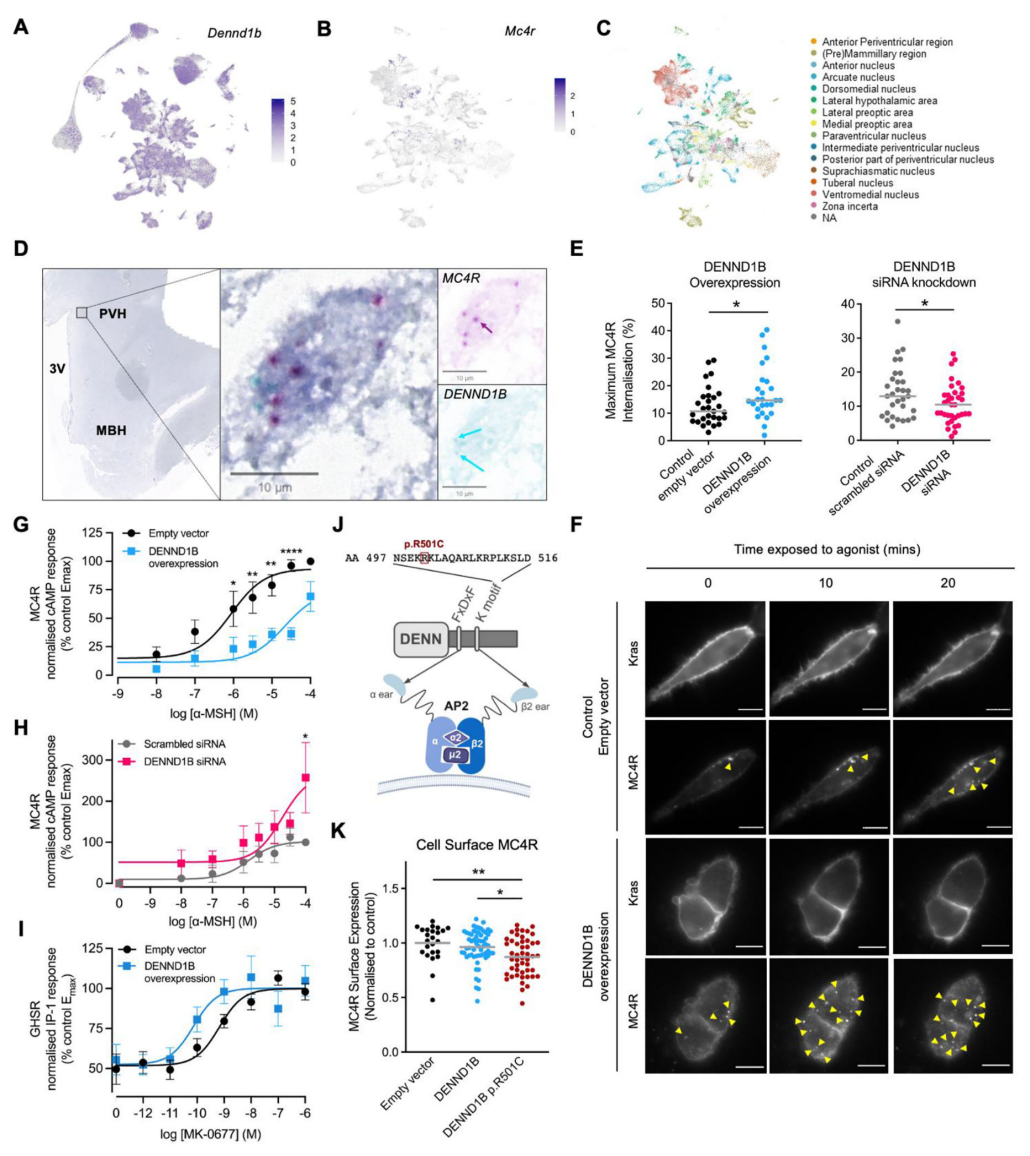
*DENND1B* is co-expressed with *MC4R* and regulates its signaling. (A) UMAP plots showing log-normalized expression of (A) *Dennd1b* in murine hypothalamus and (B) *Mc4r* in the subset of *Dennd1b* positive neurons, highlighting co-expression. (C) *Dennd1b* positive neurons colored by hypothalamic region. (D) Coronal section of human hypothalamus stained for *MC4R* (red) and *DENND1B* (green) showing dual positive neurons within PVH. (E) DENND1B overexpression (blue) enhances ligand-stimulated MC4R internalization, while *DENND1B* siRNA knockdown (pink) reduces it. (F) HILO images of HEK293 expressing MC4R and plasma membrane marker Kras showing colocalization at baseline and increased MC4R internalization on ligand stimulation with *DENND1B* overexpression. Scale 5µm. Ligand-induced G_s_ cAMP response downstream of MC4R in HEK293 cells is (G) reduced by *DENND1B* overexpression and (H) increased at maximal concentrations by *DENND1B* knockdown. (I) Overexpressing DENND1B causes increased signaling in the canonical IP-1 pathway downstream of GHSR. (J) DENND1B contains both the normal DENN protein AP-2α ear-binding motif (FxDxF) and an AP-2β_2_ ear-binding motif whose sequence is shown with *DENND1B* p.R501C highlighted. (K) Cell surface expression of MC4R during expression of DENND1B wild type, p.R501C, and empty vector. Significance: *p*≤0.05 ‘*’, *p*≤0.01 ‘**’, *p*≤0.0001 ‘****’. MBH, mediobasal hypothalamus; 3V, third ventricle; PVH, Paraventricular hypothalamic nucleus. Figure 3J created using BioRender.com.

To look at co-expression of *DENND1B* and *MC4R* in the hypothalamus, we interrogated HypoMap: a unified single cell gene expression atlas of the mouse hypothalamus (42), and HYPOMAP: A comprehensive spatio-cellular map of the human hypothalamus (43). We found high *DENND1B/Dennd1b* expression in hypothalamic neuronal clusters (with lower expression levels in non-neuronal cell types) in all regions of the hypothalamus, including in the paraventricular nucleus of the hypothalamus (PVH) (Fig. 3A-C, Fig. S5, S6). In mouse, *Dennd1b* was expressed in 22.2% of all *Mc4r*-expressing cells (Fig. 3B, C, Table S9). In humans, *DENND1B* was expressed in 79.9% of *MC4R*-expressing neurons and expressed in 63.3-87.5% of cells in the 5 clusters with the highest percentages of *MC4R* expression (Fig. S6, Table S9, S10). Duplex RNAscope in situ hybridization in human hypothalamic tissue sections confirmed *DENND1B/MC4R* co-expression in neurons within the PVH. (Fig. 3D).

In addition to MC4R we examined multiple other hypothalamic receptors with known roles in energy homeostasis. In both the murine (Fig. S5) and human (Fig. S6) hypothalamus, there was co-expression of *DENND1B/Dennd1b* with growth hormone secretagogue receptor (*GHSR*), melanocortin 3 receptor (*MC3R/Mc3r*), Neuropeptide Y Receptors Y1 and Y5 (*NPY1R/Npy1r, NPY5R/Npy5r*), leptin receptor (*LEPR/Lepr*), insulin receptor (*INSR/Insr*), 5-Hydroxytryptamine Receptors 1B and 2C (*HTR1B/Htr1b, HTR2C/Htr2c*), and glucagon like peptide 1 receptor (*GLP1R/Glp1r*). We included GHSR in functional studies to examine DENND1B activity as its orexigenic effect contrasts with the anorexigenic effect of MC4R.

### DENND1B expression affects internalization and signaling of MC4R

To test whether DENND1B expression affects signaling or receptor internalization of MC4R and GHSR, receptors were overexpressed in HEK293 cells and ligand-induced cAMP generation and internalization were assessed under conditions of DENND1B overexpression or *DENND1B* knockdown and compared to control conditions (empty vector or scrambled siRNA, respectively). Neither condition affected basal cell surface expression of MC4R (Fig. S7).

However, overexpression of *DENND1B* increased MC4R internalization after ligand activation and reduced cAMP signaling (Fig. 3E, F, G). Conversely, knockdown of *DENND1B* reduced MC4R internalization and increased cAMP signaling although only at maximal ligand concentrations (Fig. 3E, H, Fig. S7).

In contrast, altering *DENDD1B* expression had no effect on GHSR cell surface expression, cAMP signaling or internalization (Fig. S8). However, *DENDD1B* overexpression did increase signaling (reduced pEC_50_) by the canonical IP-1 pathway downstream of GHSR (Fig. 3I, Fig. S7C).

### A human DENND1B missense variant in a morbidly obese patient affects MC4R expression

A patient with severe childhood obesity was identified in the SOPP cohort as homozygous for a *DENND1B* p.R501C (Fig. S4B). This variant is extremely rare, with only a single heterozygous carrier found in gnomAD (MAF = 6.7 × 10^-6^, Table S11). No alternative genetic diagnosis for variants in established candidate obesity genes was identified by exome sequencing (44, 45). The proband presented at 2.4 years of age with body weight of 32 kg (BMI 32, BMI standard deviation score, SDS, 7.01) accompanied by hyperphagia. At 7 years of age, she weighed 63 kg (BMI 34.5, BMI SDS 4.72). Neurodevelopmental milestones were normal. Both parents were heterozygous and did not have obesity. This variant has a CADD Score of 23.9 and is predicted to affect a binding motif that interacts with AP2 (Fig. 3J), a key binding partner of DENND1B at the initiation of endocytosis (40).

We tested the functional effect of *DENND1B* p.R501C in vitro as above. It caused a reduction in MC4R protein abundance at the cell surface, compared with both the empty vector (*p* = 0.006) and wildtype *DENND1B* (*p* = 0.013, Fig. 3K). Additionally, it caused a reduction in cAMP response to ligand activation of MC4R compared to the empty vector although to a lesser extent than wildtype *DENND1B* (*p* ≤ 0.0001, Fig. S7E).

### Polygenic risk score to quantify obesity risk in dogs

**Fig. 4:**
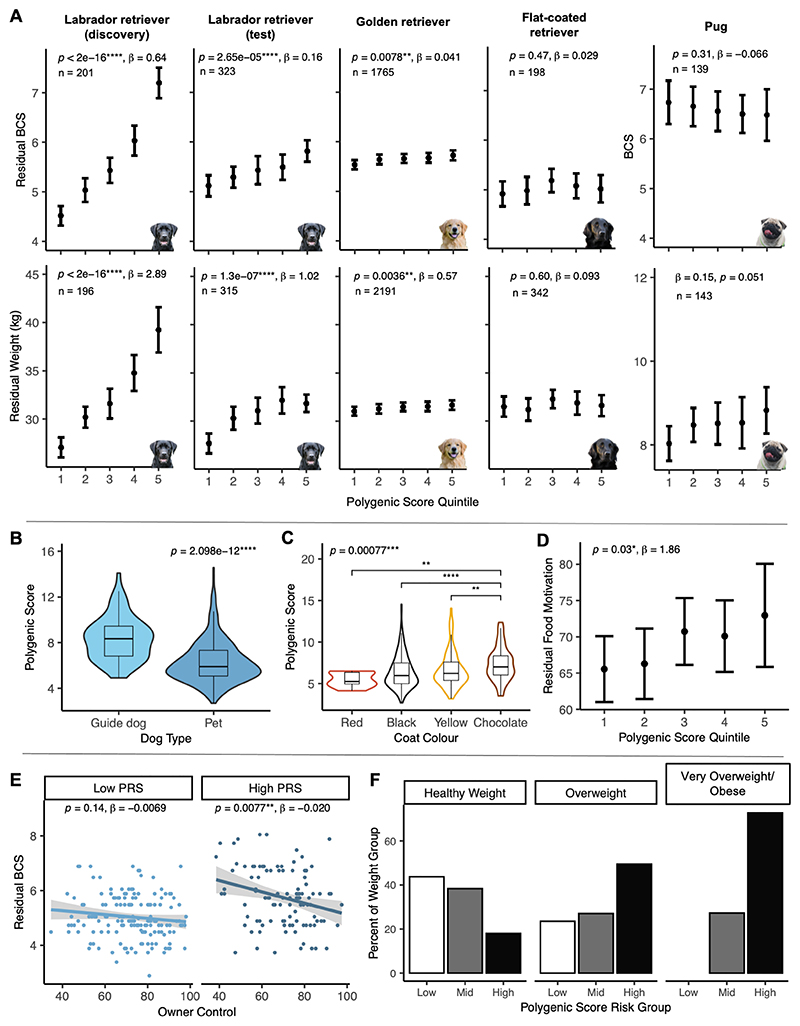
Polygenic risk scores (PRS) predict obesity and provide insight into complex trait expression. A PRS was constructed comprising 16 SNPs weighted for GWAS effect size. (A) The PRS predicted BCS and weight in independent populations of Labrador retrievers. In golden retrievers, the PRS constructed in Labradors predicted BCS and weight, albeit less strongly. In more distantly related breeds, flat-coated retrievers and pugs, the PRS had no predictive value. Known risk factors for obesity in the Labrador population were explained by differences in PRS, with (B) higher PRS in obesity-prone assistance dogs and (C) chocolate-colored Labradors. (D) The PRS predicted food motivation score in Labrador retrievers (n = 298). (E) Gene-environment interaction: dogs with low PRS were resistant to obesity irrespective of owner control of diet and exercise, but management of dogs with high PRS does significantly affect obesity outcome (significant in regression model, *p* = 0.0077). (F) Prevalence of high PRS dogs increased with obesity category (healthy BCS < 6/9; overweight BCS 6-7/9; very overweight BCS >7/9) and there were no low-risk dogs in the extremely overweight group (PRS grouped by tertiles). Significance levels: *p* ≤ 0.05 ‘*’, *p* ≤ 0.01 ‘**’, *p* ≤ 0.001 ‘***’, *p* ≤ 0.0001 ‘****’.

Polygenic risk scores (PRS) have not previously been applied in dogs. We constructed a PRS comprising 16 SNPs weighted for GWAS effect size on BCS using the ‘clumping and thresholding’ technique (Fig. S9) (46). The PRS improved prediction of BCS and body weight in an independent set of Labrador retrievers (Fig. 4A). When we included PRS, the model predicted 11% of the variability in BCS compared to just 4.5% when PRS is not included (Table S12).

We applied the PRS to determine its utility in other breeds, to test whether it explained known obesity risk factors in the breed and to examine how genetic risk interacted with dogs’ environmental exposure to food and exercise (Fig. 4B-F and Supplementary Text). The Labrador PRS retained a small but significant predictive value for BCS and body weight in a closely related breed, the golden retriever (*p* = 0.0078, β = 0.0041, n = 1765), in which adding PRS to the model predicted 7.4% of the variability in BCS compared to 7.1% when it was not included. PRS was not predictive in more distantly related breeds (Supplementary Text, Fig. 4A, Table S13). We also observed that in dogs with high polygenic risk, stricter owner control of diet and exercise significantly reduced BCS (*p* = 0.0077) but that it had no statistically significant impact on BCS in dogs with low polygenic risk (Fig. 4E).

## Discussion

A canine GWAS for body condition score in Labrador retrievers identified multiple genes associated with human obesity. The genes have previously not been well studied for their effect on energy homeostasis because the association has not been reported or their effect size in humans is small. In dogs, large effect sizes provide orthogonal evidence these genes can strongly influence energy homeostasis and are worthy of more in-depth study.

The lead canine GWAS signal was at *DENND1B* for which we identified a role in the regulation of hypothalamic melanocortin signaling. Human genomics revealed significant associations between *DENND1B* and BMI using both common (GWAS) and rare variant (ExWAS) approaches. Furthermore, we studied the molecular consequences of a mutation implicated in causing severe, early onset obesity in a single homozygous proband. In dogs, we generated a common variant PRS which provided multiple insights into known within-breed differences in obesity susceptibility, as well as evidence of gene-environment interaction in the regulation of body fat mass.

*DENND1B* variants were associated with obesity in both dogs and humans. Previously, this gene has been implicated in the pathogenesis of childhood asthma and other immune disorders by modifying T cell receptor function (47). Based on its previously characterized role in clathrin-mediated endocytosis (48), we hypothesized that DENND1B may regulate the trafficking and consequently the signaling of MC4R and GHSR.

Our data show DENND1B promotes MC4R internalization and reduces cAMP mediated anorexigenic signaling downstream of the receptor, suggesting that DENND1B can regulate MC4R trafficking and signaling, with the proposed mechanism summarized in Fig. S10. This finding is consistent with the human genetic evidence that the protective allele at the *DENND1B* locus is associated with reduced expression of the gene. Since even minor alterations in MC4R activation have been shown to have a clinically observable effect, this would be consistent with altering obesity risk (49, 50). Functionally deleterious mutations in two other regulators of MC4R signaling cause human obesity, the chaperone protein MRAP2 and transcription factor SIM1 (51, 52).

DENND1B also caused increased IP-1 signaling by the orexigenic receptor GHSR. This remains consistent with the human genetic findings. The finding is reminiscent of how other regulatory proteins, notably MRAP2, regulate the signaling and trafficking of multiple G protein-coupled receptors (GPCRs) (53) and may suggest a role for DENND1B in orchestrating a wider repertoire of responses in energy homeostasis.

In a single morbidly obese human patient, we identified a deleterious homozygous *DENND1B* missense variant. Overexpression of the variant reduced cell surface expression of MC4R as compared with wild type DENND1B, consistent with previous findings that most obesity-associated *MC4R* mutations reduce cell surface expression (49). The variant caused a lesser reduction in cAMP accumulation after ligand activation of MC4R compared to wild type DENND1B. This suggests that, in common with ~25% of obesity-associated *MC4R* mutations, it may not cause obesity by impairing the canonical Gs-cAMP pathway. Instead, such *MC4R* mutations can impact receptor homodimerization, recycling or alternative signaling pathways (for example, ERK1/2 phosphorylation). This *DENND1B* variant may have similar complex effects, or effects on other GPCRs which warrant further investigation.

Our work advances the understanding of the genetics of obesity in dogs. The PRS provided a meaningful increase in predictive value of BCS in Labradors over conventional risk factors (6-7%), which was comparable to the predictive value of polygenic scores developed specifically for human BMI (54–56). Its utility was shown to be restricted to the discovery breed, which is expected given that LD structure varies across breeds (57). Even so, it is important to highlight this at a time when canine disease prediction is increasingly desired and discussed in veterinary medicine.

Notably, the *POMC* p.P187fs variant was not statistically significantly associated with BCS in the GWAS. This may be due to variant stratification within the population (it is more common in assistance dogs which were not included in the discovery GWAS but were included in the original research reports of this mutation), low allele frequency (MAF 0.14), modest effect size, and variable penetrance in the study dogs, illustrating the complex genetic architecture of canine obesity.

We showed stratification of genetic risk exists even within the breed, with previously recognized risk factors - chocolate coat color and being purpose bred for assistance work – being associated with higher polygenic risk which was reflected by varying degrees of genetic stratification. The high polygenic risk in the genetically distinct assistance dog population of Labradors is reminiscent of the high frequency of the *POMC* p.P187fs variant in the same cohort (19). This may be due to genetic drift but raises the possibility of inadvertent selection for obesity-promoting genetic variants in this population, perhaps because dogs with a high food drive are easier to train using food to positively reinforce desirable behavior, meaning they are more likely to be selected for breeding future generations of assistance dogs.

Polygenic risk was shown to be mediated in part via eating behavior in dogs, as in other species, measured as food motivation score using a validated questionnaire (24). This means dogs with higher polygenic risk were more likely to seek out food in the home environment, to ‘beg’ for food, and to eat any food on offer. Labradors with low polygenic risk tended to remain normal weight irrespective of owner control of diet and exercise, but high-risk dogs were prone to developing obesity if dog activity was limited and owners were permissive with food (for instance, by offering human food or not restraining their dogs’ intake by limiting the food available). These canine data provide a compelling illustration of gene-environment interaction and supports data from human populations that show individuals with high appetite are particularly vulnerable to developing obesity in an permissive environment and so need to exercise greater cognitive restraint to maintain a healthy body weight (58, 59).

We have identified obesity-related genes in humans by studying the canine model, with findings relevant to preventative and therapeutic interventions in both species. The discovery of DENND1B as a regulator of MC4R activity informs our understanding of melanocortin signaling, a critical pathway in hypothalamic regulation of energy homeostasis. Importantly, our findings show that even high polygenic risk can be mitigated. These findings demonstrate the benefits of studying complex disease in non-traditional animal models such as the dog and have practical implications for improved management of canine obesity.

## Materials and methods summary

The materials and methods are summarized here, and further detail is found in the supplementary materials document.

Canine and human research was approved by the relevant local ethical review committees and the appropriate consent obtained. We studied pet and working adult Labrador retriever dogs (age 1-10 years), free of systemic illness, not being treated with medications likely to affect obesity status, and which lived with their owners. Body fat mass was assessed using a well validated measure of adiposity, Body Condition Score (BCS) which uses visual and haptic descriptors to score dogs from 1-9 where 4-5 represents optimal body fat mass and 8-9 is considered obese (Fig. S1) (25–27). Food motivation and owner control of diet and exercise was determined using the validated, owner-reported Dog Obesity Risk Assessment questionnaire (DORA) which scores responses to a series of statements about dog behavior related to food, and owners’ management of their dog’s diet, and dogs’ activity levels (Table S1 and Supplementary Methods) (24). Canine DNA samples were extracted from saliva collected using oral swabs (Performagene, DNA Genotek) or from residual EDTA blood samples left over after veterinary investigation (Qiagen, UK). Direct genotyping was performed on the CanineHD Genotyping BeadChip (Illumina) array and data were imputed to 9.4 million single nucleotide polymorphisms (SNPs) against a reference panel of genomes from 676 dogs of 91 breeds, including 31 Labrador retrievers.

We performed a GWAS for BCS in 241 Labrador retriever dogs using the 4.5 million SNPs retained after data quality control. To identify factors significantly affecting BCS in the study population we performed regression modelling, using Akaike’s Information Criterion to identify the minimal model. Significant factors in the regression (sex, neuter status and a sex:neuter interaction term) were included as covariates in the GWAS which applied a linear mixed effects model (GCTA MLMA-LOCO) to identify variants associated with BCS (60). A stringent, conservative significance threshold (*p* = 8.31x10^-7^) was determined by Bonferroni correction, using the number of independent SNP in the analysis (determined by LD pruning of the data set using a cut-off of *r*^2^ < 0.7 in PLINK v.1.9) (61). A more lenient nominal significance threshold was determined (*p* = 1.54x10^-4.81^) by the point at which the observed versus expected p value diverged outside the 95% confidence interval on a quantile-quantile (QQ) plot, an approach previously applied in canine GWAS studies (29, 30). Heritability of BCS was estimated using GCTA-LDMS GREML (60, 62, 63). Stepwise conditional analysis was performed to identify independent signals followed by haplotype mapping and LD structure (*r*^2^ ≥ 0.8 with the lead SNP) analysis to define regions of interest PLINK v.1.9 (61). Each locus was interrogated in whole genome sequences from Labrador dogs carrying both risk and non-risk alleles to search for genetic variants which might be candidates for causation.

Canine polygenic risk scores (PRS) were constructed using GWAS SNP weighted by effect size on BCS, using the ‘clumping and thresholding’ technique to include only independent variants from loci most strongly associated with BCS (Fig. S9) (46). A secondary test set of Labrador retrievers was genotyped by low-pass sequencing with imputation using skimSEEK™ technology (Neogen Europe Ltd). We tested whether PRS was a predictor of BCS, weight or food motivation in the test set of Labradors and dogs of other breeds (flat-coated retrievers, pugs and golden retrievers), and if it was associated with known obesity risk factors (coat color, assistance dog status). To examine how genetic risk interacted with environmental exposure to food and exercise, we modelled the predictive effect of owner control of diet and exercise (measured using the DORA questionnaire) on BCS for dogs of contrasting PRS.

To determine whether loci and genes identified on the canine GWAS were also relevant to human obesity, we identified human genome loci syntenic to the regions of interest defined in dogs. To test whether genes in those regions were associated with human obesity, we examined whether there was a statistically significant association with BMI in both large population based studies and in cohorts of patients with severe, early onset obesity. We interrogated data from a human GWAS for BMI in 806,834 participants from the GIANT study (31) and used the GWAS 2 Gene (G2G) pipeline (39) to identify independent GWAS signals and predict causal genes for human GWAS associations at each locus. Additionally, in 454,787 individuals from the UK Biobank study (UKB) (32–34), we performed an ExWAS study, implementing BOLT-LMM v2.3.551(64) and using a set of dummy genotypes representing the per-gene carrier status for rare (MAF < 0.1%), deleterious exome variants.

Focusing on patients with severe, early onset obesity, we first analyzed data from the Severe Childhood Onset Obesity Project (SCOOP) (n = 982), a subset of the Genetics of Obesity Study (GOOS) consisting of patients who presented with severe obesity in childhood, all of UK British origin (35). Specifically, we tested for enrichment of very rare (MAF < 0.0026%), predicted deleterious (CADD ≥ 25) variants compared to reference exomes of similar ancestry (gnomAD v2.1.1, n = 56,885 (36). We also investigated the Severe Obesity in Pakistani Population (SOPP) which is comprised of individuals for which selection criteria include having a BMI >35 or BMI SDS (standard deviation score/Z score compared to WHO global reference data (65, 66) for age) >3.5; onset of obesity prior to 5 years of age; pronounced hyperphagia; and having parents with either first- or second-degree consanguinity who are of normal weight or overweight (explicitly excluding parental obesity). We hypothesized that affected probands would be homozygous for deleterious variants in the canine genes of interest. Where variants were identified, we examined their frequency in ancestry diverse public comparator populations including gnomAD v.2.1.1, NCBI (67), TopMED (68), and NIH ClinVar (69).

We examined the expression of genes of interest by analyzing canine RNAseq data from BarkBase (41). To test their expression in brain regions important in energy homeostasis and to find out if they were co-expressed receptors involved in neuroendocrine control of body weight, we interrogated data from HypoMap: a unified single cell gene expression atlas of the mouse hypothalamus (42), and HYPOMAP: A comprehensive spatio-cellular map of the human hypothalamus (43). RNAscope in situ hybridization in human hypothalamic tissue sections was performed as previously described (70) to confirm co-expression of *DENND1B*, with *MC4R*.

To test the effect of DENND1B on the function of hypothalamic receptors, we performed molecular experiments in HEK293 cells, cultured as previously described (71). Specifically we tested MC4R, mutations in which cause obesity, and GHSR, a contrasting orexigenic receptor (23). Briefly, SNAP-tagged receptors (MC4R and GHSR) were transiently transfected using Lipofectamine 2000 (LifeTechnologies) in combination with DENND1B overexpression (WT or DENND1B p.R501C) or knockdown (siRNA). After forty-eight hours, endogenous surface expression of SNAP-647 (NEB) labelled receptor was determined by co-localization (JACoP) (72) with co-expressed Venus-Kras using live HILO microscopy. Subsequently, receptor translocation away from the membrane was measured after ligand activation using the same technique. Receptor surface expression was compared using a one-way ANOVA and receptor internalization using a Mann-Whitney test. Ligand-induced cAMP generation was assessed using the cAMP GloSensor assay (Promega) after co-expression with pGloSensor-22F. Ligand-induced GHSR canonical signaling was measured using the Cisbio IP-One Gq HTRF kit (Revvity, Codolet, France). For MC4R assays, α-MSH (Bachem AG) was added (100 μM – 10 nM). For GHSR assays, high affinity agonist MK-0677 (Tocris, Abingdon, UK) was added (10μM - 10nM). For all concentration-response curve assays, pEC50 values from independent experiments were grouped, normalized and compared by one-way ANOVA.

## Supplementary Material

Supplementary Materials

## Data Availability

All genetic and phenotypic data relating to this project are available on Dryad (73) with the exception of the GRLS data which are available from the GRLS data commons site (https://datacommons.morrisanimalfoundation.org/). Code used for imputation is available on Zenodo (74). Research materials can be provided by E.R. pending scientific review and a completed material transfer agreement. Requests for materials should be submitted to E.R. (er311@cam.ac.uk).
