# Supplementary material for: Canine genome-wide association study identifies DENND1B as an obesity gene in dogs and humans
Source: Science. Author manuscript; Available in PMC 2026 Feb 2. (PMC7618706; doi:10.1126/science.ads2145)
Supplement: Supplementary Materials [file EMS211953-supplement-Supplementary_Materials.zip › science.ads2145_mdar_reproducibility_checklist.pdf]

Materials Design Analysis Reporting (MDAR)
Checklist for Authors

The MDAR framework establishes a minimum set of requirements in transparent reporting applicable to studies in the life sciences (see Statement of Task: doi:10.31222/osf.io/9sm4x.). The MDAR checklist is a tool for authors, editors and others seeking to adopt the MDAR framework for transparent reporting in manuscripts and other outputs. Please refer to the MDAR Elaboration Document for additional context for the MDAR framework.

Materials

Antibodies	Yes (indicate where provided: page no/section/legend)	n/a
For commercial reagents, provide supplier name, catalogue number and RRID, if available.		X

Cell materials	Yes (indicate where provided: page no/section/legend)	n/a
**Cell lines:** Provide species information, strain. Provide accession number in repository **OR** supplier name, catalog number, clone number, **OR** RRID	Supplementary materials: Methods, Cell culture and transfection, pg. 10	
**Primary cultures:** Provide species, strain, sex of origin, genetic modification status.		X

Experimental animals	Yes (indicate where provided: page no/section/legend)	n/a
**Laboratory animals:** Provide species, strain, sex, age, genetic modification status. Provide accession number in repository **OR** supplier name, catalog number, clone number, **OR** RRID		X
**Animal observed in or captured from the field:** Provide species, sex and age where possible	Labrador data collection and ethics outlined in Supplementary Materials and Methods, pg. 2. Further data collection outlined in pg. 5-6.<br>Raw phenotypic data for all the participant dogs is outlined in the data availability section, including sex and age information.	
**Model organisms:** Provide Accession number in repository (where relevant) **OR** RRID		X

Plants and microbes	Yes (indicate where provided: page no/section/legend)	n/a
**Plants:** provide species and strain, unique accession number if available, and source (including location for collected wild specimens)		X
**Microbes:** provide species and strain, unique accession number if available, and source		X

Human research participants	Yes (indicate where provided: page no/section/legend)	n/a
Identify authority granting ethics approval (IRB or equivalent committee(s), provide reference number for approval.	Supplementary materials, Ethics statement: Humans, pg. 2:	
Provide statement confirming informed consent obtained from study participants.	Supplementary materials, Ethics statement: Humans, pg. 2:	

Report on age and sex for all study participants.	Age and sex for human study participants can be accessed via the original data sources, as detailed in the data availability section below.<br><br>For the SCOOP and SOPP severely obese human probands, age and sex for participants is referred to directly in the main text (page 7), Supplementary text (page 19), and in supplementary table S13.	

Design

Study protocol	Yes (indicate where provided: page no/section/legend)	n/a
For clinical trials, provide the trial registration number **OR** cite DOI in manuscript.		X

Laboratory protocol	Yes (indicate where provided: page no/section/legend)	n/a
Provide DOI or other citation details if detailed step-by-step protocols are available.	Supplementary material file, Materials and methods section, outlines the detail description of each protocol used in the study. References supporting the protocols are also included throughout.	

Experimental study design (statistics details)	Yes (indicate where provided: page no/section/legend)	n/a
State whether and how the following have been done**, or** if they were not carried out.		
Sample size determination	NA	
Randomisation		X
Blinding		X
Inclusion/exclusion criteria	Supplementary materials and methods (pages 2-4, 6, 13).	

Sample definition and in-laboratory replication	Yes (indicate where provided: page no/section/legend)	n/a
State number of times the experiment was replicated in laboratory	In the Supplementary Materials and Methods section, we describe the replicates for each *in vitro* experiment and the numbers of samples used in each experiment pg. 9-12.	
Define whether data describe technical or biological replicates	The number of technical or biological replicates included in each experiment is described in each figure legend:<br>Main manuscript, pages 18-19.	

Ethics	Yes (indicate where provided: page no/section/legend)	n/a
Studies involving human participants: State details of authority granting ethics approval (IRB or equivalent committee(s), provide reference number for approval.	Supplementary materials, Ethics statement: Humans, pg. 2:	
Studies involving experimental animals: State details of authority granting ethics approval (IRB or equivalent committee(s), provide reference number for approval.	Supplementary materials, Ethics statement: Dogs, pg. 2	
Studies involving specimen and field samples: State if relevant permits obtained, provide details of authority approving study; if none were required, explain why.	Supplementary materials, Ethics statement: Humans, pg. 2.<br>Supplementary materials, Ethics statement: Dogs, pg. 2	

Dual Use Research of Concern (DURC)	Yes (indicate where provided: page no/section/legend)	n/a
If study is subject to dual use research of concern, state the authority granting approval and reference number for the regulatory approval		X

Analysis

Attrition	Yes (indicate where provided: page no/section/legend)	n/a
State if sample or data point from the analysis is excluded, and whether the criteria for exclusion were determined and specified in advance.	No, only data points were removed according to the quality control exclusion/inclusion stages as detailed above.	X

Statistics	Yes (indicate where provided: page no/section/legend)	n/a
Describe statistical tests used and justify choice of tests.	Detailed statistical methods are outlined in the Supplementary Materials and Methods section.<br>An overall summary is provided in the 'visualization and statistics' section (pages 17-18).<br>Canine GWAS, pg. 6-7.<br>G2G pipeline, pg. 12-13. Further detail is also provided in the original publication (already cited, Kentistou et al, 2024).<br>Rare variant exome analysis statistical parameters, explained in supplementary materials and methods, pg. 13-14. Further detail is also provided in the original publication (Gardner et al, 2022).<br>Rare variant analysis for severe obesity cases in humans (SOPP/SCOOP), including purifying selection, supplementary materials and methods pg.14-15.<br>Quantitative PCR results were compared using an unpaired students t-test; Supplementary materials, pg. 11.<br>Mean dose response curve pEC50, supplementary materials, pg. 11.<br>Receptor surface expression and internalization, Supplementary materials, pg. 12.	

Data Availability	Yes (indicate where provided: page no/section/legend)	n/a
State whether newly created datasets are available, including protocols for access or restriction on access.	Data for newly created canine datasets (both phenotypic and genetic) are available on the Dryad platform.<br><br>Data for new canine WGS data used in the imputation panel are provided via ENA accession codes (https://www.ebi.ac.uk/ena/) and detailed on pg. 4 of the Supplementary materials and methods	
If data are publicly available, provide accession number in repository or DOI or URL.	Dryad DOI for newly created datasets: DOI: 10.5061/dryad.0vt4b8h85	

If publicly available data are reused, provide accession number in repository or DOI or URL, where possible.	Data obtained from the Golden Retriever Lifetime Study (GRLS) can be accessed via their data Commons website: https://datacommons.morrisanimalfoundation.org/.<br>Data for existing canine WGS data used in the imputation panel are provided via BioProject accession codes on pg. 4 of the Supplementary Materials and Methods<br>Several of the human data are reused from existing studies, all of which are referenced throughout the main and supplementary text files. Data for human analyses (G2G, UKBB, rare exome association, UKB, SCOOP, gnomAD) are publicly available to download directly or through creation of an account. Data can be accessed through the original study sources listed below.<br><br>Human WHR and BMI GWAS data GIANT consortium: https://portals.broadinstitute.org/collaboration/giant/index.php?title=GIANT_consortium_data_files&oldid=1066<br>Human HDL and triglyceride GWAS via the Global Lipids Genetics Consortium (GLGC) - https://www.lipidgenetics.org/#data-downloads-title<br>Human UKBB data: https://ams.ukbiobank.ac.uk/ams/<br>Human SCOOP case data: European Genome-Phenome Archive (EGA; https://ega-archive.org; Study ID: EGAS00001000124; Dataset ID: EGAD00001000432)<br>gnomAD webportal dataset: https://gnomad.broadinstitute.org/downloads<br>Mouse HypoMap data: https://www.nature.com/articles/s42255-022-00657-y	

Code Availability	Yes (indicate where provided: page no/section/legend)	n/a
For all newly generated code and software essential for replicating the main findings of the study:		
State whether the code or software is available.	Code for canine genetic imputation panel is publicly available via github. All other computational methods utilize simple fitting routines or common algorithms and have been described succinctly in plain English in the Supplementary Materials and Methods and/or in the original methodological manuscript(s). These detail methods are essential for replication of the main findings of this study.	
If code is publicly available, provide accession number in repository, or DOI or URL.	URL for imputation methodology code on github: https://github.com/GOdogs-Project/Imputation/releases/tag/version-0.1	

Reporting

Adherence to community standards	Yes (indicate where provided: page no/section/legend)	n/a
MDAR framework recommends adoption of discipline-specific guidelines, established and endorsed through community initiatives. Journals have their own policy about requiring specific		
guidelines and recommendations to complement MDAR.		
State if relevant guidelines (eg., ICMJE, MIBBI, ARRIVE) have been followed, and whether a checklist (eg., CONSORT, PRISMA, ARRIVE) is provided with the manuscript.		X
