# Supplementary material for: Canine genome-wide association study identifies DENND1B as an obesity gene in dogs and humans
Source: Science. Author manuscript; Available in PMC 2026 Feb 2. (PMC7618706; doi:10.1126/science.ads2145)
Supplement: Supplementary Materials [file EMS211953-supplement-Supplementary_Materials.zip › science.ads2145_sm.pdf]

Science
AAAS

Supplementary Materials for

Canine genome-wide association study identifies *DENND1B* as an obesity gene in dogs and humans

Natalie J. Wallis *et al*.

Corresponding author: Eleanor Raffan, er311@cam.ac.uk

Science **387**, eads2145 (2025)
DOI: 10.1126/science.ads2145

The PDF file includes:

Materials and Methods
Supplementary Text
Figs. S1 to S11
References

Other Supplementary Material for this manuscript includes the following:

Tables S1 to S15
MDAR Reproducibility Checklist

Materials and Methods

Ethics statement: Dogs

The research was approved by the Ethical Review Committee of the Department of Veterinary Medicine, University of Cambridge (CR73 and CR125), with sample collection at other centers also approved by local ethical review committees: Animal Health Trust Research Ethics Committee, and the University of Liverpool Research Ethics Committee (RETH000353). All dog owners gave full written consent to participate in the research.

Ethics statement: Humans

Data from the UK Biobank were accessed under application 9905. The Genetics of Obesity Study (GOOS) work was approved by the Multi-Regional Ethics Committee and the Cambridge Local Research Ethics Committee (MREC 97/21 and REC number 03/103). Each subject (or their parent for those under 16 years) provided written informed consent; minors provided oral consent. An anonymized human hypothalamic tissue sample was provided by the Cambridge Brain Bank for RNAscope analysis. Subjects were approached in life for written consent for brain banking, and all tissue donations were collected and stored following legal and ethical guidelines (NHS reference number 20/EE/0283). All studies were conducted in accordance with the Declaration of Helsinki.

Labrador retriever data collection

Labrador retriever owners were recruited by disseminating invitations to participate through veterinary practices, social media platforms, and emails to owners of relevant breeds via the Kennel Club, between 2013 - 2020. Additional dogs were pets which attended a specialist weight management clinic at the University of Liverpool School of Veterinary Science (n = 18). A population of Labrador retriever assistance dogs were also obtained through collaboration with Guide Dogs UK. Some recruitment targeted owners to volunteer if they had healthy weight or particularly overweight dogs making it unsuitable for estimating obesity prevalence. Demographic data were collected including breed, sex, neuter status, age, and coat color. Full clinical veterinary histories were also obtained and assessed by a trained veterinary professional.

Labrador retrievers were included if they met previously described criteria (*28*), outlined briefly here. Dogs were included if they had complete information on sex, neuter status, age and BCS. Clinically trained researchers reviewed their medical histories and excluded dogs affected by systemic disease, chronic orthopedic disease or on long-term medications known to cause weight gain or loss, such as corticosteroids and anti-seizure medication. Only Labrador retrievers over one year of age (at time of sampling) were included to avoid confounding by early life fluctuations in fat mass. Dogs with BCS <3 were also removed since this level of underweight may indicate poor underlying health. The final study population comprised 521 pet and 70 assistance Labrador retrievers.

Obesity was measured using the Body Condition Score (BCS), a validated measure of canine adiposity, measured using a 9-point BCS (*25–27*). Dogs are assigned to one of the body condition score categories according to how they match a set of descriptors and images and using a combination of visual and haptic assessment. The BCS chart used is shown in Fig. S1. Body weight was measured and BCS assigned by veterinary professionals who were provided with specific instructions about how to perform the assessment.

We used the Dog Obesity Risk and Appetite questionnaire to measure dogs' food motivation and owner control of food and exercise in the home environment. This validated, owner reported measure (*24*) contains 31 items which are used to obtain an 'owner control score' (combining factor scores on 'owner intervention', 'restriction of human food' and 'exercise') and food motivation score (combining factor scores on 'Interest in food', 'lack of fussiness' and 'Responsiveness and satiety'). Each statement is on a Likert scale scored numerically as 1-4 ('Not at all true' = 1, 'Somewhat true' = 2, 'Mainly true' = 3, 'Definitely true' = 4) or 1-5 ('Never' = 1, 'Rarely' = 2, 'Sometimes' = 3, 'Often' = 4, 'Always' = 5). Before factor calculation, some statements' scores are reversed. For example, for a statement like 'my dog would eat anything', stronger agreement indicates a high food motivation score but for the statement 'my dog takes his/her time to eat a meal', stronger agreement indicates lower food-motivation and so the scores are reversed. The mean response to items within each category is calculated and results reported as a percentage of the total maximum score. See Table S1 for details of DORA Questionnaire scoring items contributing to owner control and food motivation scores.

<u>DNA sampling</u>

The majority of the canine DNA samples were extracted from saliva collected using Performagene (PG-100) oral sponge swab kits (DNA Genotek). DNA was isolated according to the manufacturer's instructions using the Performagene PG-AC purification protocol (DNA Genotek). In dogs seen for clinical treatment, residual EDTA blood samples were stored at -20°C until shipping for analysis, and extracted using the DNeasy Blood & Tissue Kit (Qiagen, UK, Cat. No. 69504) according to the manufacturer's instructions.

<u>Genotyping and imputation overview</u>

Genome-level imputed data aligned to the CanFam3.1 reference assembly (GCA_000002285.2) were generated for the Labrador retrievers through one of two routes. The primary Labrador genetic dataset used for GWAS was generated via Route 1 (array genotyping followed by imputation) and the secondary or 'test' dataset used for polygenic risk score replication used a combination of Route 1 and Route 2 (low pass sequencing and imputation).

<u>Array genotyping with imputation (route 1)</u>

For 391 Labrador retriever participants DNA samples were genotyped using the 220k Canine HD BeadChip single nucleotide polymorphisms (SNPs) array (Illumina, San Diego, CA, USA). Post-QC genotypic data were imputed to genome level using an in-house pipeline which was made up of three wrapper scripts: generating an imputation reference panel, preparing the genotypic data (to be used for the GWAS) and the imputation process itself. The GitHub pipeline can be accessed via Zenodo (*74*). It was developed to run on the University of Cambridge High Performance Computing (HPC) system, with a SLURM workload manager so is not directly transferable but all the programs used within the pipeline are freely available to download and run.

Quality control for array genotypes was implemented using PLINK v.1.9. (*61*). SNPs residing on X and Y chromosomes were removed. Subsequent filters excluded markers with >3% genotype calls missing (--geno 0.03), individuals with >10% genotype calls missing (--

mind 0.1) and markers with minor allele frequency (MAF) <1% in the population (--maf 0.01). Ambiguous SNPs (A/T or C/G) were removed to limit strand and minor-allele confusion. Variants with extreme deviation from Hardy Weinberg equilibrium (HWE) were also removed (--hwe 0.00005). This data were also phased using SHAPEIT v2.r904 (*75*) using eight threads (-T 8) a window size of 2MB (--window 2) and effective population size of 200 (--effective-size 200).

To create a canine imputation reference panel, publicly available datasets were supplemented with additional genome sequence from Labrador retrievers generated locally all of which were aligned to CanFam3.1 genome build. Variant Call Format (VCF) files from a multibreed dataset were used to form most of the panel as described previously (*76*). The final imputation panel represented 676 individuals of 91 breeds, including 31 Labrador retrievers (BioProject accession PRJNA648123 and PRJNA726547). The panel was enriched with whole genome sequence from 7 Labradors extracted from the European Nucleotide Archive (ENA https://www.ebi.ac.uk/ena/ accession codes SRR7120183, SRR13340562, SRR13340566, SRR13340565, SRR13340564, SRR13340563, SRR13340570) and 5 from in-house WGS data (BioSample IDs SAMEA115942716, SAMEA115942718, SAMEA115942720, SAMEA115942723, SAMEA115942717, SAMEA115942719, SAMEA115942721, SAMEA115942722).

The reference panel was built using a combination of BEDtools v.2.20.1 (*77*), BCFtools v.1.9 (*78*), and PLINK v.1.9 (*61*). VCF files were filtered to include only variants with quality > 20 (QUAL > 20, equating to 99% probability) and duplicate SNPs were removed. All datasets were merged and matched for REF/ALT alleles; ambiguous SNPs (A/T, G/C) were removed from the panel. Variants which had missing data in one or two of the datasets were called as reference using the BCFtools v.1.9 (*78*) commands --force-samples --missing-to-ref. Only SNP variants with a read depth of >10 were used in the panel; other variant types were removed to improve imputation efficiency. The merged panel underwent PLINK QC excluding markers with >3% genotype calls missing (--geno 0.03), individuals with >10% genotype calls missing (--mind 0.1) and markers with minor allele frequency (MAF) <1% in the population (--maf 0.01). Prior to imputation, the panel was phased using SHAPEIT v2.r904 (*75*) using 20 threads (-T 20), a window size of 2MB (--window 2) and effective population size of 200 (--effective-size 200).

We used package IMPUTE2 v2 (*79*) for imputation of the genotypic data described above. A genomic interval was assigned from length 1 to max length of chromosome using the -int command. Effective population size was set at 200, using command -Ne 200. Analysis of regions over 7Mb was permitted using the -allow_large_regions command.

Following imputation, data were converted from dosage genotypes (probabilistic) to discrete allele calls (0/1/2) for each SNP. Although this conversion may lead to loss of information for more rare variants, it improves the power for the GWAS and allows for easier interpretation of data downstream. As IMPUTE2 generates an imputation certainty per SNP, we were able to filter the SNPs for those with a high imputation certainty of ≥ 0.7 (where certainty typically ranges from 0-1). We converted the data to PLINK format and performed QC excluding markers with >5% genotype calls missing (--geno 0.05), individuals with >5%

genotype calls missing (--mind 0.05) and markers with minor allele frequency (MAF) <5% in the population (--maf 0.05).

SkimSEEK™ genotyping (route 2)

Low-pass sequencing with imputation was performed using skimSEEK™ technology (Canine skimSEEK v2.0, Neogen Europe Ltd). The imputation panel consisted of the same 676 dogs representing 91 breeds and 53 million variants that formed the majority of our in house imputation panel. Imputation was based on an underlying statistical model described previously on the basis of the 'effective coverage' model, described in the first report of this method (*80*) and validated elsewhere (*81*). Variants of interest for further study could then be extracted from this dataset as appropriate. This method was used for 150 Labrador retriever dogs included in the secondary or 'test' data set.

Genotyping of the POMC deletion

Dogs were genotyped for a POMC variant of interest - frameshift mutation (17:19431807-19431821:GCGCCGGCCCGGGA>-, p.P187fs) (*19*) using custom TaqMan™ assays (ThermoFisher Scientific, UK). Custom-designed primers and probes were generated (Forward primer AGGCCTTCCCCGTCGAGTTC; Reverse primer TACTCCAGGTCGGCCAGCG; Wild-type probe AGGGCCCGGCCGCG with VIC fluorophore and MGB quencher; Deletion probe TCGGCCCCGGGCGT with FAM fluorophore and MGB quencher). For the genotyping reaction, the TaqMan gene expression master mix (ThermoFisher CAT#4369016, UK) was used, with the addition of 3% DMSO (ThermoFisher CAT #D12345). Primers were used at a concentration of 0.4µM, probes at 0.1µM, and genomic DNA at 0.4ng/µl. The thermocycling process was performed on an Applied Biosystems (Cat#4329001) 7900HT Fast Real-Time PCR System, following this profile: an initial step of 2 minutes at 50°C, followed by 10 minutes at 95°C, and then 40 cycles consisting of 15 seconds at 95°C and 1 minute and 30 seconds at 65°C. For analysis purposes, a cycle threshold (Ct) of 0.04 was set for the wild-type probe, and a Ct of 0.09 was set for the deletion probe, to account for the different lengths of the DNA fragments being amplified. If the difference between the Ct values obtained from the two probes was greater than 0.5, the genotype was confirmed through agarose gel separation.

Data from other dog breeds

Golden retriever data were obtained from the Morris Animal Foundation (Denver, Colorado, USA) having been generated as part of the Golden Retriever Lifetime Study (GRLS) (*82*). Golden retrievers were genotyped on the 1.1 million SNP Axiom Canine Genotyping Array Set A (ThermoFisher Scientific, Massachusetts, US). A population of pugs and flat-coated retrievers were recruited and phenotyped as described for the Labrador retrievers. Pugs were genotyped for >170k markers using the Canine HD BeadChip SNP array (Illumina, San Diego, CA, USA) and imputed in-house using the methods described for Labradors above with the modification that the imputation panel was enriched with pug whole genomes instead of Labradors.

Flat-coated retrievers were genotyped for specific variants of interest using Matrix-associated laser desorption/ionization time-of-flight mass spectrometry (MALDI-TOF MS) assay. Assay development and genotyping was performed at Neogen Genomics (Lincoln,

Nebraska, USA), employing the MassARRAY platform along with iPLEX GOLD chemistry in accordance with the manufacturer's protocol from Agena Bioscience (San Diego, California, USA). To design multiplex assays, the Agena Design Suite software provided by the manufacturer was used. A total of 24 markers of interest were split into two separate 'plex' pools of 12 and 11 markers each. The two multiplex assays were run on customer supplied genomic DNA, data generated, and quality check metrics applied, following which the final genotype calls were obtained.

Demographic data (sex, age, neuter status, breed, et cetera) and obesity-related phenotypic information were gathered for these additional populations. Obesity-related phenotypes for all datasets were measured by veterinary professionals or trained academic researchers. Phenotypic data underwent QC steps similar to that described above for Labradors (*28*). Dogs were removed if their age was <1 or >14 years old and if their BCS ≤3. Only individuals with complete information on relevant demographic data and BCS were included.

Genome wide association study (GWAS)

For the primary GWAS for BCS, inclusion criteria were more rigorous to ensure only dogs with the most robust phenotype data were included. Labradors were included only if they were 1-10 years old (to eliminate old age as a confounding variable); pets (due to difference in assistance dog environment and genetic divergence); had comprehensive medical histories; and their owners had not answered positively the question 'My dog regularly sees the vet for health problems (not including check-ups/vaccinations)' in the DORA questionnaire. Additionally, only dogs who underwent direct genotyping followed by in-house imputation were included to avoid batch effects on genotypic data. The final GWAS cohort comprised 241 Labradors. The remaining pet and assistance Labradors were used as a replication cohort for polygenic risk score development.

Dogs were assessed for pairwise relatedness using Identity by Descent (IBD) pi-hat estimates generated by PLINK v.1.9 (*61*) --genome command. Where estimated pi-hat ≥ 0.5 indicated 1st degree relative (parent/sibling), one of such a pair of dogs was removed. Using Genome-wide Efficient Mixed Model Analysis (GEMMA) software v0.98.1 (*83*), we then generated a relatedness matrix which was transformed to a distance matrix using R v.4.2.2 (*62*). Multidimensional scaling (MDS) was used to visualize any clustering of individuals. If distinct clustering was observed, indicating population stratification, only one cluster was taken forward.

Regression modelling was used to determine population-specific covariates relevant to BCS in the GWAS dogs. Using Akaike's Information Criterion (AIC), a stepwise model selection method, we performed minimal model selection for BCS. In the initial model, we included relevant risk factors based on prior knowledge, along with interactions. These risk factors comprised: sex, neuter status, sex:neuter status interaction term, age:sex interaction term, and age:neuter status interaction term. After implementing AIC, the minimal linear model for BCS was: BCS ~ sex*neuter status. Three covariates were therefore used for subsequent analyses in this cohort: sex, neuter status and sex:neuter status interaction term.

Using Genome-wide Complex Trait Analysis (GCTA) v.1.93.2 (*60*), we performed genome-based restricted maximum likelihood ratio (GREML) analysis (GREML_LDMS),

stratified by linkage disequilibrium (LD) and minor allele frequency (MAF). This is a method to estimate heritability and generates a multi-genetic relatedness matrix (multi-GRM) for imputed data or whole genome sequenced (WGS) data. This method is more suitable for imputed data compared with a standard GRM, as it corrects for the LD bias in the estimated SNP-based heritability (*63*). First, segment-based LD score was calculated using an LD-score region (--ld-score-region) of 200 kilobases (kb) and overlap of 100kb between adjacent segments. Second, individual SNPs were stratified by segment-based LD scores in R v.4.2.2 (*62*). Third, multiple GRMs were generated on the stratified SNPs using GCTA v.1.93.2 (*60*) command --make-grm. These GRMs, along with the covariates, were used via --mgrm and --covar to perform restricted maximum likelihood (REML) analysis with --reml to get an estimate for heritability.

The GWAS analysis used a mixed linear model association (MLMA) analysis from GCTA v.1.93.2 (*60*), implementing the –-mlma-loco option. This MLMA analysis excludes the chromosome on which the individual SNP is located when calculating the GRM. The multi-GRM generated during the GREML_LDMS analysis was used as a random effect in the GWAS model using the –mgrm command, as were the identified covariates using the –covar option.

For the threshold(s) of genome-wide significance we used a stringent Bonferroni corrected threshold of $p = 8.31x10^{-7}$. Bonferroni correction was performed on LD-pruned data using 1000bp window size, 2 bp step size and variance inflation factor (VIF) of 3.33˙. VIF threshold was based upon calculation: $1/(1-r^2)$ where $r^2 = 0.7$. Pruning was implemented using PLINK v.1.9 (*61*) using command --indep 1000 2 3.33˙. This Bonferroni-corrected genome-wide significance threshold is notably conservative since ‘independent’ SNPs were defined using a relatively high LD cut-off ($r^2 < 0.7$). A suggestive threshold was defined at $p = 1.54x10^{-4.81}$ where SNP associations deviated from the 95% confidence interval (CI) on the quantile-quantile (QQ) plot, an approach previously applied in canine GWAS studies (*29*, *30*).

For the lead SNP at each locus, the allele with a positive β effect on BCS outcome was identified as the ‘risk’ allele and the allele associated with a negative β effect on BCS was identified as the ‘non-risk’ allele.

Stepwise conditional analysis was performed on the GWAS output on a per-chromosome basis, to identify independently associated loci. The genotype of the most highly associated SNP was extracted and the GWAS MLMA rerun using the genotype as a covariate. This process was repeated on a per-chromosome basis until no more variants on a chromosome were significant. SNPs which became significant during the conditional analysis process but did not pass the original threshold were not taken forward as candidate loci. Additionally, SNPs which lost their association when conducting conditional analysis on other chromosomes were regarded as confounded and removed.

SNPs which were corroborated by multiple other proximal SNPs with above average significance were prioritized in further analysis. This was observed by a regional clustering of associated SNPs on the resulting GWAS Manhattan plots, indicating multiple variants in high LD and similarly associated with the trait. SNPs which passed the significance threshold but were not accompanied by such clustering were not taken forward as solitary SNPs may be indicative of a genotyping artefact.

To ensure that the imputation process and conversion to discrete genotype calls was not generating artefactual loci, the GWAS was rerun, first using only directly genotyped SNPs and, second, using the imputed 'probabilistic' genotypes (prior to converting to 0/1/2 discrete calls). Only loci which demonstrated significant association across all three GWAS were taken forward.

Identifying genes and variants at associated loci

Regions of interests were first defined based on pairwise r^2 measure of linkage disequilibrium with the lead SNP ($r^2 \geq 0.8$). Using these inclusive regions, gene lists were extracted from Ensembl Biomart (Archive release 104, May 2021) (*84*, *85*) to extract regional candidate genes from the CanFam3.1 genome. Due to incomplete annotation of the canine genome, syntenic genetic regions were identified using the UCSC liftOver tool (*86*) in the human genome (GRCh38 - hg38) and any additional annotated human protein coding genes in the syntenic region were added to the candidate gene list. Additionally, regions were checked in alternative genome builds CanFam4 (GCA_000002285.3) and CFam_1.0 (GCA_014441545.1) to ensure annotated genes were consistent.

Since our GWAS used only common SNP and no indels or structural variants, we interrogated WGS data from 25 Labrador retrievers using the Integrative Genomic Viewer (IGV) tool (*87*) to identify additional candidate variants within the initial regions of interest. Dogs were categorized as 'risk' or 'control' based on whether they carry the GWAS SNP of interest in each region. WGS data were then compared between the two groups to identify additional candidate variants (including indels) which co-segregated at a confidence of ≥70% between the groups. For this, confidence was calculated as a percentage of the dog's homozygous risk for the tagging SNP that were also homozygous for the variant in question, or from the heterozygous risk dogs if homozygous risk dogs were not available. All candidate variants were annotated using Ensembl's Variant Effect Predictor (VEP) v.109.2 (*88*). Since there is limited annotation on the CanFam3.1 genome, we tested whether human genetic positions syntenic to canine variants were annotated as regulatory elements or epigenetic hotspots.

Fine mapping

The regions defined by being in high LD with the lead variant were further refined by performing haplotype mapping and variant phasing. Where possible, haplotype mapping using PLINK v.1.9 (*60*) command –blocks identified the haplotype in which the top SNP resided using default parameters (using the method of Gabriel et al. 2002 (*89*) of defining haplotype blocks, using 95% confidence bounds on the D prime (D') measure of allelic association to estimate recombination between SNPs). Using the bounds of the inferred haplotype, regions of interest were refined. Variants 100% 'in-phase' with the top SNP were also identified and were encompassed into the refined region of interest in cases where they fell out of these haplotype regions. Protein coding genes within those refined regions were identified and further investigated. If refined regions did not harbor (or were not proximal to) a protein-coding gene, they were not explored further.

Investigation of candidate genes

Candidate genes were input into STRING v.11.5 (*82*)

and PANTHER v.17.0 databases (*91*). The STRING database was used to investigate protein-protein interactions in the candidate genes, encompassing both direct (physical) and indirect (functional) associations with a confidence threshold of 0.4 ('medium') as the cut off. The PANTHER database was used for the identification of pathways with functional links to the candidate gene list, testing in mouse, dog and human datasets using Fishers exact test with Bonferroni correction for multiple testing.

Publicly available cross-species databases were utilized to investigate candidate genes and to hypothesize mechanisms of action for the variants/genes of interest. Mouse knockout and targeted mutation models were investigated through the International Mouse Phenotyping Consortium (IMPC) (*92*) and the Mouse Genome informatics Database (MGD) from the Jackson laboratory (*93*). Summary statistics for previous human GWAS and phenome-wide association studies (PheWAS) were also explored to identify any existing association(s) with obesity and/or related traits. NHGRI-EBI GWAS catalog (*94*) was interrogated and if variants in candidate genes were identified as associated with traits of interest then they were explored further. GWAS Atlas (*95*) phenome wide association analysis (PheWAS) results uses GWAS catalog statistics to analyze several phenotypes against a single gene/variant. The *p*-values for PheWAS associations with phenotypes of interest were compared to a Bonferroni-corrected significance threshold. This threshold was calculated using the PheWAS significance threshold of 0.05 and correcting for the number of phenotypes associated with each gene. Genebass v. 0.13.0 (*96*) is a resource of exome-based association statistics from UK Biobank (UKB) in which we explored whether there were any associations with endocrine/metabolic traits, or physical traits of interest. The Online Mendelian Inheritance in Man (OMIM) catalogue was explored to check the clinical synopsis for candidate genes. More generally, we performed wide-scale literature searching for protein function and candidate gene empirical study.

To investigate if these genes have previously been implicated in other complex disorders, we obtained gene-disease association data from the Developmental Disorders Genotype-to-Phenotype (DDG2P) database (*97*), a curated resource linking genetic variants with developmental disorders based on evidence from clinical and genetic studies (downloaded from https://www.deciphergenomics.org/ddd/ddgenes on 28/10/2024).

<u>Expression analysis for genes of interest</u>

We evaluated tissue-specific expression of candidate genes by employing a multi-species approach to publicly available RNA sequencing data. We tested whether candidate genes were enriched in relevant tissues (brain, adipose tissue) or ubiquitously expressed across tissue types using the Human Protein Atlas (HPA) database v23.0 (*98*) and the Genotype-Tissue Expression (GTEx) portal v.8 (*99*) for human data and the MGI - Gene Expression Database (MGI-GXD) from the Jackson laboratory (*100*) for murine expression.

Canine bulk RNA sequencing data obtained from the BarkBase data repository (*41*) were used to examine expression across tissues, with data from 5 individuals analyzed across all available tissues, of which hypothalamus was not included (adipose, adrenal gland, brain cortex and cerebellum, kidney, liver, pancreas, pituitary gland, skin, stomach, thyroid gland). Candidate genes of interest were extracted from the BarkBase files and mean expression (in FPKM -

Fragments per kilobase of transcript per million fragments mapped) calculated on a per tissue basis, combining all donors.

To understand gene expression with a higher degree of resolution we interrogated single cell/single nucleus RNA sequencing (scRNAseq/snRNAseq) from the mouse and human brain, focusing on the hypothalamus because of its well characterized role in energy homeostasis. The scRNAseq murine HypoMap (*42*) represents eighteen datasets encompassing ~385,000 cells from 29 samples from the mouse hypothalamus. To look at hypothalamic data from the human, we utilised the snRNAseq dataset from human HYPOMAP (*43*). In both datasets, candidate genes were explored for brain region expression specificity (particularly nuclei implicated in energy homeostasis), co-expression with known obesity genes (specifically, those in the leptin-melanocortin pathway) in R v.4.2.2 (*62*) and visualized using Seurat v.4.0.2 (*101*). Co-expression of receptors with *Dennd1b/DENND1B* was visualized by taking a subset of neurons expressing >1 *Dennd1b/DENND1B* counts.

<u>RNAscope</u>

Simultaneous detection of Human *MC4R* and *DENND1B* mRNA was performed on formalin fixed paraffin embedded hypothalamic sections using Advanced Cell Diagnostics (ACD) RNAscope® 2.5 LS Duplex Reagent Kit, and RNAscope® probes 1239438-C1 and 538798-C2 (ACD, Hayward, CA, USA). The assay was performed as previously described (*70*) with one modification, that the time in Amp5 time was extended from 15 to 20 minutes. Slides were imaged on a Slide Scanner Axio Scan.Z1 microscope (Zeiss). Images were taken in regions where positive cells were detected using a 40x air objective and sharpened using the Unsharp Masking processing in ZEN Blue (Zeiss). CZI files were read into QuPath v0.5.1 for analysis. MC4R and DENND1B positive cells were detected by manual inspection and color deconvolution.

<u>Cell culture and transfection</u>

Adherent HEK293 (AdHEK) cells were obtained and cultured as previously described (*71*). AdHEK were maintained in DMEM-Glutamax media (Sigma-Aldrich) with 10% fetal bovine serum (FBS, Sigma) at 37ºC, 5% CO_2. Full-length *DENND1B* cDNA was PCR amplified (Reagents from New England Biolabs) from human cDNA and inserted into a pcDNA3.1 expression construct by restriction enzyme cloning. Human SNAP-GHSR was purchased from Cisbio (UK). For the human SNAP-MC4R construct, MC4R was PCR amplified from a template of Tango-MC4R (Addgene #66430, from Bryan Roth (*102*)) using reagents from Promega and sequence verified by Source Bioscience. Single nucleotide polymorphisms were introduced by construct high-fidelity PCR amplification using primers containing the polymorphism at the center. Cycle conditions were: 95ºC for 30 seconds, followed by 12 cycles of 95ºC for 30 seconds, 55ºC for 1 minute, and 72ºC for 7.5 minutes, followed by hold at 4ºC. The original DNA in the PCR product was degraded by Dpnl digestion. Resulting expression constructs were sequence-verified (service provided by Department of Biochemistry, University of Cambridge). The pGloSensor-22F plasmid was purchased from Promega. Human DENND1B knockdown was achieved by transfection of 3 unique 27mer siRNA duplexes (SR316077, OriGene Technologies). All transfections were performed using Lipofectamine 2000 (LifeTechnologies) according to manufacturer’s instructions.

Gene expression analysis

Overexpression and knockdown of *DENND1B* was assessed using quantitative real-time PCR (qPCR). RNA was extracted using Tri-Reagent (Sigma-Aldrich) and purified with the QIAwave RNA mini kit (Qiagen). RNA was treated with the TURBO DNA-free kit (Thermo Fisher Scientific) and 1µg made into cDNA using the High-Capacity cDNA Reverse Transcription kit (Thermo). *DENND1B* and *GAPDH* specific primers were designed to achieve an amplicon with a melting temperature between 58°C-62°C, size of approximately 100bp and no secondary structure. Duplicate qPCR reactions of 2µl cDNA were performed with Fast SYBR Green Master Mix using pre-set quantitative PCR cycling methods on the 7500 Fast Real-Time PCR System (Thermo). *GAPDH* RNA levels were used to normalize gene expression using the $2^{-\Delta\Delta Ct}$ method (*103*) and visualized as a percentage of the un-transfected cells. Two technical replicates were combined, and an unpaired student's t-test used to determine significant differences in gene expression.

cAMP GloSensor Assays

AdHEK were seeded in six-well plates and 24-hours later transfected with 100 ng pGloSensor-22F and 1000 ng SNAP receptor (human MC4R). When overexpressing DENND1B, 1000 ng pcDNA3.1 (empty vector, DENND1B WT or DENND1B R501C) were co-transfected. For knockdown, 24-hours after DNA transfection, a combination of 3 unique siRNA (10 nM) were transfected. Forty-eight hours after DNA transfection, cells were re-plated in 96-well white plates (Nunc) in FluoroBrite DMEM media (ThermoScientific), supplemented with 10% FBS. After 6 hours, cells were incubated in 40 µL of equilibration media consisting of FluoroBrite DMEM media containing 10% FBS, 2% (v/v) dilution of the GloSensor cAMP Reagent stock solution according to manufacturer's instructions and 0.5 mM IBMX. Cells were incubated for 1 hour at 37°C before basal luminescence was read on a FLUOstar® Omega (BMGLabtech) or a Glomax (Promega, Madison, USA) plate reader for 6 minutes before addition of receptor agonist. For MC4R assays, α-MSH (Bachem AG) was added (100 µM – 10 nM). As a positive control, we used 10 µM forskolin (Sigma). For GHSR assays, MK-0677 (Tocris, Abingdon, UK), a high affinity ghrelin receptor agonist, was added (10µM - 10nM). Plates were read for a further 25 minutes. Statistics were performed as for the IP-1 experiments below.

IP-1 Gq assay

IP-1 assays were performed with the Cisbio IP-One Gq HTRF kit (Revvity, Codolet, France). Cells were co-transfected with 500 µg of SNAP-tagged GHSR and either 500 µg of pcDNA3.1, *DENND1B*-WT or *DENND1B*-R501C variant. Forty-eight hours after transfection, cells were washed once in 1X PBS and resuspended in FluoroBrite DMEM (Thermo Fisher Scientific) supplemented with 10% FBS and 100 mM lithium chloride at 500 µL/well. The cells were then replated into 384-well plates at 7 µL/ well. MK-0677 (Tocris) agonist dilutions were made in the stimulation buffer provided with the IP-One kit. MK-0677 was added to cells at 7 µL/well and incubated at 37°C for 1 hour. Cells were lysed according to the IP-One kit protocol. IP-1 signal was measured on a BMG Labtech PHERAstar microplate reader. The supplied IP-1 standard concentration range was run in tandem with each experimental repeat. Raw values were interpolated from the IP-1 standard curve. Non-linear log regression fit (dose-response – stimulation curve; three parameters logistic equation) was fitted to the datasets using GraphPad Prism 9. Independent experiments were converted into a combined concentration-response graph

by normalizing data to the fitted maximal response of GHSR co-transfected with pcDNA3.1. Mean pEC50 values from at least four independent experiments before normalization were presented as mean ± SEM. pEC50 values from independent experiments were grouped and compared by one-way ANOVA.

HILO imaging

AdHEK cells were seeded on 24mm coverslips (VWR) and transfected with 500ng of each plasmid 48 hours prior to experiments. Venus-Kras (obtained from Nevin Lambert, Augusta University) was co-transfected at 500ng per well as a marker of the cell plasma membrane. For knockdown, a combination of 3 unique siRNA (10 nM) were also transfected 24 hours prior to experiments. SNAP-Surface Alexa Fluor 647 (NEB) was diluted 1:1000 in FluoroBrite complete media and applied to cells for 20-minutes, before washing and imaging. Coverslips were mounted onto plastic imaging chambers with a rubber seal and filled with imaging medium (HBSS with 10mM HEPES, Sigma). HILO images were acquired on a custom-built TIRF microscope (Cairn Research) comprising an Eclipse Ti2 (Nikon) equipped with an EMCCD camera (iXon Ultra, Andor), a 488 nm diode laser, a hardware Perfect Focus System, a TIRF iLas2 module, and a 100× oil-immersion objective (NA 1.49, Nikon). The objective and samples were maintained at 37°C in a heated enclosure. Images were acquired on MetaMorph software (Molecular Devices) using a frame exposure of 50–200 ms with an image acquired before ligand stimulation and a subsequent image taken every 30s thereafter, up to 20 min. Images were analyzed using ImageJ. Images were stacked, regions of interest corresponding to cell outlines assigned for each cell, and intracellular fluorescence intensity quantified. Data were normalized to the cytoplasmic intensity at time 0. Colocalization between SNAP-labelled receptors and Venus-Kras was measured using the ImageJ plugin JACoP (*72*). Regions of interest were selected and cropped so that images contained single cells, then JACoP thresholds for each channel were set using the Costes' automatic thresholding (*72*). Receptor surface expression was compared using a one-way ANOVA and receptor internalization using a Mann-Whitney test.

Human comparative genomics

Canine regions of interest (ROI) bounded by the limits of SNP with $r^2 \geq 0.8$ with the lead SNP at each locus were mapped to orthologous regions in the human genome using the UCSC liftOver tool (*86*) and integrated with human genome-wide association study (GWAS) and exome-wide association study (ExWAS) data for BMI and also GWAS data of other metabolic traits.

Human GWAS gene prioritization

We interrogated human GWAS data on body mass index (BMI) and waist-hip ratio (WHR) adjusted for BMI in up to 806,834 and 694,649 individuals respectively from the GIANT study (*31*, *104*) and circulating high density lipoprotein (HDL) and triglyceride levels in up to 1,253,277 European individuals from the Global Lipids Genetics Consortium (GLGC) study (*105*) (up to n = 1,253,277).

To identify independent GWAS signals and prioritize causal gene candidates at the resulting loci, we used the "GWAS to Genes" (G2G) pipeline as described by Kentistou et al. 2023 (*39*) and discussed in brief below. GWAS summary statistics were filtered to retain variants with a MAF>0.1%. Quasi-independent genome-wide significant (GWS, at a multiple test

corrected *p*-value threshold of $5x10^{-8}$) signals were initially selected in 1Mb windows and secondary signals within these loci were further selected via conditional analysis in GCTA v.1.93.2 (*60*), using an LD reference derived from the UKB study. Primary signals were then supplemented with unlinked ($r^2 < 5\%$) secondary signals, whose association statistics did not overtly change in the conditional models and signals were mapped to proximal NCBI RefSeq genes, within 500kb windows.

Independent signals and closely linked SNPs ($r^2 > 0.8$) within the associated loci were annotated if they were coding variants within the identified genes or if they mapped within known enhancers of the identified genes, via activity-by-contact (ABC) enhancer maps (*38*). Signals were also annotated with their physically closest gene. Gene-level associations were then determined via Multi-marker Analysis of GenoMic Annotation (MAGMA) (*106*) by collapsing all coding variants within a gene. Genes with FDR-corrected MAGMA $p < 0.05$ were considered associated with the GWAS outcomes. Colocalization analyses between the GWAS and expression- or protein- quantitative trait loci (eQTL or pQTL) data were also performed via SMR-HEIDI (summary data–based Mendelian randomization-heterogeneity in dependent instruments) method v0.68, (*107*) and the ABF function within the R package “coloc” v5.1.0 (*108*). For the former, we considered gene expression to be influenced by the same GWAS outcome variants if the FDR-corrected SMR test $p < 0.05$ and HEIDI test $p > 0.001$. For the latter, loci exhibiting an H4 PP > 0.75 were considered to show evidence of colocalization. For eQTL analyses, these were applied for specifically enriched tissues (via LDSC-SEG) (*109*), as well as cross-tissue meta-analyzed GTEx eQTL data v.7 (*110*) and data from blood eQTL (*111*) and Brain-eMeta (*112*) studies. Lastly, genes residing within each locus underwent prioritization using the gene-level polygenic priority score (PoPS) method (*113*). Causal candidate genes were then prioritized by overlaying all of the above information and scoring the strength of evidence observed. For further details about the specific application of this method, see Kentistou et al. 2023 (*39*).

BMI GWAS signals identified as outlined above, were mapped (if present) to the human genomic regions syntenic to the Labrador retriever GWAS ROIs. Orthologous genes mapping within each ROI were also queried for the strength of G2G evidence in the two anthropometric (*31*, *104*) and two lipid (*105*) GWAS described above.

Rare exome variant association in UK Biobank (UKBB)

We performed exome sequencing-based rare variant burden analyses, as described in Gardner et al. 2022 (*114*) and described in short below. We queried population-level VCF files data for 454,787 individuals from the UKBB study via the UKBB Research Access Platform (https://ukbiobank.dnanexus.com; application number 9905). BMI for all participants was obtained from the UKBB data showcase (field 21001).

We excluded individuals with excess heterozygosity or autosomal variant missingness ≥ 5%, based on the available genotyping array data, or those who were not included in the subset of phased samples as defined in Bycroft et al. 2018 (*115*). We also excluded participants who were not of broadly European genetic ancestry, leaving a total of 421,065 individuals (including related participants) for further analysis. Using BCFTools v.1.9 (*78*) multi-allelic variants were split and left-normalized, and all variants filtered using a missingness based approach. SNV

genotypes with depth < 7 and genotype quality < 20 or indel genotypes with a depth < 10 and genotype quality < 20 were set to missing (./.). We also tested for an expected reference and alternate allele balance of 50% for heterozygous SNVs using a binomial test; SNV genotypes with a binomial test p. value ≤ $1x10^{-3}$ were set to missing. Following genotype filtering, variants with > 50% missing genotypes were excluded from further analysis. Variants were then annotated with the Ensembl VEP v.109.2 (*88*) with the 'everything' flag and the LOFTEE plugin (*36*). For each variant we prioritized a single MANE v.0.97 or VEP canonical ENSEMBL transcript and most damaging consequence as defined by VEP defaults. To define Protein Truncating Variants (PTVs), we grouped high confidence (HC, as defined by LOFTEE) stop gained, splice donor/acceptor, and frameshift consequences. All variants were subsequently annotated using CADD v1.650 (*116*). After excluding individuals with missing data, 419,692 individuals with BMI measures remained for downstream analysis.

To assess the association between rare variant burden and BMI we implemented BOLT-LMM v2.3.551 (*64*), using a set of dummy genotypes representing the per-gene carrier status. For the latter, we collapsed variants with a minor allele frequency (MAF) < 0.1% across each gene and defined carriers of variants as those with a qualifying high confidence PTV (HC PTV) as defined by VEP and LOFTEE or "Damaging" variants (DMG), including missense variants with a CADD score ≥25 and the aforementioned HC PTVs. Genes with fewer than ten carriers were excluded. BOLT-LMM was run with default settings and the 'lmmInfOnly' flag. All analyses were controlled for sex, age, age-squared, WES batch, and the first ten genetic ancestry principal components as calculated in Bycroft et al., 2018 (*115*).

Gene-level BOLT association summary statistics were then extracted for genes falling within the identified ROIs. We identified 16 orthologous genes with at least 10 carriers of qualifying exome variants, setting the multiple-test corrected threshold at $p \leq 0.0031$ (0.05/16). Any downstream sensitivity analyses (such as leave-one-out tests) were performed in the same individuals as the BOLT associations, but using linear models in R.

Rare variant enrichment analysis in SCOOP

A case-versus-control, rare variant enrichment analysis was performed using independent case and control datasets. The Severe Childhood Onset Obesity Project (SCOOP) dataset is a subset of the Genetics of Obesity Study (GOOS) of UK British origin. This dataset was used as the case dataset and tested against the control dataset obtained from the Genome Aggregation Database (gnomAD) resource (*36*).

The UK10K SCOOP dataset (n = 982) was obtained from the European Genome-Phenome Archive (EGA; https://ega-archive.org; Study ID: EGAS00001000124; Dataset ID: EGAD00001000432, downloaded on 02/03/2021). Exome sequencing and variant calling for this dataset are described previously (*117*, *118*). Only variants that were both polymorphic in SCOOP and marked with a "PASS" flag from GATK (v.1.3-21) were retained. Coordinates were lifted over from build 37 to build 38 using the LiftoverVcf module from GATK.

GnomAD exome r2.1.1 variants (GRCh38 liftOver) were downloaded from the gnomAD webportal (*36*) (downloaded on 25/10/2022). Gene annotations were based on Ensembl Release 108 (downloaded on 25/10/2022). Again, we used BCFTools v.0.1.18-r579 (*78*) to subset the

variant data to the genes of interest. Only very rare variants were retained in gnomAD v2.1.1 non-Finnish Europeans. We used a minor allele count (MAC ≤ 3), equal to an allele frequency of ~ 0.0026%.

The syntenic ROI that were highlighted through the canine analysis were extracted from both case and control datasets using BCFTools v.0.1.18-r579 (*78*) Ensembl VEP, including the combined annotation dependent depletion (CADD) plugin, was used to annotate all variants. Genotype counts were extracted with PLINK v2.0 and a missingness filter < 0.1 at site-level, applied to both datasets.

The final data analysis steps were run using R 4.1.1. For this analysis, we only retained deleterious variants, defined as moderate/high impact predicted by VEP, and/or CADD > 25. We generated cumulative minor allele counts for each of the genes in the regions of interest. For each gene, we applied a one-sided Fisher's test to determine whether there was an excess burden of very rare deleterious variants in the gene of interest in the SCOOP cohort vs gnomAD v2.1.1 non-Finnish Europeans. Alpha significance level corrected for multiple testing was set at p = 0.01 (0.05/5) and a suggestive significance level at p = 0.05.

If suggestive or significant enrichment in the case cohort was identified in any of the genes, we explored the specific variants and carriers further. Familial genotypes and phenotypes were obtained as were clinical phenotypes of the probands. Where possible, we investigated if the genotypes of the variants of interest segregated with obesity status within affected families. Obesity was assessed using the age-adjusted BMI chart relevant to each individual.

Severe Obesity in Pakistani Population (SOPP) cohort

We also investigated genes of interest in the Severe Obesity in Pakistani Population (SOPP) cohort. The cohort is comprised of well-characterized individuals originating from a distinct inbred population who reside in relatively uniform environmental conditions. The selection criteria encompass a BMI or BMI SDS (standard deviation score/Z score compared to WHO global reference data (*65*, *66*) for age >35 or 3.5, respectively; the onset of obesity prior to 5 years of age, coupled with pronounced hyperphagia. Additionally, it is required that the participants exhibit either first- or second-degree parental consanguinity, with parents being either of normal weight or overweight, but explicitly excluding obesity. In all the cases, comprehensive anthropometric measurements are systematically conducted for all participants, alongside the detailed documentation of patient and/or family medical histories. We hypothesized that affected patients would be homozygous for deleterious variants in the genes of interest.

Ancestry diverse public datasets including gnomAD v.2.1.1, NCBI (*67*), TopMED (*68*), and NIH ClinVar (*69*) were used as comparator populations. To gain insight into mechanism of action for candidate variants (in addition to VEP annotation) they were examined using UniProt (*119*) and AlphaFold (*120*). UniProt allowed for identification of protein domains impacted by coding variants.

Testing for Purifying Selection

The two constraint metrics pLI (*121*) and LOUEF (*36*) were used to evaluate the intolerance of a gene to high confidence LoF mutations in the gnomAD database. We accessed the gnomAD v4.1 dataset (downloaded on 25/10/2024) and implemented thresholds suggested by the developers of each metric (<0.4 for LOUEF and >0.9 for pLI).

Canine polygenic scoring

Using the GWAS results, a polygenic score method was created using the 'clumping and thresholding' technique (*46*). First, an arbitrary *p* value significance threshold was allocated to the GWAS output ($p < 0.0004$) retaining only SNPs which passed this threshold (n = 481). Variants which had a pairwise r^2 measure of LD ≥ 0.7 were treated as the same locus, with the most significant 'lead' SNP per locus taken forward to the PRS calculation. Using this approach, 23 SNPs contributed to the primary PRS. Polygenic score was calculated as the sum of the product of each 'risk' allele genotype weighted by effect size from GWAS output.

PRS = (Risk allele count$_{SNP1}$ * β effect size$_{SNP1}$) + (risk allele count$_{SNP2}$ * β effect size$_{SNP2}$) + etc…

PRS was then refined in the secondary/test set of Labrador retrievers (n = 350). We retained only SNPs which retained a positive effect size for BCS outcome in the test set (significant association was not a requirement). Sixteen SNPs remained, contributing to the PRS method.

We subsequently tested the PRS method in multiple purebred datasets for prediction of obesity-related phenotypes. Genotypes for the sixteen contributing SNPs were extracted from the multiple datasets using PLINK v.1.9 (*61*) command –-extract. If genetic datasets were missing some variants, alternative variants were identified by finding a proximal SNP in high LD (r^2 > 70%) with the original, with a similar effect size and *p* value in the original GWAS output. If no suitable replacement SNP could be identified, the PRS was calculated with only the retained variants. Polygenic score per individual was calculated as described above.

Polygenic score predictive value

Importantly, prediction of obesity-related traits by the PRS was tested specifically for each purebred population, since specific breed groups act as closed populations. The Labrador retriever discovery (n = 241) and test (n = 350) populations were treated separately for this analysis. Minimal modelling was performed with known risk factors as described in the Genome Wide Association Study (GWAS) section above. After implementing AIC, the remaining covariates in the minimal model were specific to each population. The polygenic score was then added to the relevant model and tested for its predictive value using the linear model command lm() in R v.4.2.2 (*62*). To test whether individual variants were associated in the different breed groups, analysis was conducted in a similar manner.

For the GRLS, FCR and Pug populations, polygenic score was used to predict BCS and body weight (kg). For GRLS dogs the data were longitudinal so a single point in time was chosen for each participant. For the BCS phenotype, this entailed identifying the youngest age at which the dog had its highest BCS recorded. For body weight, the earliest weight was used. For the pug and FCR populations, phenotypic data were only recorded once.

Of the combined cohort of Labrador retrievers (n = 591), 298 had a food motivation phenotype. After minimal model selection for food motivation (as above), polygenic score was tested for prediction of the food motivation phenotype in these dogs. A linear model was used via the lm() command in R v.4.2.2 (*62*), accounting for relevant covariates. To calculate variance explained by PRS and individual PRS SNPs, the R^2 goodness-of-fit value from the linear regression model was used.

Polygenic score distribution in Labrador retrievers

To assess the distribution of polygenic score in Labrador retrievers and how it related to obesity status, polygenic score was binned into tertials to assign 'low', 'middle' and 'high' PRS groups. BCS was then categorized into three groups: healthy weight (BCS $\leq$ 5), overweight (BCS 6-7), very overweight/obese (BCS >7).

To test whether genetic factors underlie known risk factors for obesity in Labradors (chocolate coat color and being from the assistance dog group), polygenic score distribution was compared between different demographic groups in the combined set of Labradors (n = 591), incorporating covariates from minimal model selection in that group.

To test whether there was a significant difference in polygenic scores between the categorical coat color groups, ANOVA was performed (accounting for relevant covariates in the minimal model). Assumptions for a parametric ANOVA test were not met, but an alternative non-parametric test allowing for covariates is not available. Therefore, a non-parametric Kruskal-Wallis test was also performed to confirm the ANOVA association. For post-hoc, pairwise comparisons between coat color groups, non-parametric Wilcoxon rank sum tests were used. To assess if there was significant difference in polygenic score between assistance dogs and pet dogs, a Welch's t-test was performed. However, as above, not all assumptions for a parametric t-test were met so an additional analysis was performed using the non-parametric Wilcoxon rank sum test to confirm association.

High and low risk polygenic scores

Based on polygenic score distribution in the combined set of Labrador retrievers, polygenic score was re-grouped into quintiles. 'Low risk' and 'high risk' polygenic score group was then assigned based on lower two/upper two quintiles, respectively. Those individuals falling into the third/middle quintile were removed, with the aim of comparing only the ends of the polygenic risk distribution.

The interaction between owner management of the food and exercise environment on obesity outcome was also compared between high and low polygenic risk groups. Owner control was calculated using DORA questionnaire factor scores, as described above. Stepwise minimal model selection was implemented with inclusion of PRS and owner control score interaction. The owner control score was then tested for association in a linear model for BCS in the low-risk vs the high-risk polygenic score groups separately. Mean owner control was also compared between the two data subsets to ensure this was not confounding the findings.

Visualization and statistics

All data analysis and visualization were performed using R/R Studio v.4.2.2 (*62*). Packages tidyverse v.2.0.0 (*122*), data.table v.1.14.8, easystats v.0.7.0, ggplot2 v.3.4.4 were used for data analysis and visualization. Specifically, visualization of statistical assumptions was implemented using easystats. Decimal data are reported to two/three significant figures (sf) or two decimal places (dp) as appropriate.

For visualization of phenotype-genotype association and for polygenic score analysis across datasets, residual phenotypes were used. This is a method to better visualize cause and effect for a specific variable by adjusting the phenotype for covariate effects. The command lm() is used to perform a linear model and summary() used to determine the effect size (β) for all factors. The phenotype of interest (to be plotted) can then be adjusted for the relevant covariates per individual based on the equation: Residual phenotype = phenotype - (β effect covariate A * (covariate A - mean covariate A)) - (β covariate B * (covariate B – mean covariate B).

Supplementary Text

Lead SNP in canine GWAS and definition of genes of interest

Conditional analysis and haplotype mapping identified seven independent loci, of which five contained protein coding genes (Fig. 1B-K, Table S3). Specifically rs850596879 lies within an intron of *CSNK1A1*, and had MAF = 0.09, p = 4.73x10^{-6}, effect size β = 0.80 BCS; rs853159627 is upstream of *CDH8* and had MAF = 0.25, p = 7.18x10^{-6}, β = 0.52 BCS; rs8831037 is in an intron of *CARD11* and had MAF = 0.16, p = 1.43x10^{-5}, β = 0.66 BCS, with *CNA12*, *BRAT1* and *AMZ1* also present in the mapped region; rs24430444 is in an intron of *DENND1B* and had MAF = 0.06, p = 5.54 x 10^{-7}, β = 0.94 BCS with *LHX9* also present in the mapped region; rs22632455 is in an intron of *SEMA3D* and had MAF = 0.14, p = 1.41x10^{-6}, β = 0.96 BCS.

Human genetic associations with BMI for canine candidate genes

Human genomic regions orthologous to the fine mapped obesity associated canine loci were identified and interrogated for the presence of GWAS associations for BMI or related metabolic traits. Protein coding genes within those regions were considered as candidate genes and tested for their association with obesity in a GWAS for BMI on 806,834 participants from the GIANT study (*31*); an exome-wide association study (ExWAS) of rare (MAF < 0.1%) deleterious exome variants from 454,787 individuals from the UK Biobank study (UKB) (*32–34*); the Severe Childhood Onset Obesity Project (SCOOP-UK) (*35*) (n = 982) in which we tested for enrichment of very rare (MAF < 0.0026%), predicted deleterious (CADD ≥ 25) variants compared to reference exomes of similar ancestry (gnomAD v2.1.1, n = 56,885); and the Severe Obesity in Pakistani Population (SOPP) cohort of patients with severe, early onset obesity from a highly consanguineous population, all with unaffected parents and no prior genetic diagnosis, in which we focused on homozygous, predicted deleterious variants (*44*, *45*).

In the GIANT GWAS there were associations with human BMI for variants in *DENND1B* (further details in main text) and *CARD11* (rs7811825, MAF = 0.14, p = 9.02x10-9, β = -0.0146 kg/m^2, Table S7). In each case, the human effect sizes were small and the statistical association significant but modest, meaning these associations were likely to be overlooked as having potential to meaningfully influence BMI at a population level. *CARD11* encodes a signaling scaffold protein which is best understood for its role in the adaptive immune response (*123*) and it is not clear how this might link mechanistically to increased BMI.

Rare variant enrichment was evident in the SCOOP cohort for *CSNK1A1* (p = 0.006, Fig. 2A, C) due to the presence of two variants which were absent from gnomAD (Table S11), map to the protein kinase domain and have high CADD scores (>27). A heterozygous variant *CSNK1A1* p.V87G was present in a proband with a complex phenotype that included severe, early onset obesity, speech delay, constipation, sleep disturbance and behavioral problems. At presentation at 3 years 4 months old, her weight was 23.3 kg, BMI 22.13 kg/m^2, BMI standard deviation score (SDS) 3.33. The two other family members from which DNA was available had the reference allele and were normal weight (mother BMI 23.6 kg/m^2 and brother age 4 years 4 months BMI 15 kg/m^2, BMI SDS -0.58). A second heterozygous variant *CSNK1A1* p.G80D was identified in a patient with severe, early onset obesity who presented aged 12 years with weight 83.6 kg, BMI 48.13 kg/m^2, BMI SDS 4.28. Her heterozygous, mother was also obese (BMI 36.2 kg/m^2) and had type 2 diabetes. The father harbored only the reference allele and was borderline obese (BMI 31.2 kg/m^2). Further information is available in Table S14. *CSNK1A1* is involved in

the development and function of hypothalamic POMC neurons (*124*) and has previously been implicated in regulation of the adipokine adiponectin (*125*), both of which provide plausible, if speculative, mechanistic links to energy homeostasis.

Rare variants were enriched within *CDH8* in both the UKB BMI analysis and SCOOP (Fig. 2B, D). In UKB the combined effect size of rare protein truncating variants (2.21 kg/m^2, p = 0.0025, Fig. 2B) was predominantly driven by one frameshift variant of large effect (16:61653821:CA>C, L729X carried by three individuals, $\beta = 12.76 \pm 2.54$kg/m^2, $p = 4.90\text{x}10^{-7}$, Fig. 2E) and the association was attenuated after the exclusion of this variant. In SCOOP there was a nominally significant enrichment for rare, predicted deleterious variants in *CDH8*, each in a single heterozygous proband (p = 0.045, Fig. 2A), specifically *CDH8* p.R479* (also detected in a single heterozygous individual in gnomAD, Table S11), *CDH8* p.R431W and p.R126W (both absent from gnomAD, Table S11). Pedigree analysis of affected families showed imperfect segregation of these variants with obesity (Table S14).

CDH8 encodes a brain-specific cadherin which has been shown to have a role in forming the anatomical and functional features critical for the formation of action-outcome associations (*126*). There are also >90 GWAS associations with CDH8 in GWAS catalog (*94*), many of which are for traits related to behavior or educational attainment. Given the importance of the central nervous system in maintaining energy homeostasis (*2*), and the well described links between obesity and both eating behavior and educational attainment (*59*), the links we report between BCS/BMI and obesity seem credible.

At the fifth locus, the canine association lay within *SEMA3D* for which we found no new human obesity association. The gene is a member of the Semaphorin 3 gene family previously implicated in causing severe, early onset human obesity and which have been shown to affect the development of hypothalamic neurons (*127*). In the same study, knockout of *SEMA3D* caused an increased body fat percentage in zebrafish. A single patient from the SOPP cohort was homozygous for a deleterious *SEMA3D* variant which was rare in control populations (*SEMA3D* p.D380H, Table S11). She had a BMI of 36 at 15 years old and her normal weight parents were both heterozygous for the variant.

We considered whether the rarity of deleterious variants in the five candidate genes might indicate they were under purifying selection, and whether they might have been overlooked as obesity genes because they caused rare, complex syndromes. We used pLI (*121*) and LOUEF (*36*) constraint metrics to demonstrate that three of the genes of interest (*CARD11, CDH8, CSNK1A1*) show evidence of purifying selection but the others (*DENND1B* and *SEMA3D*) do not (Table S15). None of the five genes are currently listed as known developmental disorder genes in the Developmental Disorders Genotype-to-Phenotype (DDG2P) database (*97*), a curated resource linking genetic variants with developmental disorders based on evidence from clinical and genetic studies. We have yet no explanation for the rarity of the *DENND1B* or *SEMA3D* mutations reported and further phenotypic characterization in animal models and other patient cohorts is needed.

CARD11 is a known cancer-causing gene, from tier 1 of the Cancer Gene Census (*128*). *CSNK1A1, DENND1B* and *SEMA3D* have all in the past been implicated in cancer pathogenesis, for example by being differentially regulated in certain cancer types, but are not listed in the Cancer Gene Census (*129–133*). We are not aware of any association between *CDH8* and cancer, although other members of the cadherin family are frequently implicated in metastatic

progression in cancer. In this context, it is of note that there was no history of cancer in the human variant carriers for any of the genes, nor any dogs included in the GWAS or follow-on studies (for which absence of systemic disease was an inclusion criteria).

Canine polygenic risk score application and utility

The Labrador PRS predicted BCS and body weight in a closely related breed, the golden retriever (n = 1765), although to a lesser extent. In more distantly related breeds (flat-coated retrievers, n = 196, pugs, n = 139), it was not predictive (Fig. 4A) (*134*). This reflected that in golden retrievers, 4 SNPs retained a significant effect on BCS, but none were significant in flat-coated retrievers or pugs (Table S13).

Polygenic risk score contributes to known within-breed variation

Labrador retrievers can be registered as one of three colors, black, yellow, and chocolate, with chocolate dogs being the most obesity-prone and food motivated (*28*). Chocolate Labradors in the discovery GWAS had higher BCS than other colors (mean BCS = 6.2 versus 5.5, $p < 0.05$, Table S2), as has been reported previously (*28, 135*). We considered whether this might be because the owners of chocolate colored dogs managed them less strictly (for instance, because they are popular family dogs). Using the DORA questionnaire (*24*) to quantify owner control of diet and exercise we showed there was no significant difference in owner management of diet and exercise between dogs of different coat colors (ANOVA $p = 0.95$). However, chocolate colored dogs had significantly higher PRS than dogs of other colors (mean PRS: red = 5.46, black = 6.41, yellow = 6.66, chocolate = 7.38, Fig. 4C). MDS plots reveal subtle clustering of chocolate dogs within the pet population which may explain this higher PRS (Fig. S11A).

Purpose-bred assistance Labradors had higher BCS than those kept as pets (mean BCS: assistance = 5.99, pet = 5.48, p <0.001). This was associated with significantly higher PRS (mean PRS assistance = 8.36, mean PRS pet = 6.32, p = 2x10-12, Fig. 4B). When assistance dogs were included in MDS plots, stratification is evident as compared to the pet Labradors (Fig. S11B). No assistance dogs were included in the discovery GWAS.

PRS, food motivation and gene environment interaction

Healthy weight Labradors were predominantly from the middle or lower tertiles of PRS. The majority of dogs which were obese were from the highest PRS tertile, with the remainder from the middle tertile (Fig. 4F). In 248 Labradors, the PRS positively predicted food motivation score (quantified as per materials and methods, Fig. 4D, $p = 0.03$) suggesting increased food-seeking behavior drives higher food intake and BCS in genetically predisposed dogs. Using a validated, owner-reported questionnaire we captured information regarding dogs' activity levels (during 'walks', whilst working, performing agility etc) and about the stringency with which owners controlled their dogs' access to food (*24*). For dogs in the highest two quintiles of PRS, more assiduous management of diet and exercise mitigated the development of increased BCS ($\beta = -0.020$, $p = 0.0077$) but it had no significant effect on BCS ($\beta = -0.007$, $p = 0.14$) in dogs in the lowest two quintiles (Fig. 4E). Reflecting this, PRS and owner control of the food and exercise environment displayed a significant interaction within the linear model ($p = 0.0028$).

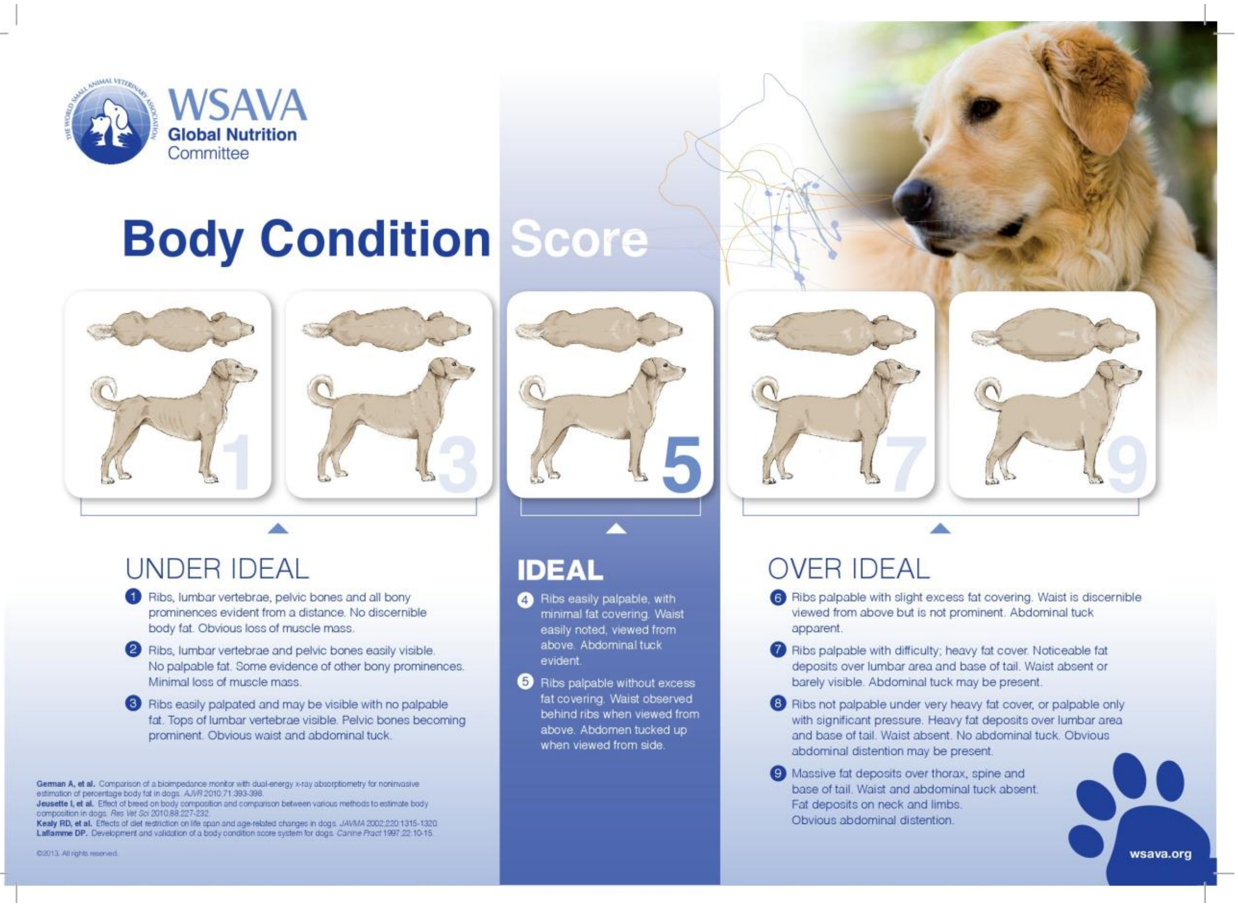

Fig. S1.

Body condition scoring descriptors and images used for scoring adiposity in dogs. The chart in is part of the Global Nutrition Committee Toolkit provided courtesy of the World Small Animal Veterinary Association.

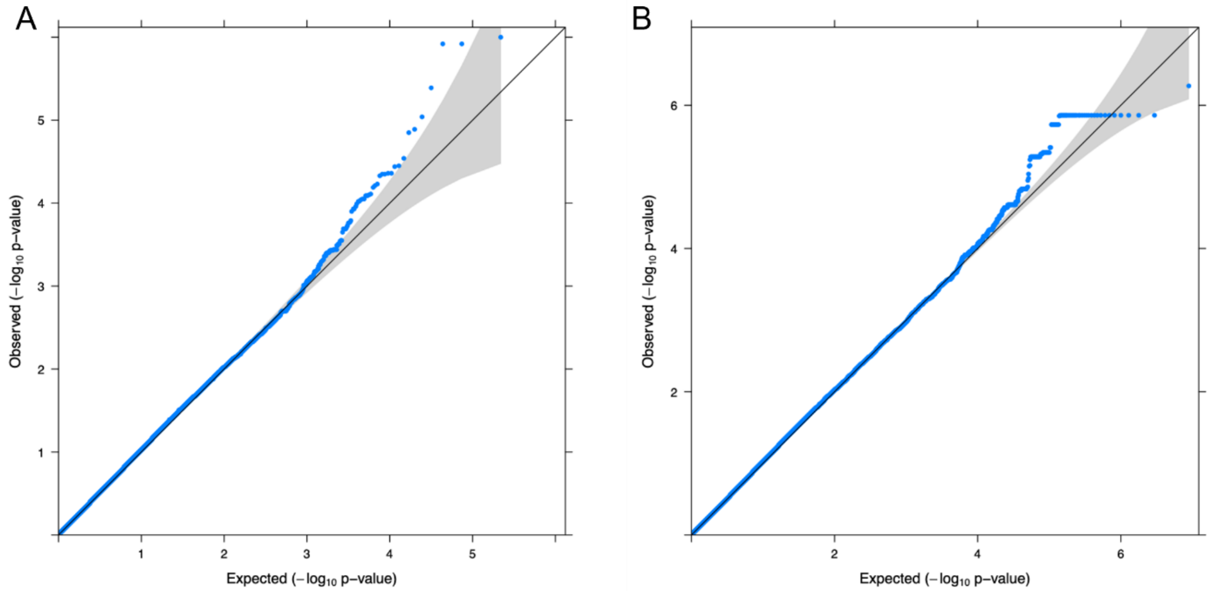

Fig. S2.

Quintile-Quintile (QQ) plot resulting from the GWAS in Labrador retrievers for body condition score (BCS) in 241 individuals. Generated LMM GWAS output applying GCTA MLMA-LOCO, using only genotypic data which were (A) directly genotyped or (B) including imputed genotypes.

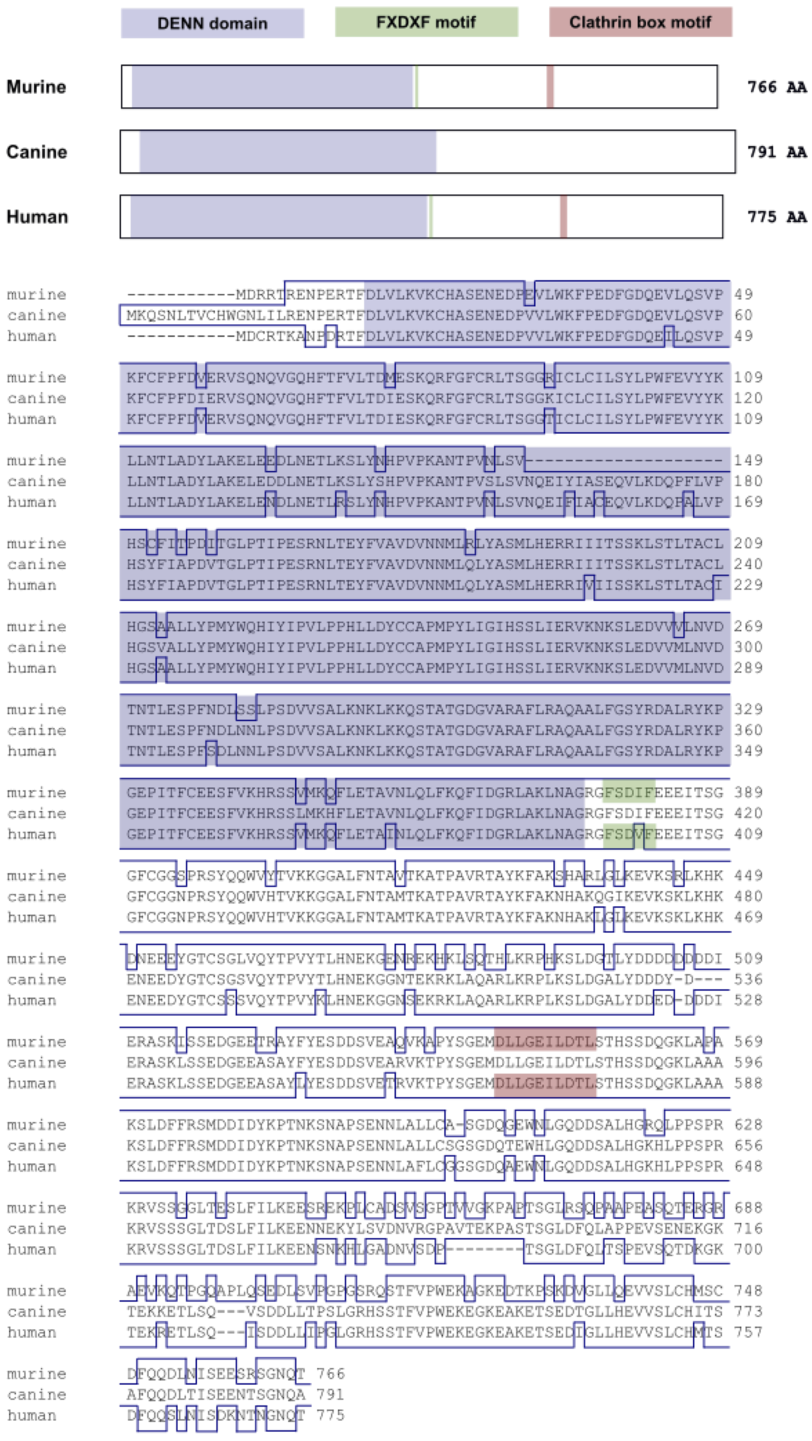

Fig. S3.

There is high homology between canine DENND1B and human and murine forms. Protein alignment of all three species is shown. Overall, canine has highest homology with human (89.4%) compared to mouse (82.7%), with near perfect homology in the functionally important DENN domain and within the FXDXF and clathrin box motifs.

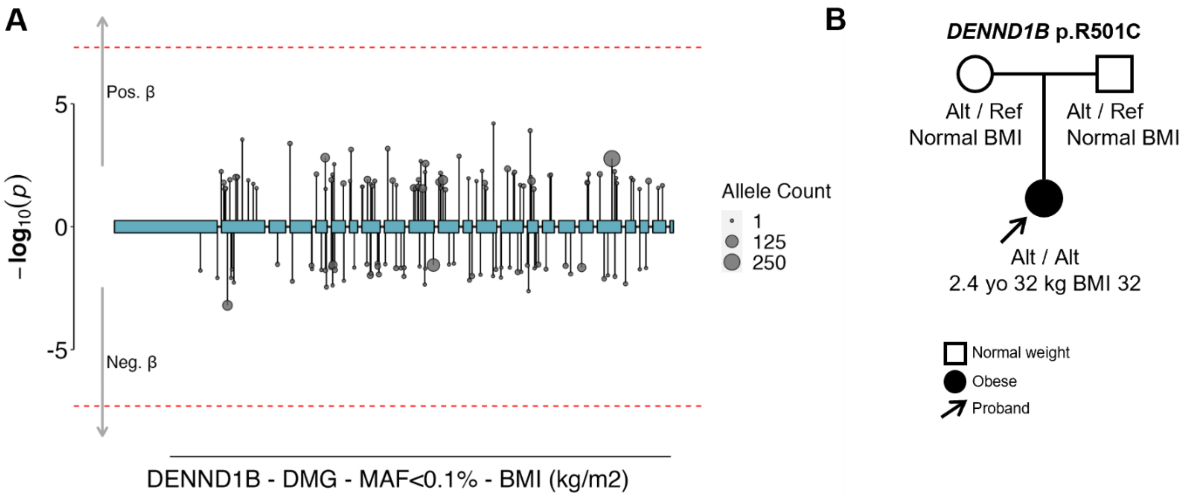

Fig. S4.

DENND1B associations with human obesity. (A) There was a nominally significant enrichment of damaging mutations in *DENND1B* with BMI in UK Biobank ($p = 0.0087$). Qualifying variants had a minor allele frequency (MAF) < 0.1% and were annotated as either high-confidence protein truncating variants or missense variants with a high CADD score (≥25). Each variant association is represented by a circle and vertical line, the line length indicates the *p*-value (-log10), in the direction of its effect on BMI in carriers of the rare allele, and the circle size indicates the number of carriers of each variant (allele count). The horizontal red lines indicate *p*-values smaller than $1.6x10^{-6}$. Exons are indicated by the blue boxes. (B) *DENND1B* p.R501C was identified in a single proband in the Severe Obesity Pakistani Population (SOPP) cohort whose normal weight parents were heterozygous for the variant. No alternative genetic diagnosis was revealed by exome sequencing.

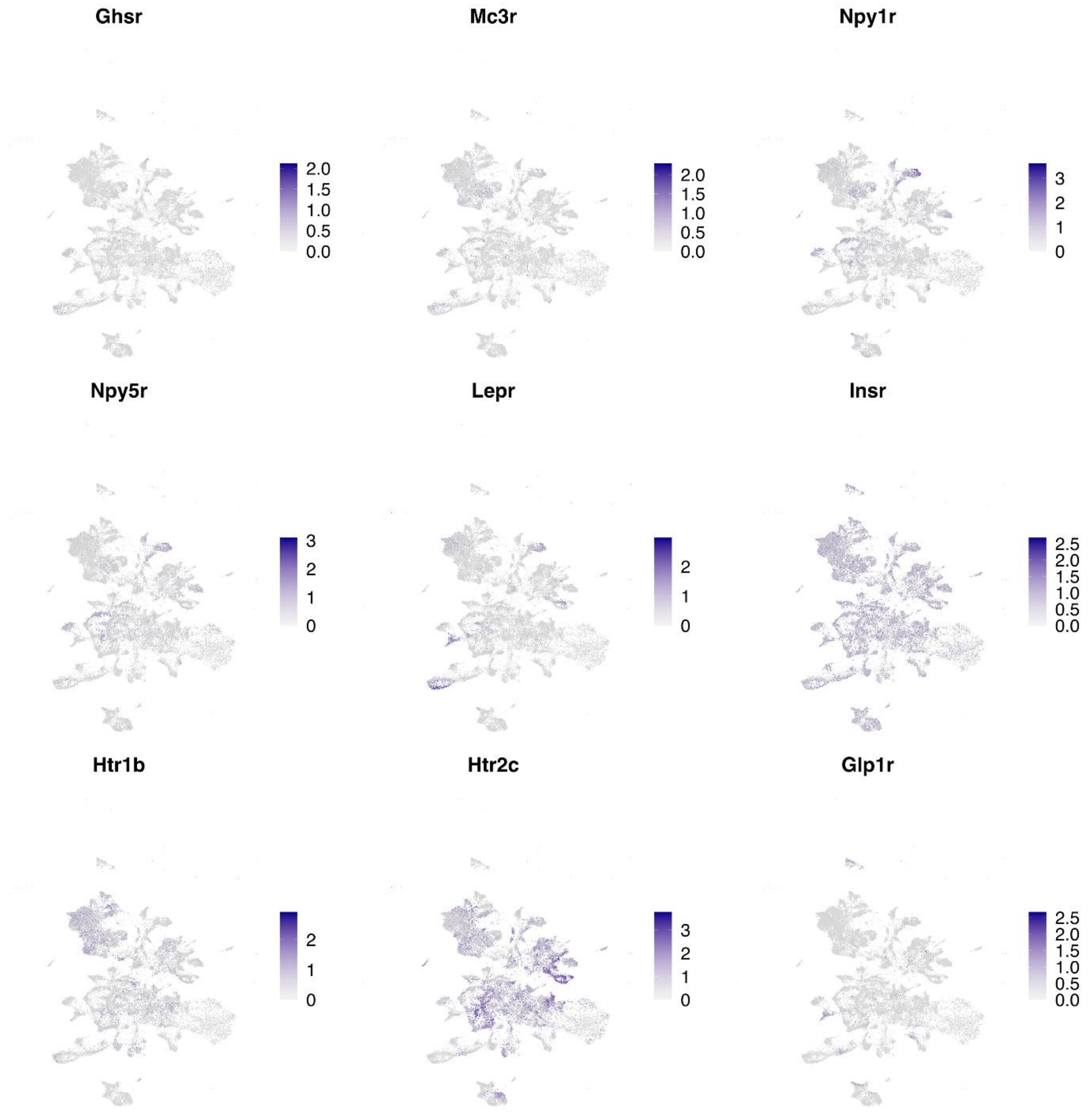

Fig. S5.

Dennd1b is widely expressed in the murine hypothalamus (HypoMap) and is co-expressed with multiple receptors involved in energy homeostasis. UMAP plots show co-expression of *Dennd1b* with *Ghsr*, *Mc3r*, *Npy1r*, *Npy5r*, *Lepr*, *Insr*, *Htr1b*, *Htr2c*, and *Glp1r*. UMAP plots show expression of each receptor in the subset of neurons from HypoMap that expressed *Dennd1b* transcripts.

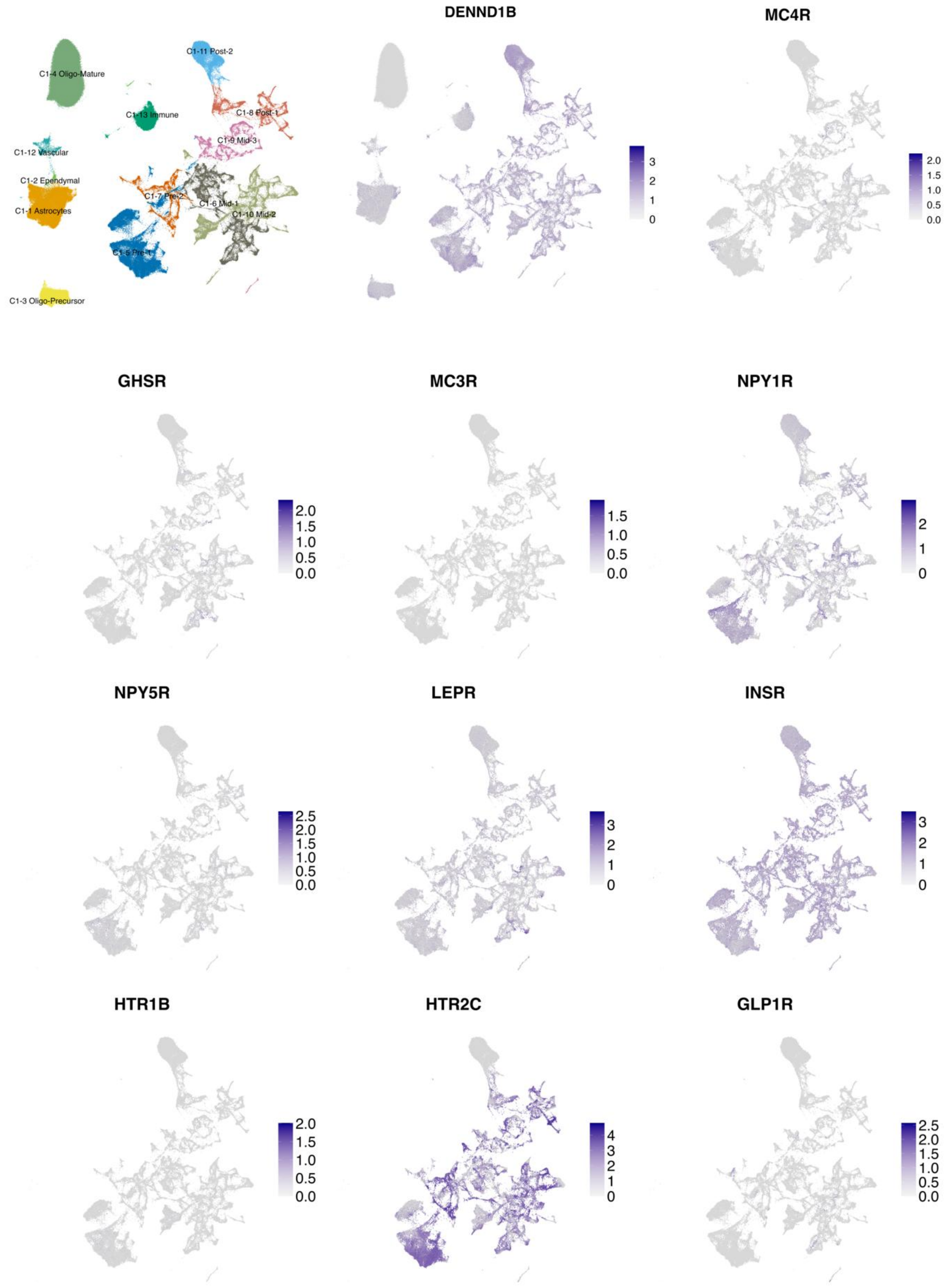

C1-4 Oligo-Mature
C1-11 Post-2
C1-13 Immune
C1-8 Post-1
C1-9 Mid-3
C1-12 Vascular
C1-2 Ependymal
C1-7 Pre-2
C1-6 Mid-1
C1-1 Astrocytes
C1-10 Mid-2
C1-5 Pre-1
C1-3 Oligo-Precursor
DENND1B
MC4R
GHSR
MC3R
NPY1R
NPY5R
LEPR
INSR
HTR1B
HTR2C
GLP1R

Fig. S6.

RNAseq shows *DENND1B* is widely expressed in the human hypothalamus and is co-expressed with *MC4R*. UMAP plots show human HYPOMAP single nucleus RNA sequencing data extracted from Tadross et al., 2023 (*43*) colored by general cell type (C1 clustering level), log-normalized expression of *DENND1B* in the human hypothalamus and log-normalized expression of *MC4R* and other hypothalamic receptors in the *DENND1B+* neuronal subset.

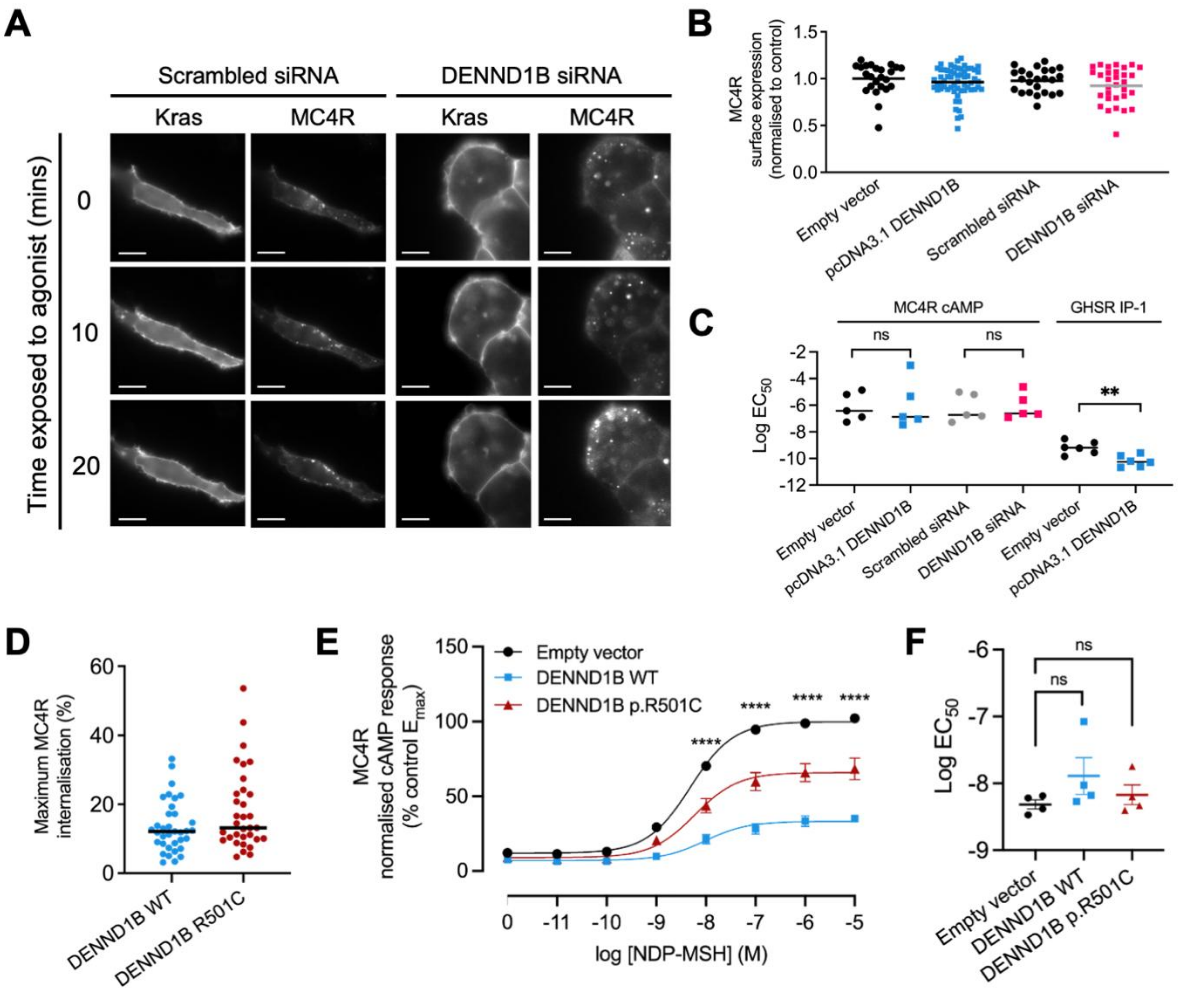

Fig. S7

Additional data on DENND1B effect on MC4R. (A) Representative HILO images of HEK293 with DENND1B knocked down (KD) using siRNA or control condition (scrambled siRNA). MC4R (SNAP-647) and plasma membrane marker Kras (Venus) were expressed and show colocalization at time point 0. Decreased ligand-induced MC4R internalization was observed in conditions of DENND1B knockdown (p = 0.044). Scale bar = 5 μm. (B) Basal MC4R surface expression (via colocalization with Kras) was quantified at time point 0 in control cells (empty vector – EV, scrambled siRNA), cells overexpressing DENND1B and cells in which DENND1B was knocked down using siRNA. Control and DENND1B conditions were not significantly different from one another (pairwise comparisons to internal control). (C) MC4R cAMP and GHSR IP-1 EC_{50} for dose response curves. No significant difference was observed between the control and either DENND1B overexpression or knockdown for MC4R. Overexpression of DENND1B significantly reduced the GHSR IP-1 log EC50 compared to the control (p = 0.004**). Data from 5-6 independent experiments. Conditions compared using a student's t test. (D) There was no difference in maximum internalization of MC4R after overexpression of DENND1B WT or p.R501C (determined by localization to cell surface Kras in HILO images after 20 min α-MSH-stimulation). Results expressed as the percentage of the unstimulated receptor surface expression. (E) Ligand-induced cAMP response of MC4R in HEK293 cells after

overexpression of WT DENND1B or DENND1B p.R501C. Each condition was significantly different to the other at the highest four concentrations of ligand (two-way ANOVA with multiple comparisons, p <0.0001****). (F) There was no difference between the log EC_{50} of the dose response for the same experiments (4 independent experiments, one-way ANOVA multiple comparisons).

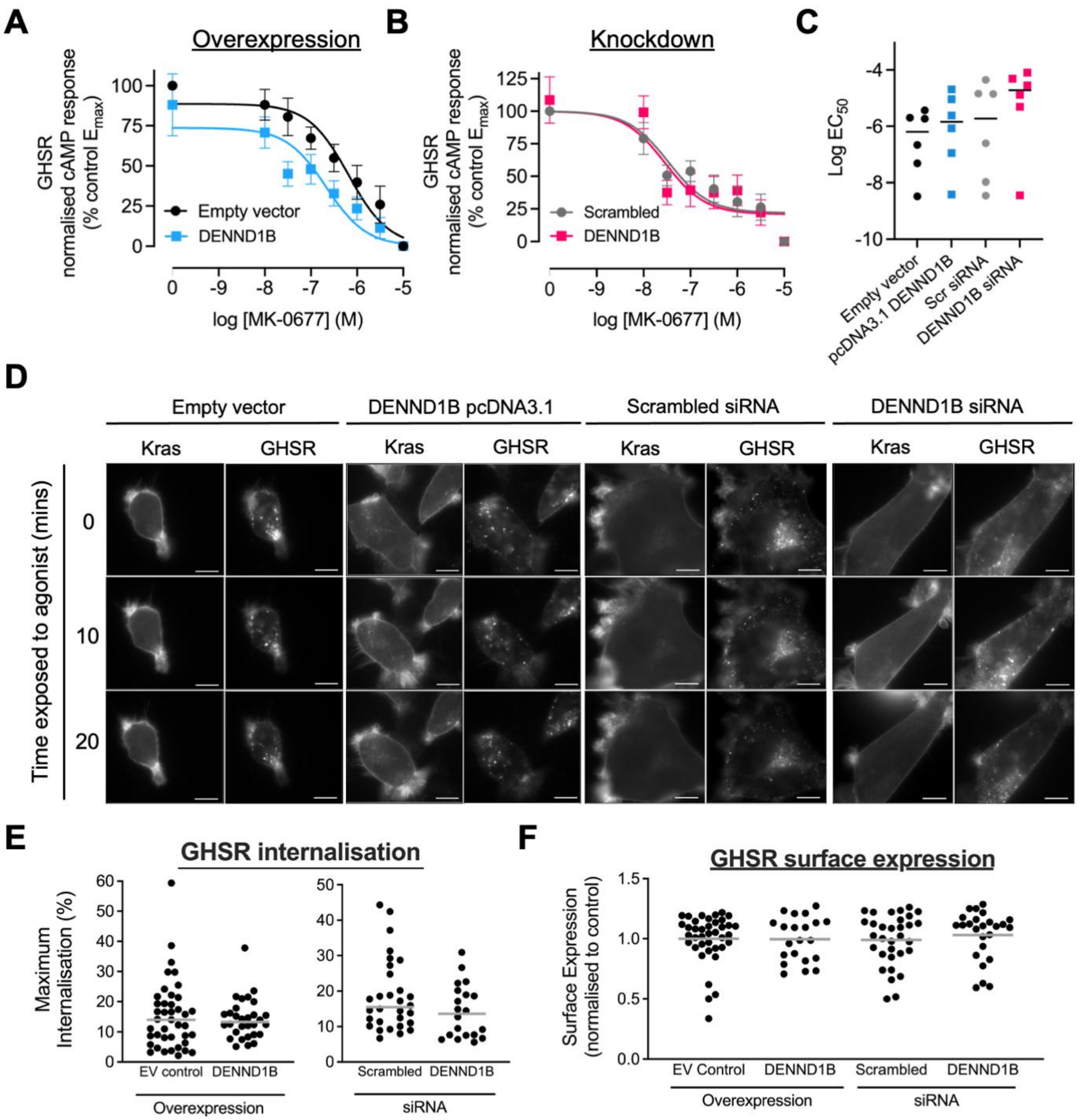

Fig. S8

Altering DENDD1B expression had no effect on GHSR cAMP signaling internalization or cell surface expression. GHSR-activated decrease in cAMP production in HEK293 cells expressing GHSR is shown in conditions of (A) DENND1B overexpression or (b) DENND1B siRNA knockdown (KD), after prior stimulation with forskolin. (C) Log EC_{50} from 6 independent experiments were compared (Student's t test, no significant differences). (D) Representative HILO images of HEK293 cells expressing GHSR (SNAP-647) and plasma membrane marker Kras (Venus) showing colocalization at time point 0, with GHSR internalisation following agonist stimulation. Scale bar = 5 µm. There was no difference between (E) internalisation or (F) basal GHSR surface expression at time point 0 between control cells (empty vector – EV, scrambled siRNA) versus cells overexpressing DENND1B or with DENND1B knockdown using siRNA (pairwise comparisons).

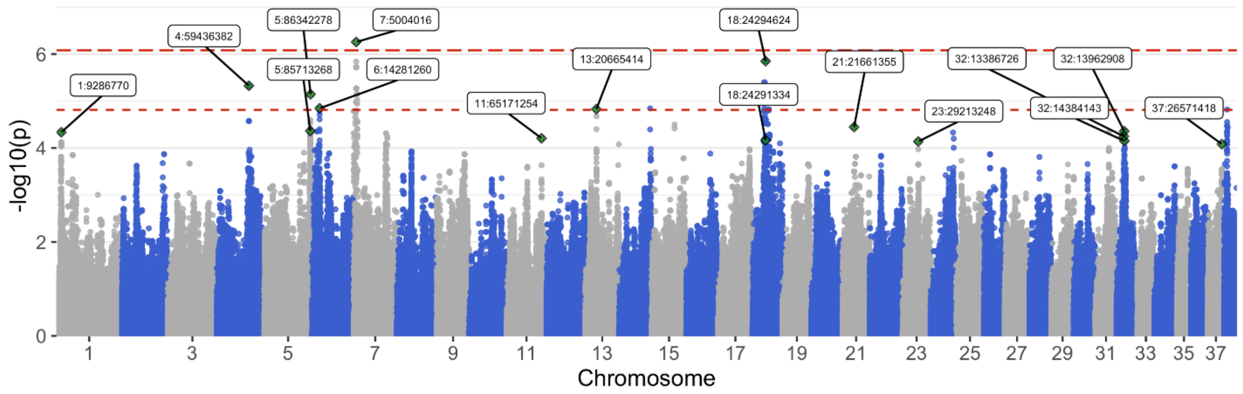

Fig. S9

Using the GWAS results, a polygenic score method was created using the 'clumping and thresholding' technique. First, an arbitrary *p* value significance threshold was allocated to the GWAS output ($p < 0.0004$) retaining only SNPs which passed this threshold (n = 481). Variants which had a pairwise r^2 measure of LD ≥ 0.7 were treated as the same locus, with the most significant 'lead' SNP per locus taken forward to the PRS calculation. Using this approach, 23 SNPs contributed to the primary PRS. Polygenic score was calculated as the sum of the product of each 'risk' allele genotype weighted by effect size from GWAS output. PRS was then refined in the secondary/test set of Labrador retrievers (n = 350). We retained only SNPs which retained a positive effect size for BCS outcome in the test set (significant association was not a requirement). The 16 remaining SNP retained in the final PRS are shown.

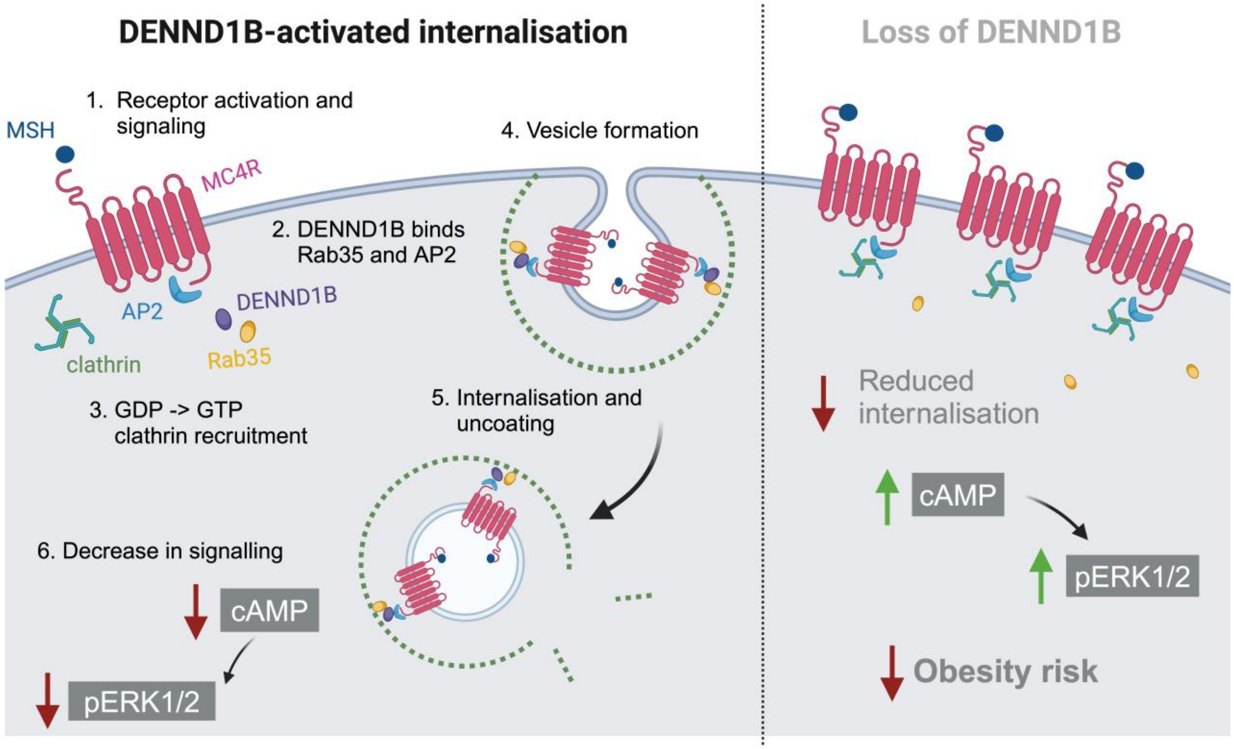

Fig. S10
Proposed mechanism linking regulation of obesity risk to DENND1B to energy homeostasis by regulation of MC4R signaling. Hypothalamic MC4R integrate signals from POMC and AgRP neurons to regulate energy homeostasis, exerting an anorexigenic effect. (1) α-MSH binds MC4R, activating signaling via Gs protein recruitment. Signaling is inactivated by β-arrestin recruitment triggering recruitment of clathrin machinery (AP-2 and clathrin). (2) DENND1B binds AP-2 and Rab35 and DENND1B GEF activity converts GDP to GTP and activates Rab35. (3) This initiates Rab35-dependent (4) vesicle formation and (5) MC4R endocytosis. (6) This downmodulation of MC4R surface expression reduces its signaling. The internalized MC4R vesicle is then clathrin uncoated. Right hand side: Reduced DENND1B impairs ligand-activated internalization and prolongs anorexigenic signaling to decrease risk of obesity. AP-2 – AP2 adaptor complex, GDP – guanosine diphosphate, GTP – Guanosine triphosphate, GEF – Guanine nucleotide exchange factors, cAMP – Cyclic adenosine monophosphate, pERK1/2 - active extracellular signal-regulated kinase 1/2. Figure created using BioRender.com.

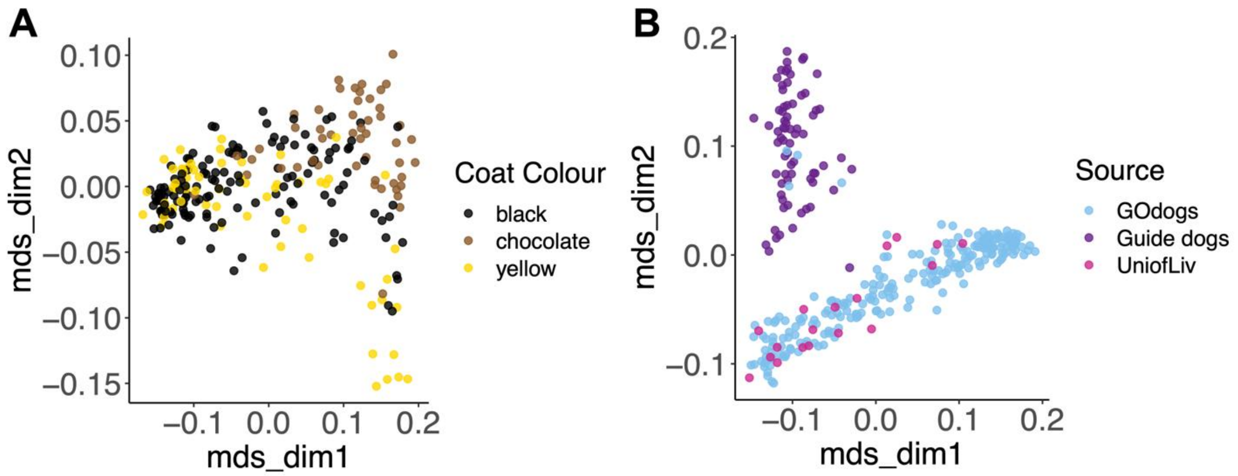

Fig. S11

MDS plots of Labrador population. Dimensional reduction plots presenting the genetic structure of the Labrador population in two dimensions. (A) The discovery GWAS set of dogs (n = 241) with points colored by coat color (black, chocolate, yellow). (B) Points are colored by source of data (GOdogs research group internal recruitment; assistance dogs collected through collaboration with Guide Dogs UK; UniofLiv, pet dogs through collaboration with the University of Liverpool). Only dogs which were identified as pets and which clustered with the larger pet population cluster were included in the discovery GWAS. The four pet dogs which clustered with assistance dogs were discovered (after owner interviews) to be retired or 'failed' guide dogs and were excluded from the discovery GWAS. MDS – multi-dimensional scaling; mds_dim – MDS dimension.

Table S1 – S15

Table S1: Items included in the Dog Obesity Risk Assessment (DORA) Questionnaire which contributed to owner control and food motivation scores (24). Owners respond on a 4 or 5 point Likert scale according to their level of agreement with each statement and responses are translated into a percentage. Overall food motivation score or owner control score are calculated as the mean response to the items within that category. For some items, the scores are reversed (as indicated) to ensure item scores are in the congruent direction for the overall factor score.

Table S2: Summary statistics for phenotypes in the Labrador populations reported in the study. In the discovery GWAS all dogs were kept as companions. In the wider population (used for examining the utility of PRS) there were also assistance dogs in the population. For each group, the summary statistics are provided for all dogs, and subgroups by role and color. 'Red' is not a recognized color for this breed in the Kennel Club registration system (considered officially as yellow) but was reported by owners and so is listed separately where it occurred. PRS, polygenic risk score; SD, standard deviation; BCS, body condition score.

Table S3: Regions of interest from BCS GWAS. Regions of interest tagged by lead SNP variant (in format chromosome:position) from GWAS for BCS via two methods: r2 measure of LD regions ≥ 0.8 (80%) or haplotype mapping using PLINK. The 'refined' region was either the mapped haplotype plus in-phase SNPs or, if the SNP did not map to a haploblock, the r2 ≥ 0.8 region.

Table S4: Candidate causative variants identified through analysis of high coverage whole genome sequencing data from 25 Labrador retrievers. Labrador WGS (>30x) BAM files were visualized in IGV to detect structural variants using the 'soft clip' function which identified multiple regions suggestive of the presence of a moderate to large insertion or deletion mutation which are denoted below as 'poorly defined indels'. GWAS region of interest is categorized by top SNP from each locus. Distance from tagging SNP is determined by position of SNP or first bp of insertion/deletion. Ref/Alt indicates reference or alternative base on the Canfam3.1 genome. Control/risk label is determined based on whether the variant is inherited with the risk or non-risk allele for the lead GWAS SNP for BCS in the Labrador GWAS (for >70% of alleles).

Table S5: Results of investigation of positional candidate genes for evidence of effects related to obesity. PheWAS data were interrogated in GWAS Atlas (https://atlas.ctglab.nl/PheWAS). To investigate the effect of gene knockout or other genetic manipulation in mouse, we used web portals of the International Mouse Phenotyping Consortium (https://www.mousephenotype.org/), the Mouse Phenome Database (https://phenome.jax.org/) and performed searches using PubMed. The Online Mendelian Inheritance in Man (OMIM) database was searched to determine whether mutations in each gene were known to cause Mendelian disease in humans. The GTEx portal (https://www.gtexportal.org/) and Human Protein Atlas (https://www.proteinatlas.org/) were interrogated to determine tissues in which gene expression was enriched. - indicates no phenotype of interest. N/A indicates data not available.

Table S6: Summary of human BMI associations for genes identified on the canine GWAS.

Table S7: Predicted causative gene for obesity-related traits in human loci syntenic to canine obesity GWAS loci. We interrogated human GWAS data on body mass index (BMI) and waist-hip ratio (WHR) adjusted for BMI in up to 806,834 and 694,649 individuals respectively from the GIANT study and circulating high density lipoprotein (HDL) and triglyceride levels in up to 1,253,277 European individuals from the Global Lipids Genetics Consortium (GLGC) study (up to n = 1,253,277). G2G uses a variety of information to predict causative gene at each locus. G2G – GWAS to Genes; GWAS – genome wide association study; BMI – body mass index; HDL – high density lipoprotein; WHR – waist-hip ratio; PoPS – polygenic priority score; pQTL – protein quantitative trait loci; eQTL – expression quantitative trait loci; ABC – activity by contact; MAGMA – Multi-marker Analysis of GenoMic Annotation; LD – linkage disequilibrium.

Table S8: Canine GWAS obesity candidate genes are expressed in a range of canine tissues. Data obtained from the BarkBase database *(41)* and expressed in fragments per kilobase of transcript per million read pairs (FPKM). NA not in database.

Table S9: Candidate gene expression in single cell RNA sequencing from mouse and human hypothalamus. We interrogated HypoMap, a unified single cell gene expression atlas of the mouse hypothalamus (42) and snRNAseq of the human hypothalamus in data from the human HYPOMAP (43). Results shown for each gene of interest show expression in all cell types, all neurons, Pomc-expressing cells and Mc4r-expressing cells. GHSR is included since it was used, like MC4R, in molecular studies of DENND1B function. Presented as a percentage of all cells in subgroup to 2 decimal places.

Table S10: Co-expression of DENND1B and other candidate genes with POMC, MC4R and GHSR in the human hypothalamus. In snRNAseq data from the human hypothalamus, cells were clustered according to gene expression patterns (42). The table shows cell type and gene markers defining each cluster along with the number of nuclei contributing to that data. Data shown: % <gene>+ (% of cells / cluster expressing gene); AverageExpression_<gene> (Average log-normalized expression of gene / cluster); % <gene1>+<gene2>+ (Percentage of cells / cluster co-expressing both genes).

Table S11: Rare variants predicted to be deleterious and found to be associated with obesity in either SCOOP or SOPP data sets. To test if regions and genes identified on the canine GWAS were also relevant to human obesity, we examined regions orthologous to those mapped in dogs in human data sets. Specifically, we tested for enrichment of rare (MAF <0.0026%), predicted deleterious (CADD ≥ 25) variants in the Severe Childhood Onset Obesity Project (SCOOP-UK) (35) (n = 982), compared to reference exomes of similar ancestry (gnomAD v2.1.1, n = 56,885); and investigated the Severe Obesity in Pakistani Population (SOPP) cohort of patients with severe, early onset obesity from a highly consanguineous population, all with unaffected parents and no prior genetic diagnosis (*44*, *45*), in which we focused on homozygous, predicted deleterious variants. The table shows variants identified using that approach and their frequency in different control populations including gNomad Non-Finnish European (NFE), NCBI Allele Frequency Aggregator (ALFA), Trans-Omics for Precision Medicine (TOPMed) participants and the 1000 Genomes database.

Table S12: Predictive value of PRS demonstrated by improved goodness of fit for obesity-related traits in Labrador retrievers when it is included in the model. The R^2 measure for goodness-of-fit within linear regression models is displayed for the combined Labrador retriever population and subgroups, for models predicting three obesity-related traits: BCS, body weight (kg) and food motivation. R^2 is displayed in its adjusted and unadjusted forms. All numbers rounded to 2sf. Food motivation on a scale of 1-100%. PRS – polygenic risk score; R^2 – linear regression goodness-of-fit measure; BCS – body condition score on a 9-point scale.

Table S13: PRS variant associations across purebred groups. Results of linear regression for each PRS SNP variant that contributed to the Labrador retriever PRS in other breed and population-specific minimal models. Data presented for each purebred cohort and phenotype combination as effect size β and significance p. Phenotypes are BCS, body weight (kg) and DORA food motivation.

Table S14: Clinical information and genotypes of probands and available family members from human patients with variants in CDH8 and CSNK1A1. Families delineated by background color. BMI SDS, body mass index standard deviation score (Z score).

Table S15: Results of tests for purifying selection. Two constraint metrics were used to evaluate the intolerance of a gene to high confidence predicted loss of function (pLoF) mutations in the gnomAD database. Results beyond the thresholds suggested by the developers of each metric (<0.4 for LOUEF and >0.9 for pLI) are in bold text. o/e, observed/expected.

References and Notes

1. A. A. van der Klaauw, I. S. Farooqi, The hunger genes: Pathways to obesity. *Cell* **161**, 119–132 (2015). doi:10.1016/j.cell.2015.03.008 Medline

2. R. J. F. Loos, G. S. H. Yeo, The genetics of obesity: From discovery to biology. *Nat. Rev. Genet.* **23**, 120–133 (2022). doi:10.1038/s41576-021-00414-z Medline

3. N. B. Sutter, M. A. Eberle, H. G. Parker, B. J. Pullar, E. F. Kirkness, L. Kruglyak, E. A. Ostrander, Extensive and breed-specific linkage disequilibrium in *Canis familiaris*. *Genome Res.* **14**, 2388–2396 (2004). doi:10.1101/gr.3147604 Medline

4. K. Lindblad-Toh, C. M. Wade, T. S. Mikkelsen, E. K. Karlsson, D. B. Jaffe, M. Kamal, M. Clamp, J. L. Chang, E. J. Kulbokas 3rd, M. C. Zody, E. Mauceli, X. Xie, M. Breen, R. K. Wayne, E. A. Ostrander, C. P. Ponting, F. Galibert, D. R. Smith, P. J. DeJong, E. Kirkness, P. Alvarez, T. Biagi, W. Brockman, J. Butler, C.-W. Chin, A. Cook, J. Cuff, M. J. Daly, D. DeCaprio, S. Gnerre, M. Grabherr, M. Kellis, M. Kleber, C. Bardeleben, L. Goodstadt, A. Heger, C. Hitte, L. Kim, K.-P. Koepfli, H. G. Parker, J. P. Pollinger, S. M. J. Searle, N. B. Sutter, R. Thomas, C. Webber, Broad Sequencing Platform Members, E. S. Lander, Genome sequence, comparative analysis and haplotype structure of the domestic dog. *Nature* **438**, 803–819 (2005). doi:10.1038/nature04338 Medline

5. J. V. Halo, A. L. Pendleton, F. Shen, A. J. Doucet, T. Derrien, C. Hitte, L. E. Kirby, B. Myers, E. Sliwerska, S. Emery, J. V. Moran, A. R. Boyko, J. M. Kidd, Long-read assembly of a Great Dane genome highlights the contribution of GC-rich sequence and mobile elements to canine genomes. *Proc. Natl. Acad. Sci. U.S.A.* **118**, e2016274118 (2021). doi:10.1073/pnas.2016274118 Medline

6. C. Wang, O. Wallerman, M.-L. Arendt, E. Sundström, Å. Karlsson, J. Nordin, S. Mäkeläinen, G. R. Pielberg, J. Hanson, Å. Ohlsson, S. Saellström, H. Rönnberg, I. Ljungvall, J. Häggström, T. F. Bergström, Å. Hedhammar, J. R. S. Meadows, K. Lindblad-Toh, A novel canine reference genome resolves genomic architecture and uncovers transcript complexity. *Commun. Biol.* **4**, 185 (2021). doi:10.1038/s42003-021-01698-x Medline

7. J. R. S. Meadows, J. M. Kidd, G.-D. Wang, H. G. Parker, P. Z. Schall, M. Bianchi, M. J. Christmas, K. Bougiouri, R. M. Buckley, C. Hitte, A. K. Nguyen, C. Wang, V. Jagannathan, J. E. Niskanen, L. A. F. Frantz, M. Arumilli, S. Hundi, K. Lindblad-Toh, C. Ginja, K. K. Agustina, C. André, A. R. Boyko, B. W. Davis, M. Drögemüller, X.-Y. Feng, K. Gkagkavouzis, G. Iliopoulos, A. C. Harris, M. K. Hytönen, D. C. Kalthoff, Y.-H. Liu, P. Lymberakis, N. Poulakakis, A. E. Pires, F. Racimo, F. Ramos-Almodovar, P. Savolainen, S. Venetsani, I. Tammen, A. Triantafyllidis, B. vonHoldt, R. K. Wayne, G. Larson, F. W. Nicholas, H. Lohi, T. Leeb, Y.-P. Zhang, E. A. Ostrander, Genome sequencing of 2000 canids by the Dog10K consortium advances the understanding of demography, genome function and architecture. *Genome Biol.* **24**, 187 (2023). doi:10.1186/s13059-023-03023-7 Medline

8. E. V. Dutrow, J. A. Serpell, E. A. Ostrander, Domestic dog lineages reveal genetic drivers of behavioral diversification. *Cell* **185**, 4737–4755.e18 (2022). doi:10.1016/j.cell.2022.11.003 Medline

9. J. Donner, J. Freyer, S. Davison, H. Anderson, M. Blades, L. Honkanen, L. Inman, C. A. Brookhart-Knox, A. Louviere, O. P. Forman, R. Chodroff Foran, Genetic prevalence and clinical relevance of canine Mendelian disease variants in over one million dogs. *PLOS Genet.* **19**, e1010651 (2023). doi:10.1371/journal.pgen.1010651 Medline

10. E. M. Lund, P. J. Armstrong, C. A. Kirk, J. S. Klausner, Prevalence and risk factors for obesity in adult dogs from private US veterinary practices. *Intern. J. Appl. Res. Vet. Med.* **4**, 177–186 (2006).

11. C. Pegram, E. Raffan, E. White, A. H. Ashworth, D. C. Brodbelt, D. B. Church, D. G. O'Neill, Frequency, breed predisposition and demographic risk factors for overweight status in dogs in the UK. *J. Small Anim. Pract.* **62**, 521–530 (2021). doi:10.1111/jsap.13325 Medline

12. J. F. Summers, D. G. O'Neill, D. Church, L. Collins, D. Sargan, D. C. Brodbelt, Health-related welfare prioritisation of canine disorders using electronic health records in primary care practice in the UK. *BMC Vet. Res.* **15**, 163 (2019). doi:10.1186/s12917-019-1902-0 Medline

13. A. J. German, G. R. T. Woods, S. L. Holden, L. Brennan, C. Burke, Dangerous trends in pet obesity. *Vet. Rec.* **182**, 25 (2018). doi:10.1136/vr.k2 Medline

14. E. A. Courcier, R. M. Thomson, D. J. Mellor, P. S. Yam, An epidemiological study of environmental factors associated with canine obesity. *J. Small Anim. Pract.* **51**, 362–367 (2010). doi:10.1111/j.1748-5827.2010.00933.x Medline

15. A. J. German, The growing problem of obesity in dogs and cats. *J. Nutr.* **136**, 1940S–1946S (2006). doi:10.1093/jn/136.7.1940S Medline

16. N. Wallis, E. Raffan, The Genetic Basis of Obesity and Related Metabolic Diseases in Humans and Companion Animals. *Genes* **11**, 1378 (2020). doi:10.3390/genes11111378 Medline

17. E. Raffan, The big problem: Battling companion animal obesity. *Vet. Rec.* **173**, 287–291 (2013). doi:10.1136/vr.f5815 Medline

18. S. Sheet, S. Krishnamoorthy, J. Cha, S. Choi, B.-H. Choi, Identification of Candidate Genes and Pathways Associated with Obesity-Related Traits in Canines via Gene-Set Enrichment and Pathway-Based GWAS Analysis. *Animals* **10**, 2071 (2020). doi:10.3390/ani10112071 Medline

19. E. Raffan, R. J. Dennis, C. J. O'Donovan, J. M. Becker, R. A. Scott, S. P. Smith, D. J. Withers, C. J. Wood, E. Conci, D. N. Clements, K. M. Summers, A. J. German, C. S. Mellersh, M. L. Arendt, V. P. Iyemere, E. Withers, J. Söder, S. Wernersson, G. Andersson, K. Lindblad-Toh, G. S. H. Yeo, S. O'Rahilly, A Deletion in the Canine *POMC* Gene Is Associated with Weight and Appetite in Obesity-Prone Labrador Retriever Dogs. *Cell Metab.* **23**, 893–900 (2016). doi:10.1016/j.cmet.2016.04.012 Medline

20. M. T. Dittmann, G. Lakatos, J. F. Wainwright, J. Mokrosinski, E. Cross, I. S. Farooqi, N. J. Wallis, L. G. Halsey, R. Wilson, S. O'Rahilly, G. S. H. Yeo, E. Raffan, Low resting metabolic rate and increased hunger due to β-MSH and β-endorphin deletion in a canine model. *Sci. Adv.* **10**, eadj3823 (2024). doi:10.1126/sciadv.adj3823 Medline

21. H. Biebermann, T. R. Castañeda, F. van Landeghem, A. von Deimling, F. Escher, G. Brabant, J. Hebebrand, A. Hinney, M. H. Tschöp, A. Grüters, H. Krude, A role for β-melanocyte-stimulating hormone in human body-weight regulation. *Cell Metab.* **3**, 141–146 (2006). doi:10.1016/j.cmet.2006.01.007 Medline

22. Y. S. Lee, B. G. Challis, D. A. Thompson, G. S. H. Yeo, J. M. Keogh, M. E. Madonna, V. Wraight, M. Sims, V. Vatin, D. Meyre, J. Shield, C. Burren, Z. Ibrahim, T. Cheetham, P. Swift, A. Blackwood, C.-C. C. Hung, N. J. Wareham, P. Froguel, G. L. Millhauser, S. O'Rahilly, I. S. Farooqi, A POMC variant implicates β-melanocyte-stimulating hormone in the control of human energy balance. *Cell Metab.* **3**, 135–140 (2006). doi:10.1016/j.cmet.2006.01.006 Medline

23. G. S. H. Yeo, D. H. M. Chao, A.-M. Siegert, Z. M. Koerperich, M. D. Ericson, S. E. Simonds, C. M. Larson, S. Luquet, I. Clarke, S. Sharma, K. Clément, M. A. Cowley, C. Haskell-Luevano, L. Van Der Ploeg, R. A. H. Adan, The melanocortin pathway and energy homeostasis: From discovery to obesity therapy. *Mol. Metab.* **48**, 101206 (2021). doi:10.1016/j.molmet.2021.101206 Medline

24. E. Raffan, S. P. Smith, S. O'Rahilly, J. Wardle, Development, factor structure and application of the Dog Obesity Risk and Appetite (DORA) questionnaire. *PeerJ* **3**, e1278 (2015). doi:10.7717/peerj.1278 Medline

25. A. J. German, S. L. Holden, G. L. Moxham, K. L. Holmes, R. M. Hackett, J. M. Rawlings, A simple, reliable tool for owners to assess the body condition of their dog or cat. *J. Nutr.* **136**, 2031S–2033S (2006). doi:10.1093/jn/136.7.2031S Medline

26. D. P. Laflamme, Development and validation of a body condition score system for dogs. *Canine Pract.* **22**, 10–15 (1997).

27. D. I. Mawby, J. W. Bartges, A. d'Avignon, D. P. Laflamme, T. D. Moyers, T. Cottrell, Comparison of various methods for estimating body fat in dogs. *J. Am. Anim. Hosp. Assoc.* **40**, 109–114 (2004). doi:10.5326/0400109 Medline

28. N. J. Wallis, N. T. Sumanasekera, E. Raffan, Obesity risk factors in British Labrador retrievers: Effect of sex, neuter status, age, chocolate coat colour and food motivation. *Vet. Rec.* **194**, e3410 (2024). doi:10.1002/vetr.3410 Medline

29. I. N. Kieler, S. M. Persson, R. Hagman, V. D. Marinescu, Å. Hedhammar, E. Strandberg, K. Lindblad-Toh, M. L. Arendt, Genome wide association study in Swedish Labrador retrievers identifies genetic loci associated with hip dysplasia and body weight. *Sci. Rep.* **14**, 6090 (2024). doi:10.1038/s41598-024-56060-y Medline

30. M. L. Arendt, M. Melin, N. Tonomura, M. Koltookian, C. Courtay-Cahen, N. Flindall, J. Bass, K. Boerkamp, K. Megquir, L. Youell, S. Murphy, C. McCarthy, C. London, G. R. Rutteman, M. Starkey, K. Lindblad-Toh, Genome-Wide Association Study of Golden Retrievers Identifies Germ-Line Risk Factors Predisposing to Mast Cell Tumours. *PLOS Genet.* **11**, e1005647 (2015). doi:10.1371/journal.pgen.1005647 Medline

31. L. Yengo, J. Sidorenko, K. E. Kemper, Z. Zheng, A. R. Wood, M. N. Weedon, T. M. Frayling, J. Hirschhorn, J. Yang, P. M. Visscher, GIANT Consortium, Meta-analysis of genome-wide association studies for height and body mass index in ~700000 individuals

of European ancestry. *Hum. Mol. Genet.* **27**, 3641–3649 (2018). doi:10.1093/hmg/ddy271 Medline

32. C. Sudlow, J. Gallacher, N. Allen, V. Beral, P. Burton, J. Danesh, P. Downey, P. Elliott, J. Green, M. Landray, B. Liu, P. Matthews, G. Ong, J. Pell, A. Silman, A. Young, T. Sprosen, T. Peakman, R. Collins, UK biobank: An open access resource for identifying the causes of a wide range of complex diseases of middle and old age. *PLOS Med.* **12**, e1001779 (2015). doi:10.1371/journal.pmed.1001779 Medline

33. L. R. Kaisinger, K. A. Kentistou, S. Stankovic, E. J. Gardner, F. R. Day, Y. Zhao, A. Mörseburg, C. J. Carnie, G. Zagnoli-Vieira, F. Puddu, S. P. Jackson, S. O'Rahilly, I. S. Farooqi, L. Dearden, L. C. Pantaleão, S. E. Ozanne, K. K. Ong, J. R. B. Perry, Large-scale exome sequence analysis identifies sex- and age-specific determinants of obesity. *Cell Genom.* **3**, 100362 (2023). doi:10.1016/j.xgen.2023.100362 Medline

34. Y. Zhao, M. Chukanova, K. A. Kentistou, Z. Fairhurst-Hunter, A. M. Siegert, R. Y. Jia, G. K. C. Dowsett, E. J. Gardner, K. Lawler, F. R. Day, L. R. Kaisinger, Y. L. Tung, B. Y. H. Lam, H. C. Chen, Q. Wang, J. Berumen-Campos, P. Kuri-Morales, R. Tapia-Conyer, J. Alegre-Diaz, I. Barroso, J. Emberson, J. M. Torres, R. Collins, D. Saleheen, K. R. Smith, D. S. Paul, F. Merkle, I. S. Farooqi, N. J. Wareham, S. Petrovski, S. O'Rahilly, K. K. Ong, G. S. H. Yeo, J. R. B. Perry, Protein-truncating variants in *BSN* are associated with severe adult-onset obesity, type 2 diabetes and fatty liver disease. *Nat. Genet.* **56**, 579–584 (2024). doi:10.1038/s41588-024-01694-x Medline

35. E. Wheeler, N. Huang, E. G. Bochukova, J. M. Keogh, S. Lindsay, S. Garg, E. Henning, H. Blackburn, R. J. F. Loos, N. J. Wareham, S. O'Rahilly, M. E. Hurles, I. Barroso, I. S. Farooqi, Genome-wide SNP and CNV analysis identifies common and low-frequency variants associated with severe early-onset obesity. *Nat. Genet.* **45**, 513–517 (2013). doi:10.1038/ng.2607 Medline

36. K. J. Karczewski, L. C. Francioli, G. Tiao, B. B. Cummings, J. Alföldi, Q. Wang, R. L. Collins, K. M. Laricchia, A. Ganna, D. P. Birnbaum, L. D. Gauthier, H. Brand, M. Solomonson, N. A. Watts, D. Rhodes, M. Singer-Berk, E. M. England, E. G. Seaby, J. A. Kosmicki, R. K. Walters, K. Tashman, Y. Farjoun, E. Banks, T. Poterba, A. Wang, C. Seed, N. Whiffin, J. X. Chong, K. E. Samocha, E. Pierce-Hoffman, Z. Zappala, A. H. O'Donnell-Luria, E. V. Minikel, B. Weisburd, M. Lek, J. S. Ware, C. Vittal, I. M. Armean, L. Bergelson, K. Cibulskis, K. M. Connolly, M. Covarrubias, S. Donnelly, S. Ferriera, S. Gabriel, J. Gentry, N. Gupta, T. Jeandet, D. Kaplan, C. Llanwarne, R. Munshi, S. Novod, N. Petrillo, D. Roazen, V. Ruano-Rubio, A. Saltzman, M. Schleicher, J. Soto, K. Tibbetts, C. Tolonen, G. Wade, M. E. Talkowski, Genome Aggregation Database Consortium, B. M. Neale, M. J. Daly, D. G. MacArthur, The mutational constraint spectrum quantified from variation in 141,456 humans. *Nature* **581**, 434–443 (2020). doi:10.1038/s41586-020-2308-7 Medline

37. S. Yoshimura, A. Gerondopoulos, A. Linford, D. J. Rigden, F. A. Barr, Family-wide characterization of the DENN domain Rab GDP-GTP exchange factors. *J. Cell Biol.* **191**, 367–381 (2010). doi:10.1083/jcb.201008051 Medline

38. J. Nasser, D. T. Bergman, C. P. Fulco, P. Guckelberger, B. R. Doughty, T. A. Patwardhan, T. R. Jones, T. H. Nguyen, J. C. Ulirsch, F. Lekschas, K. Mualim, H. M. Natri, E. M.

Weeks, G. Munson, M. Kane, H. Y. Kang, A. Cui, J. P. Ray, T. M. Eisenhaure, R. L. Collins, K. Dey, H. Pfister, A. L. Price, C. B. Epstein, A. Kundaje, R. J. Xavier, M. J. Daly, H. Huang, H. K. Finucane, N. Hacohen, E. S. Lander, J. M. Engreitz, Genome-wide enhancer maps link risk variants to disease genes. *Nature* **593**, 238–243 (2021). doi:10.1038/s41586-021-03446-x Medline

39. K. A. Kentistou, L. R. Kaisinger, S. Stankovic, M. Vaudel, E. Mendes de Oliveira, A. Messina, R. G. Walters, X. Liu, A. S. Busch, H. Helgason, D. J. Thompson, F. Santoni, K. M. Petricek, Y. Zouaghi, I. Huang-Doran, D. F. Gudbjartsson, E. Bratland, K. Lin, E. J. Gardner, Y. Zhao, R. Y. Jia, C. Terao, M. J. Riggan, M. K. Bolla, M. Yazdanpanah, N. Yazdanpanah, J. P. Bradfield, L. Broer, A. Campbell, D. I. Chasman, D. L. Cousminer, N. Franceschini, L. H. Franke, G. Girotto, C. He, M.-R. Järvelin, P. K. Joshi, Y. Kamatani, R. Karlsson, J. Luan, K. L. Lunetta, R. Mägi, M. Mangino, S. E. Medland, C. Meisinger, R. Noordam, T. Nutile, M. P. Concas, O. Polašek, E. Porcu, S. M. Ring, C. Sala, A. V. Smith, T. Tanaka, P. J. van der Most, V. Vitart, C. A. Wang, G. Willemsen, M. Zygmunt, T. U. Ahearn, I. L. Andrulis, H. Anton-Culver, A. C. Antoniou, P. L. Auer, C. L. K. Barnes, M. W. Beckmann, A. Berrington de Gonzalez, N. V. Bogdanova, S. E. Bojesen, H. Brenner, J. E. Buring, F. Canzian, J. Chang-Claude, F. J. Couch, A. Cox, L. Crisponi, K. Czene, M. B. Daly, E. W. Demerath, J. Dennis, P. Devilee, I. De Vivo, T. Dörk, A. M. Dunning, M. Dwek, J. G. Eriksson, P. A. Fasching, L. Fernandez-Rhodes, L. Ferreli, O. Fletcher, M. Gago-Dominguez, M. García-Closas, J. A. García-Sáenz, A. González-Neira, H. Grallert, P. Guénel, C. A. Haiman, P. Hall, U. Hamann, H. Hakonarson, R. J. Hart, M. Hickey, M. J. Hooning, R. Hoppe, J. L. Hopper, J.-J. Hottenga, F. B. Hu, H. Huebner, D. J. Hunter, ABCTB Investigators, H. Jernström, E. M. John, D. Karasik, E. K. Khusnutdinova, V. N. Kristensen, J. V. Lacey, D. Lambrechts, L. J. Launer, P. A. Lind, A. Lindblom, P. K. E. Magnusson, A. Mannermaa, M. I. McCarthy, T. Meitinger, C. Menni, K. Michailidou, I. Y. Millwood, R. L. Milne, G. W. Montgomery, H. Nevanlinna, I. M. Nolte, D. R. Nyholt, N. Obi, K. M. O’Brien, K. Offit, A. J. Oldehinkel, S. R. Ostrowski, A. Palotie, O. B. Pedersen, A. Peters, G. Pianigiani, D. Plaseska-Karanfilska, A. Pouta, A. Pozarickij, P. Radice, G. Rennert, F. R. Rosendaal, D. Ruggiero, E. Saloustros, D. P. Sandler, S. Schipf, C. O. Schmidt, M. K. Schmidt, K. Small, B. Spedicati, M. Stampfer, J. Stone, R. M. Tamimi, L. R. Teras, E. Tikkanen, C. Turman, C. M. Vachon, Q. Wang, R. Winqvist, A. Wolk, B. S. Zemel, W. Zheng, K. W. van Dijk, B. Z. Alizadeh, S. Bandinelli, E. Boerwinkle, D. I. Boomsma, M. Ciullo, G. Chenevix-Trench, F. Cucca, T. Esko, C. Gieger, S. F. A. Grant, V. Gudnason, C. Hayward, I. Kolčić, P. Kraft, D. A. Lawlor, N. G. Martin, E. A. Nøhr, N. L. Pedersen, C. E. Pennell, P. M. Ridker, A. Robino, H. Snieder, U. Sovio, T. D. Spector, D. Stöckl, C. Sudlow, N. J. Timpson, D. Toniolo, A. Uitterlinden, S. Ulivi, H. Völzke, N. J. Wareham, E. Widen, J. F. Wilson, Lifelines Cohort Study, Danish Blood Donor Study, Ovarian Cancer Association Consortium, Breast Cancer Association Consortium, Biobank Japan Project, China Kadoorie Biobank Collaborative Group, P. D. P. Pharoah, L. Li, D. F. Easton, P. R. Njølstad, P. Sulem, J. M. Murabito, A. Murray, D. Manousaki, A. Juul, C. Erikstrup, K. Stefansson, M. Horikoshi, Z. Chen, I. S. Farooqi, N. Pitteloud, S. Johansson, F. R. Day, J. R. B. Perry, K. K. Ong, Understanding the genetic complexity of puberty timing across the allele frequency spectrum. *Nat. Genet.* **56**, 1397–1411 (2024). doi:10.1038/s41588-024-01798-4 Medline

40. A. L. Marat, P. S. McPherson, The connecdenn family, Rab35 guanine nucleotide exchange factors interfacing with the clathrin machinery. *J. Biol. Chem.* **285**, 10627–10637 (2010). doi:10.1074/jbc.M109.050930 Medline

41. K. Megquier, D. P. Genereux, J. Hekman, R. Swofford, J. Turner-Maier, J. Johnson, J. Alonso, X. Li, K. Morrill, L. J. Anguish, M. Koltookian, B. Logan, C. R. Sharp, L. Ferrer, K. Lindblad-Toh, V. N. Meyers-Wallen, A. Hoffman, E. K. Karlsson, BarkBase: Epigenomic Annotation of Canine Genomes. *Genes* **10**, 433 (2019). doi:10.3390/genes10060433 Medline

42. L. Steuernagel, B. Y. H. Lam, P. Klemm, G. K. C. Dowsett, C. A. Bauder, J. A. Tadross, T. S. Hitschfeld, A. Del Rio Martin, W. Chen, A. J. de Solis, H. Fenselau, P. Davidsen, I. Cimino, S. N. Kohnke, D. Rimmington, A. P. Coll, A. Beyer, G. S. H. Yeo, J. C. Brüning, HypoMap—A unified single-cell gene expression atlas of the murine hypothalamus. *Nat. Metab.* **4**, 1402–1419 (2022). doi:10.1038/s42255-022-00657-y Medline

43. J. A. Tadross, L. Steuernagel, G. K. C. Dowsett, K. A. Kentistou, S. Lundh, M. Porniece, P. Klemm, K. Rainbow, H. Hvid, K. Kania, J. Polex-Wolf, L. Bjerre Knudsen, C. Pyke, J. R. B. Perry, B. Y. H. Lam, J. C. Brüning, G. S. H. Yeo, A comprehensive spatio-cellular map of the human hypothalamus. *Nature* 10.1038/s41586-024-08504-8 (2025). doi:10.1038/s41586-024-08504-8 Medline

44. S. Saeed, Q. M. Janjua, A. Haseeb, R. Khanam, E. Durand, E. Vaillant, L. Ning, A. Badreddine, L. Berberian, M. Boissel, S. Amanzougarene, M. Canouil, M. Derhourhi, A. Bonnefond, M. Arslan, P. Froguel, Rare Variant Analysis of Obesity-Associated Genes in Young Adults With Severe Obesity From a Consanguineous Population of Pakistan. *Diabetes* **71**, 694–705 (2022). doi:10.2337/db21-0373 Medline

45. S. Saeed, M. Arslan, J. Manzoor, S. M. Din, Q. M. Janjua, H. Ayesha, Q.-T. Ain, L. Inam, S. Lobbens, E. Vaillant, E. Durand, M. Derhourhi, S. Amanzougarene, A. Badreddine, L. Berberian, S. Gaget, W. I. Khan, T. A. Butt, A. Bonnefond, P. Froguel, Genetic Causes of Severe Childhood Obesity: A Remarkably High Prevalence in an Inbred Population of Pakistan. *Diabetes* **69**, 1424–1438 (2020). doi:10.2337/db19-1238 Medline

46. S. W. Choi, T. S.-H. Mak, P. F. O'Reilly, Tutorial: A guide to performing polygenic risk score analyses. *Nat. Protoc.* **15**, 2759–2772 (2020). doi:10.1038/s41596-020-0353-1 Medline

47. C.-W. Yang, C. D. Hojer, M. Zhou, X. Wu, A. Wuster, W. P. Lee, B. L. Yaspan, A. C. Chan, Regulation of T Cell Receptor Signaling by DENND1B in T_H2 Cells and Allergic Disease. *Cell* **164**, 141–155 (2016). doi:10.1016/j.cell.2015.11.052 Medline

48. P. D. Allaire, A. L. Marat, C. Dall'Armi, G. Di Paolo, P. S. McPherson, B. Ritter, The Connecdenn DENN domain: A GEF for Rab35 mediating cargo-specific exit from early endosomes. *Mol. Cell* **37**, 370–382 (2010). doi:10.1016/j.molcel.2009.12.037 Medline

49. B. Brouwers, E. M. de Oliveira, M. Marti-Solano, F. B. F. Monteiro, S.-A. Laurin, J. M. Keogh, E. Henning, R. Bounds, C. A. Daly, S. Houston, V. Ayinampudi, N. Wasiluk, D. Clarke, B. Plouffe, M. Bouvier, M. M. Babu, I. S. Farooqi, J. Mokrosiński, Human MC4R variants affect endocytosis, trafficking and dimerization revealing multiple

cellular mechanisms involved in weight regulation. *Cell Rep.* **34**, 108862 (2021). doi:10.1016/j.celrep.2021.108862 Medline

50. G. S. H. Yeo, E. J. Lank, I. S. Farooqi, J. Keogh, B. G. Challis, S. O'Rahilly, Mutations in the human melanocortin-4 receptor gene associated with severe familial obesity disrupts receptor function through multiple molecular mechanisms. *Hum. Mol. Genet.* **12**, 561–574 (2003). doi:10.1093/hmg/ddg057 Medline

51. S. Ramachandrappa, A. Raimondo, A. M. G. Cali, J. M. Keogh, E. Henning, S. Saeed, A. Thompson, S. Garg, E. G. Bochukova, S. Brage, V. Trowse, E. Wheeler, A. E. Sullivan, M. Dattani, P. E. Clayton, V. Datta, J. B. Bruning, N. J. Wareham, S. O'Rahilly, D. J. Peet, I. Barroso, M. L. Whitelaw, I. S. Farooqi, Rare variants in single-minded 1 (*SIM1*) are associated with severe obesity. *J. Clin. Invest.* **123**, 3042–3050 (2013). doi:10.1172/JCI68016 Medline

52. M. Asai, S. Ramachandrappa, M. Joachim, Y. Shen, R. Zhang, N. Nuthalapati, V. Ramanathan, D. E. Strochlic, P. Ferket, K. Linhart, C. Ho, T. V. Novoselova, S. Garg, M. Ridderstråle, C. Marcus, J. N. Hirschhorn, J. M. Keogh, S. O'Rahilly, L. F. Chan, A. J. Clark, I. S. Farooqi, J. A. Majzoub, Loss of function of the melanocortin 2 receptor accessory protein 2 is associated with mammalian obesity. *Science* **341**, 275–278 (2013). doi:10.1126/science.1233000 Medline

53. M. Wang, X. Wang, B. Jiang, Y. Zhai, J. Zheng, L. Yang, X. Tai, Y. Li, S. Fu, J. Xu, X. Lei, Z. Kuang, C. Zhang, X. Bai, M. Li, T. Zan, S. Qu, Q. Li, C. Zhang, Identification of MRAP protein family as broad-spectrum GPCR modulators. *Clin. Transl. Med.* **12**, e1091 (2022). doi:10.1002/ctm2.1091 Medline

54. A. E. Locke, B. Kahali, S. I. Berndt, A. E. Justice, T. H. Pers, F. R. Day, C. Powell, S. Vedantam, M. L. Buchkovich, J. Yang, D. C. Croteau-Chonka, T. Esko, T. Fall, T. Ferreira, S. Gustafsson, Z. Kutalik, J. Luan, R. Mägi, J. C. Randall, T. W. Winkler, A. R. Wood, T. Workalemahu, J. D. Faul, J. A. Smith, J. H. Zhao, W. Zhao, J. Chen, R. Fehrmann, Å. K. Hedman, J. Karjalainen, E. M. Schmidt, D. Absher, N. Amin, D. Anderson, M. Beekman, J. L. Bolton, J. L. Bragg-Gresham, S. Buyske, A. Demirkan, G. Deng, G. B. Ehret, B. Feenstra, M. F. Feitosa, K. Fischer, A. Goel, J. Gong, A. U. Jackson, S. Kanoni, M. E. Kleber, K. Kristiansson, U. Lim, V. Lotay, M. Mangino, I. M. Leach, C. Medina-Gomez, S. E. Medland, M. A. Nalls, C. D. Palmer, D. Pasko, S. Pechlivanis, M. J. Peters, I. Prokopenko, D. Shungin, A. Stančáková, R. J. Strawbridge, Y. J. Sung, T. Tanaka, A. Teumer, S. Trompet, S. W. van der Laan, J. van Setten, J. V. Van Vliet-Ostaptchouk, Z. Wang, L. Yengo, W. Zhang, A. Isaacs, E. Albrecht, J. Ärnlöv, G. M. Arscott, A. P. Attwood, S. Bandinelli, A. Barrett, I. N. Bas, C. Bellis, A. J. Bennett, C. Berne, R. Blagieva, M. Blüher, S. Böhringer, L. L. Bonnycastle, Y. Böttcher, H. A. Boyd, M. Bruinenberg, I. H. Caspersen, Y. I. Chen, R. Clarke, E. W. Daw, A. J. M. de Craen, G. Delgado, M. Dimitriou, A. S. F. Doney, N. Eklund, K. Estrada, E. Eury, L. Folkersen, R. M. Fraser, M. E. Garcia, F. Geller, V. Giedraitis, B. Gigante, A. S. Go, A. Golay, A. H. Goodall, S. D. Gordon, M. Gorski, H.-J. Grabe, H. Grallert, T. B. Grammer, J. Gräßler, H. Grönberg, C. J. Groves, G. Gusto, J. Haessler, P. Hall, T. Haller, G. Hallmans, C. A. Hartman, M. Hassinen, C. Hayward, N. L. Heard-Costa, Q. Helmer, C. Hengstenberg, O. Holmen, J.-J. Hottenga, A. L. James, J. M. Jeff, Å. Johansson, J. Jolley, T. Juliusdottir, L. Kinnunen, W. Koenig, M. Koskenvuo, W. Kratzer, J. Laitinen, C.

Lamina, K. Leander, N. R. Lee, P. Lichtner, L. Lind, J. Lindström, K. S. Lo, S. Lobbens, R. Lorbeer, Y. Lu, F. Mach, P. K. E. Magnusson, A. Mahajan, W. L. McArdle, S. McLachlan, C. Menni, S. Merger, E. Mihailov, L. Milani, A. Moayyeri, K. L. Monda, M. A. Morken, A. Mulas, G. Müller, M. Müller-Nurasyid, A. W. Musk, R. Nagaraja, M. M. Nöthen, I. M. Nolte, S. Pilz, N. W. Rayner, F. Renstrom, R. Rettig, J. S. Ried, S. Ripke, N. R. Robertson, L. M. Rose, S. Sanna, H. Scharnagl, S. Scholtens, F. R. Schumacher, W. R. Scott, T. Seufferlein, J. Shi, A. V. Smith, J. Smolonska, A. V. Stanton, V. Steinthorsdottir, K. Stirrups, H. M. Stringham, J. Sundström, M. A. Swertz, A. J. Swift, A.-C. Syvänen, S.-T. Tan, B. O. Tayo, B. Thorand, G. Thorleifsson, J. P. Tyrer, H.-W. Uh, L. Vandenput, F. C. Verhulst, S. H. Vermeulen, N. Verweij, J. M. Vonk, L. L. Waite, H. R. Warren, D. Waterworth, M. N. Weedon, L. R. Wilkens, C. Willenborg, T. Wilsgaard, M. K. Wojczynski, A. Wong, A. F. Wright, Q. Zhang, LifeLines Cohort Study, E. P. Brennan, M. Choi, Z. Dastani, A. W. Drong, P. Eriksson, A. Franco-Cereceda, J. R. Gådin, A. G. Gharavi, M. E. Goddard, R. E. Handsaker, J. Huang, F. Karpe, S. Kathiresan, S. Keildson, K. Kiryluk, M. Kubo, J.-Y. Lee, L. Liang, R. P. Lifton, B. Ma, S. A. McCarroll, A. J. McKnight, J. L. Min, M. F. Moffatt, G. W. Montgomery, J. M. Murabito, G. Nicholson, D. R. Nyholt, Y. Okada, J. R. B. Perry, R. Dorajoo, E. Reinmaa, R. M. Salem, N. Sandholm, R. A. Scott, L. Stolk, A. Takahashi, T. Tanaka, F. M. van 't Hooft, A. A. E. Vinkhuyzen, H.-J. Westra, W. Zheng, K. T. Zondervan, ADIPOGen Consortium, AGEN-BMI Working Group, CARDIOGRAMplusC4D Consortium, CKDGen Consortium, GLGC, ICBP, MAGIC Investigators, MuTHER Consortium, MIGen Consortium, PAGE Consortium, ReproGen Consortium, GENIE Consortium, International Endogene Consortium, A. C. Heath, D. Arveiler, S. J. L. Bakker, J. Beilby, R. N. Bergman, J. Blangero, P. Bovet, H. Campbell, M. J. Caulfield, G. Cesana, A. Chakravarti, D. I. Chasman, P. S. Chines, F. S. Collins, D. C. Crawford, L. A. Cupples, D. Cusi, J. Danesh, U. de Faire, H. M. den Ruijter, A. F. Dominiczak, R. Erbel, J. Erdmann, J. G. Eriksson, M. Farrall, S. B. Felix, E. Ferrannini, J. Ferrières, I. Ford, N. G. Forouhi, T. Forrester, O. H. Franco, R. T. Gansevoort, P. V. Gejman, C. Gieger, O. Gottesman, V. Gudnason, U. Gyllensten, A. S. Hall, T. B. Harris, A. T. Hattersley, A. A. Hicks, L. A. Hindorff, A. D. Hingorani, A. Hofman, G. Homuth, G. K. Hovingh, S. E. Humphries, S. C. Hunt, E. Hyppönen, T. Illig, K. B. Jacobs, M.-R. Jarvelin, K.-H. Jöckel, B. Johansen, P. Jousilahti, J. W. Jukema, A. M. Jula, J. Kaprio, J. J. P. Kastelein, S. M. Keinanen-Kiukaanniemi, L. A. Kiemeney, P. Knekt, J. S. Kooner, C. Kooperberg, P. Kovacs, A. T. Kraja, M. Kumari, J. Kuusisto, T. A. Lakka, C. Langenberg, L. L. Marchand, T. Lehtimäki, V. Lyssenko, S. Männistö, A. Marette, T. C. Matise, C. A. McKenzie, B. McKnight, F. L. Moll, A. D. Morris, A. P. Morris, J. C. Murray, M. Nelis, C. Ohlsson, A. J. Oldehinkel, K. K. Ong, P. A. F. Madden, G. Pasterkamp, J. F. Peden, A. Peters, D. S. Postma, P. P. Pramstaller, J. F. Price, L. Qi, O. T. Raitakari, T. Rankinen, D. C. Rao, T. K. Rice, P. M. Ridker, J. D. Rioux, M. D. Ritchie, I. Rudan, V. Salomaa, N. J. Samani, J. Saramies, M. A. Sarzynski, H. Schunkert, P. E. H. Schwarz, P. Sever, A. R. Shuldiner, J. Sinisalo, R. P. Stolk, K. Strauch, A. Tönjes, D.-A. Trégouët, A. Tremblay, E. Tremoli, J. Virtamo, M.-C. Vohl, U. Völker, G. Waeber, G. Willemsen, J. C. Witteman, M. C. Zillikens, L. S. Adair, P. Amouyel, F. W. Asselbergs, T. L. Assimes, M. Bochud, B. O. Boehm, E. Boerwinkle, S. R. Bornstein, E. P. Bottinger, C. Bouchard, S. Cauchi, J. C. Chambers, S. J. Chanock, R. S. Cooper, P. I. W. de Bakker, G. Dedoussis, L. Ferrucci, P. W. Franks, P. Froguel, L. C. Groop, C. A.

Haiman, A. Hamsten, J. Hui, D. J. Hunter, K. Hveem, R. C. Kaplan, M. Kivimaki, D. Kuh, M. Laakso, Y. Liu, N. G. Martin, W. März, M. Melbye, A. Metspalu, S. Moebus, P. B. Munroe, I. Njølstad, B. A. Oostra, C. N. A. Palmer, N. L. Pedersen, M. Perola, L. Pérusse, U. Peters, C. Power, T. Quertermous, R. Rauramaa, F. Rivadeneira, T. E. Saaristo, D. Saleheen, N. Sattar, E. E. Schadt, D. Schlessinger, P. E. Slagboom, H. Snieder, T. D. Spector, U. Thorsteinsdottir, M. Stumvoll, J. Tuomilehto, A. G. Uitterlinden, M. Uusitupa, P. van der Harst, M. Walker, H. Wallaschofski, N. J. Wareham, H. Watkins, D. R. Weir, H.-E. Wichmann, J. F. Wilson, P. Zanen, I. B. Borecki, P. Deloukas, C. S. Fox, I. M. Heid, J. R. O'Connell, D. P. Strachan, K. Stefansson, C. M. van Duijn, G. R. Abecasis, L. Franke, T. M. Frayling, M. I. McCarthy, P. M. Visscher, A. Scherag, C. J. Willer, M. Boehnke, K. L. Mohlke, C. M. Lindgren, J. S. Beckmann, I. Barroso, K. E. North, E. Ingelsson, J. N. Hirschhorn, R. J. F. Loos, E. K. Speliotes, Genetic studies of body mass index yield new insights for obesity biology. *Nature* **518**, 197–206 (2015). doi:10.1038/nature14177 Medline

55. F. Seyednasrollah, J. Mäkelä, N. Pitkänen, M. Juonala, N. Hutri-Kähönen, T. Lehtimäki, J. Viikari, T. Kelly, C. Li, L. Bazzano, L. L. Elo, O. T. Raitakari, Prediction of Adulthood Obesity Using Genetic and Childhood Clinical Risk Factors in the Cardiovascular Risk in Young Finns Study. *Circ. Cardiovasc. Genet.* **10**, e001554 (2017). doi:10.1161/CIRCGENETICS.116.001554 Medline

56. P. R. Jansen, N. Vos, J. van Uhm, I. A. Dekkers, R. van der Meer, M. M. A. M. Mannens, M. M. van Haelst, The utility of obesity polygenic risk scores from research to clinical practice: A review. *Obes. Rev.* **25**, e13810 (2024). doi:10.1111/obr.13810 Medline

57. E. K. Karlsson, K. Lindblad-Toh, Leader of the pack: Gene mapping in dogs and other model organisms. *Nat. Rev. Genet.* **9**, 713–725 (2008). doi:10.1038/nrg2382 Medline

58. B. de Lauzon-Guillain, E. A. Clifton, F. R. Day, K. Clément, S. Brage, N. G. Forouhi, S. J. Griffin, Y. A. Koudou, V. Pelloux, N. J. Wareham, M.-A. Charles, B. Heude, K. K. Ong, Mediation and modification of genetic susceptibility to obesity by eating behaviors. *Am. J. Clin. Nutr.* **106**, 996–1004 (2017). doi:10.3945/ajcn.117.157396 Medline

59. C. H. Llewellyn, A. R. Kininmonth, M. Herle, Z. Nas, A. D. Smith, S. Carnell, A. Fildes, Behavioural susceptibility theory: The role of appetite in genetic susceptibility to obesity in early life. *Phil. Trans. R. Soc. B* **378**, 20220223 (2023). doi:10.1098/rstb.2022.0223 Medline

60. J. Yang, S. H. Lee, M. E. Goddard, P. M. Visscher, GCTA: A tool for genome-wide complex trait analysis. *Am. J. Hum. Genet.* **88**, 76–82 (2011). doi:10.1016/j.ajhg.2010.11.011 Medline

61. S. Purcell, B. Neale, K. Todd-Brown, L. Thomas, M. A. R. Ferreira, D. Bender, J. Maller, P. Sklar, P. I. W. de Bakker, M. J. Daly, P. C. Sham, PLINK: A tool set for whole-genome association and population-based linkage analyses. *Am. J. Hum. Genet.* **81**, 559–575 (2007). doi:10.1086/519795 Medline

62. R Core Team, R: A Language and Environment for Statistical Computing, version 4.2.2 (R Foundation for Statistical Computing, 2021).

63. J. Yang, A. Bakshi, Z. Zhu, G. Hemani, A. A. E. Vinkhuyzen, S. H. Lee, M. R. Robinson, J. R. B. Perry, I. M. Nolte, J. V. van Vliet-Ostaptchouk, H. Snieder, LifeLines Cohort

Study, T. Esko, L. Milani, R. Mägi, A. Metspalu, A. Hamsten, P. K. E. Magnusson, N. L. Pedersen, E. Ingelsson, N. Soranzo, M. C. Keller, N. R. Wray, M. E. Goddard, P. M. Visscher, Genetic variance estimation with imputed variants finds negligible missing heritability for human height and body mass index. *Nat. Genet.* **47**, 1114–1120 (2015). doi:10.1038/ng.3390 Medline

64. P.-R. Loh, G. Tucker, B. K. Bulik-Sullivan, B. J. Vilhjálmsson, H. K. Finucane, R. M. Salem, D. I. Chasman, P. M. Ridker, B. M. Neale, B. Berger, N. Patterson, A. L. Price, Efficient Bayesian mixed-model analysis increases association power in large cohorts. *Nat. Genet.* **47**, 284–290 (2015). doi:10.1038/ng.3190 Medline

65. H. Pan, T. Cole, LMSgrowth, a Microsoft Excel add-in to access growth references based on the LMS method, version 2.77 (2012); http://www.healthforallchildren.co.uk/.

66. WHO Multicentre Growth Reference Study Group, *WHO Child Growth Standards: Length/Height-for-Age, Weight-for-Age, Weight-for-Length, Weight-for-Height and Body Mass Index-for-Age: Methods and Development* (World Health Organization, 2006).

67. E. W. Sayers, E. E. Bolton, J. R. Brister, K. Canese, J. Chan, D. C. Comeau, R. Connor, K. Funk, C. Kelly, S. Kim, T. Madej, A. Marchler-Bauer, C. Lanczycki, S. Lathrop, Z. Lu, F. Thibaud-Nissen, T. Murphy, L. Phan, Y. Skripchenko, T. Tse, J. Wang, R. Williams, B. W. Trawick, K. D. Pruitt, S. T. Sherry, Database resources of the national center for biotechnology information. *Nucleic Acids Res.* **50**, D20–D26 (2022). doi:10.1093/nar/gkab1112 Medline

68. D. Taliun, D. N. Harris, M. D. Kessler, J. Carlson, Z. A. Szpiech, R. Torres, S. A. G. Taliun, A. Corvelo, S. M. Gogarten, H. M. Kang, A. N. Pitsillides, J. LeFaive, S.-B. Lee, X. Tian, B. L. Browning, S. Das, A.-K. Emde, W. E. Clarke, D. P. Loesch, A. C. Shetty, T. W. Blackwell, A. V. Smith, Q. Wong, X. Liu, M. P. Conomos, D. M. Bobo, F. Aguet, C. Albert, A. Alonso, K. G. Ardlie, D. E. Arking, S. Aslibekyan, P. L. Auer, J. Barnard, R. G. Barr, L. Barwick, L. C. Becker, R. L. Beer, E. J. Benjamin, L. F. Bielak, J. Blangero, M. Boehnke, D. W. Bowden, J. A. Brody, E. G. Burchard, B. E. Cade, J. F. Casella, B. Chalazan, D. I. Chasman, Y.-D. I. Chen, M. H. Cho, S. H. Choi, M. K. Chung, C. B. Clish, A. Correa, J. E. Curran, B. Custer, D. Darbar, M. Daya, M. de Andrade, D. L. DeMeo, S. K. Dutcher, P. T. Ellinor, L. S. Emery, C. Eng, D. Fatkin, T. Fingerlin, L. Forer, M. Fornage, N. Franceschini, C. Fuchsberger, S. M. Fullerton, S. Germer, M. T. Gladwin, D. J. Gottlieb, X. Guo, M. E. Hall, J. He, N. L. Heard-Costa, S. R. Heckbert, M. R. Irvin, J. M. Johnsen, A. D. Johnson, R. Kaplan, S. L. R. Kardia, T. Kelly, S. Kelly, E. E. Kenny, D. P. Kiel, R. Klemmer, B. A. Konkle, C. Kooperberg, A. Köttgen, L. A. Lange, J. Lasky-Su, D. Levy, X. Lin, K.-H. Lin, C. Liu, R. J. F. Loos, L. Garman, R. Gerszten, S. A. Lubitz, K. L. Lunetta, A. C. Y. Mak, A. Manichaikul, A. K. Manning, R. A. Mathias, D. D. McManus, S. T. McGarvey, J. B. Meigs, D. A. Meyers, J. L. Mikulla, M. A. Minear, B. D. Mitchell, S. Mohanty, M. E. Montasser, C. Montgomery, A. C. Morrison, J. M. Murabito, A. Natale, P. Natarajan, S. C. Nelson, K. E. North, J. R. O'Connell, N. D. Palmer, N. Pankratz, G. M. Peloso, P. A. Peyser, J. Pleiness, W. S. Post, B. M. Psaty, D. C. Rao, S. Redline, A. P. Reiner, D. Roden, J. I. Rotter, I. Ruczinski, C. Sarnowski, S. Schoenherr, D. A. Schwartz, J.-S. Seo, S. Seshadri, V. A. Sheehan, W. H. Sheu, M. B. Shoemaker, N. L. Smith, J. A. Smith, N. Sotoodehnia, A. M. Stilp, W. Tang, K. D. Taylor, M. Telen, T. A. Thornton, R. P. Tracy, D. J. Van Den Berg,

R. S. Vasan, K. A. Viaud-Martinez, S. Vrieze, D. E. Weeks, B. S. Weir, S. T. Weiss, L.-C. Weng, C. J. Willer, Y. Zhang, X. Zhao, D. K. Arnett, A. E. Ashley-Koch, K. C. Barnes, E. Boerwinkle, S. Gabriel, R. Gibbs, K. M. Rice, S. S. Rich, E. K. Silverman, P. Qasba, W. Gan, NHLBI Trans-Omics for Precision Medicine (TOPMed) Consortium, G. J. Papanicolaou, D. A. Nickerson, S. R. Browning, M. C. Zody, S. Zöllner, J. G. Wilson, L. A. Cupples, C. C. Laurie, C. E. Jaquish, R. D. Hernandez, T. D. O'Connor, G. R. Abecasis, Sequencing of 53,831 diverse genomes from the NHLBI TOPMed Program. *Nature* **590**, 290–299 (2021). doi:10.1038/s41586-021-03205-y Medline

69. M. J. Landrum, J. M. Lee, M. Benson, G. R. Brown, C. Chao, S. Chitipiralla, B. Gu, J. Hart, D. Hoffman, W. Jang, K. Karapetyan, K. Katz, C. Liu, Z. Maddipatla, A. Malheiro, K. McDaniel, M. Ovetsky, G. Riley, G. Zhou, J. B. Holmes, B. L. Kattman, D. R. Maglott, ClinVar: Improving access to variant interpretations and supporting evidence. *Nucleic Acids Res.* **46**, D1062–D1067 (2018). doi:10.1093/nar/gkx1153 Medline

70. A. E. Adriaenssens, E. K. Biggs, T. Darwish, J. Tadross, T. Sukthankar, M. Girish, J. Polex-Wolf, B. Y. Lam, I. Zvetkova, W. Pan, D. Chiarugi, G. S. H. Yeo, C. Blouet, F. M. Gribble, F. Reimann, Glucose-Dependent Insulinotropic Polypeptide Receptor-Expressing Cells in the Hypothalamus Regulate Food Intake. *Cell Metab.* **30**, 987–996.e6 (2019). doi:10.1016/j.cmet.2019.07.013 Medline

71. H. A. Abid, A. Inoue, C. M. Gorvin, Heterogeneity of G protein activation by the calcium-sensing receptor. *J. Mol. Endocrinol.* **67**, 41–53 (2021). doi:10.1530/JME-21-0058 Medline

72. S. Bolte, F. P. Cordelières, A guided tour into subcellular colocalization analysis in light microscopy. *J. Microsc.* **224**, 213–232 (2006). doi:10.1111/j.1365-2818.2006.01706.x Medline

73. N. J. Wallis, A. McClellan, A. Mörseburg, K. A. Kentistou, A. Jamaluddin, G. K. C. Dowsett, E. Schofield, A. Morros-Nuevo, S. Saeed, B. Y. H. Lam, N. T. Sumanasekera, J. Chan, S. S. Kumar, R. M. Zhang, J. F. Wainwright, M. Dittmann, G. Lakatos, K. Rainbow, D. Withers, R. Bounds, M. Ma, A. J. German, J. Ladlow, D. Sargan, P. Froguel, I. S. Farooqi, K. K. Ong, G. S. H. Yeo, J. A. Tadross, J. R. B. Perry, C. M. Gorvin, E. Raffan, Canine genome-wide association study identifies DENND1B as an obesity gene in dogs and humans, Dryad (2024). doi:10.5061/dryad.0vt4b8h85

74. E. Schofield, GOdogs-Project/Imputation: Version 0.1.1, version-0.1.1, Zenodo (2024). doi:10.5281/zenodo.14541383

75. J. O'Connell, D. Gurdasani, O. Delaneau, N. Pirastu, S. Ulivi, M. Cocca, M. Traglia, J. Huang, J. E. Huffman, I. Rudan, R. McQuillan, R. M. Fraser, H. Campbell, O. Polasek, G. Asiki, K. Ekoru, C. Hayward, A. F. Wright, V. Vitart, P. Navarro, J.-F. Zagury, J. F. Wilson, D. Toniolo, P. Gasparini, N. Soranzo, M. S. Sandhu, J. Marchini, A general approach for haplotype phasing across the full spectrum of relatedness. *PLOS Genet.* **10**, e1004234 (2014). doi:10.1371/journal.pgen.1004234 Medline

76. R. M. Buckley, A. C. Harris, G.-D. Wang, D. T. Whitaker, Y.-P. Zhang, E. A. Ostrander, Best practices for analyzing imputed genotypes from low-pass sequencing in dogs. *Mamm. Genome* **33**, 213–229 (2022). doi:10.1007/s00335-021-09914-z Medline

77. A. R. Quinlan, I. M. Hall, BEDTools: A flexible suite of utilities for comparing genomic features. *Bioinformatics* **26**, 841–842 (2010). doi:10.1093/bioinformatics/btq033 Medline

78. P. Danecek, J. K. Bonfield, J. Liddle, J. Marshall, V. Ohan, M. O. Pollard, A. Whitwham, T. Keane, S. A. McCarthy, R. M. Davies, H. Li, Twelve years of SAMtools and BCFtools. *Gigascience* **10**, giab008 (2021). doi:10.1093/gigascience/giab008 Medline

79. B. N. Howie, P. Donnelly, J. Marchini, A flexible and accurate genotype imputation method for the next generation of genome-wide association studies. *PLOS Genet.* **5**, e1000529 (2009). doi:10.1371/journal.pgen.1000529 Medline

80. J. H. Li, C. A. Mazur, T. Berisa, J. K. Pickrell, Low-pass sequencing increases the power of GWAS and decreases measurement error of polygenic risk scores compared to genotyping arrays. *Genome Res.* **31**, 529–537 (2021). doi:10.1101/gr.266486.120 Medline

81. K. Morrill, J. Hekman, X. Li, J. McClure, B. Logan, L. Goodman, M. Gao, Y. Dong, M. Alonso, E. Carmichael, N. Snyder-Mackler, J. Alonso, H. J. Noh, J. Johnson, M. Koltookian, C. Lieu, K. Megquier, R. Swofford, J. Turner-Maier, M. E. White, Z. Weng, A. Colubri, D. P. Genereux, K. A. Lord, E. K. Karlsson, Ancestry-inclusive dog genomics challenges popular breed stereotypes. *Science* **376**, eabk0639 (2022). doi:10.1126/science.abk0639 Medline

82. J. Labadie, B. Swafford, M. DePena, K. Tietje, R. Page, J. Patterson-Kane, Cohort profile: The Golden Retriever Lifetime Study (GRLS). *PLOS ONE* **17**, e0269425 (2022). doi:10.1371/journal.pone.0269425 Medline

83. X. Zhou, M. Stephens, Genome-wide efficient mixed-model analysis for association studies. *Nat. Genet.* **44**, 821–824 (2012). doi:10.1038/ng.2310 Medline

84. F. J. Martin, M. R. Amode, A. Aneja, O. Austine-Orimoloye, A. G. Azov, I. Barnes, A. Becker, R. Bennett, A. Berry, J. Bhai, S. K. Bhurji, A. Bignell, S. Boddu, P. R. Branco Lins, L. Brooks, S. B. Ramaraju, M. Charkhchi, A. Cockburn, L. Da Rin Fiorretto, C. Davidson, K. Dodiya, S. Donaldson, B. El Houdaigui, T. El Naboulsi, R. Fatima, C. G. Giron, T. Genez, G. S. Ghattaoraya, J. G. Martinez, C. Guijarro, M. Hardy, Z. Hollis, T. Hourlier, T. Hunt, M. Kay, V. Kaykala, T. Le, D. Lemos, D. Marques-Coelho, J. C. Marugán, G. A. Merino, L. P. Mirabueno, A. Mushtaq, S. N. Hossain, D. N. Ogeh, M. P. Sakthivel, A. Parker, M. Perry, I. Piližota, I. Prosovetskaia, J. G. Pérez-Silva, A. I. A. Salam, N. Saraiva-Agostinho, H. Schuilenburg, D. Sheppard, S. Sinha, B. Sipos, W. Stark, E. Steed, R. Sukumaran, D. Sumathipala, M.-M. Suner, L. Surapaneni, K. Sutinen, M. Szpak, F. F. Tricomi, D. Urbina-Gómez, A. Veidenberg, T. A. Walsh, B. Walts, E. Wass, N. Willhoft, J. Allen, J. Alvarez-Jarreta, M. Chakiachvili, B. Flint, S. Giorgetti, L. Haggerty, G. R. Ilsley, J. E. Loveland, B. Moore, J. M. Mudge, J. Tate, D. Thybert, S. J. Trevanion, A. Winterbottom, A. Frankish, S. E. Hunt, M. Ruffier, F. Cunningham, S. Dyer, R. D. Finn, K. L. Howe, P. W. Harrison, A. D. Yates, P. Flicek, Ensembl 2023. *Nucleic Acids Res.* **51**, D933–D941 (2023). doi:10.1093/nar/gkac958 Medline

85. R. J. Kinsella, A. Kähäri, S. Haider, J. Zamora, G. Proctor, G. Spudich, J. Almeida-King, D. Staines, P. Derwent, A. Kerhornou, P. Kersey, P. Flicek, Ensembl BioMarts: A hub for data retrieval across taxonomic space. *Database* **2011**, bar030 (2011). doi:10.1093/database/bar030 Medline

86. W. J. Kent, C. W. Sugnet, T. S. Furey, K. M. Roskin, T. H. Pringle, A. M. Zahler, D. Haussler, The human genome browser at UCSC. *Genome Res.* **12**, 996–1006 (2002). doi:10.1101/gr.229102 Medline

87. H. Thorvaldsdóttir, J. T. Robinson, J. P. Mesirov, Integrative Genomics Viewer (IGV): High-performance genomics data visualization and exploration. *Brief. Bioinform.* **14**, 178–192 (2013). doi:10.1093/bib/bbs017 Medline

88. W. McLaren, L. Gil, S. E. Hunt, H. S. Riat, G. R. S. Ritchie, A. Thormann, P. Flicek, F. Cunningham, The Ensembl Variant Effect Predictor. *Genome Biol.* **17**, 122 (2016). doi:10.1186/s13059-016-0974-4 Medline

89. S. B. Gabriel, S. F. Schaffner, H. Nguyen, J. M. Moore, J. Roy, B. Blumenstiel, J. Higgins, M. DeFelice, A. Lochner, M. Faggart, S. N. Liu-Cordero, C. Rotimi, A. Adeyemo, R. Cooper, R. Ward, E. S. Lander, M. J. Daly, D. Altshuler, The structure of haplotype blocks in the human genome. *Science* **296**, 2225–2229 (2002). doi:10.1126/science.1069424 Medline

90. D. Szklarczyk, R. Kirsch, M. Koutrouli, K. Nastou, F. Mehryary, R. Hachilif, A. L. Gable, T. Fang, N. T. Doncheva, S. Pyysalo, P. Bork, L. J. Jensen, C. von Mering, The STRING database in 2023: Protein-protein association networks and functional enrichment analyses for any sequenced genome of interest. *Nucleic Acids Res.* **51**, D638–D646 (2023). doi:10.1093/nar/gkac1000 Medline

91. P. D. Thomas, D. Ebert, A. Muruganujan, T. Mushayahama, L.-P. Albou, H. Mi, PANTHER: Making genome-scale phylogenetics accessible to all. *Protein Sci.* **31**, 8–22 (2022). doi:10.1002/pro.4218 Medline

92. T. Groza, F. L. Gomez, H. H. Mashhadi, V. Muñoz-Fuentes, O. Gunes, R. Wilson, P. Cacheiro, A. Frost, P. Keskivali-Bond, B. Vardal, A. McCoy, T. K. Cheng, L. Santos, S. Wells, D. Smedley, A.-M. Mallon, H. Parkinson, The International Mouse Phenotyping Consortium: Comprehensive knockout phenotyping underpinning the study of human disease. *Nucleic Acids Res.* **51**, D1038–D1045 (2023). doi:10.1093/nar/gkac972 Medline

93. J. A. Blake, R. Baldarelli, J. A. Kadin, J. E. Richardson, C. L. Smith, C. J. Bult, Mouse Genome Database Group, Mouse Genome Database (MGD): Knowledgebase for mouse-human comparative biology. *Nucleic Acids Res.* **49**, D981–D987 (2021). doi:10.1093/nar/gkaa1083 Medline

94. J. MacArthur, E. Bowler, M. Cerezo, L. Gil, P. Hall, E. Hastings, H. Junkins, A. McMahon, A. Milano, J. Morales, Z. M. Pendlington, D. Welter, T. Burdett, L. Hindorff, P. Flicek, F. Cunningham, H. Parkinson, The new NHGRI-EBI Catalog of published genome-wide association studies (GWAS Catalog). *Nucleic Acids Res.* **45**, D896–D901 (2017). doi:10.1093/nar/gkw1133 Medline

95. K. Watanabe, S. Stringer, O. Frei, M. Umićević Mirkov, C. de Leeuw, T. J. C. Polderman, S. van der Sluis, O. A. Andreassen, B. M. Neale, D. Posthuma, A global overview of pleiotropy and genetic architecture in complex traits. *Nat. Genet.* **51**, 1339–1348 (2019). doi:10.1038/s41588-019-0481-0 Medline

96. K. J. Karczewski, M. Solomonson, K. R. Chao, J. K. Goodrich, G. Tiao, W. Lu, B. M. Riley-Gillis, E. A. Tsai, H. I. Kim, X. Zheng, F. Rahimov, S. Esmaeeli, A. J. Grundstad, M.

Reppell, J. Waring, H. Jacob, D. Sexton, P. G. Bronson, X. Chen, X. Hu, J. I. Goldstein, D. King, C. Vittal, T. Poterba, D. S. Palmer, C. Churchhouse, D. P. Howrigan, W. Zhou, N. A. Watts, K. Nguyen, H. Nguyen, C. Mason, C. Farnham, C. Tolonen, L. D. Gauthier, N. Gupta, D. G. MacArthur, H. L. Rehm, C. Seed, A. A. Philippakis, M. J. Daly, J. W. Davis, H. Runz, M. R. Miller, B. M. Neale, Systematic single-variant and gene-based association testing of thousands of phenotypes in 394,841 UK Biobank exomes. *Cell Genom.* **2**, 100168 (2022). doi:10.1016/j.xgen.2022.100168 Medline

97. A. Thormann, M. Halachev, W. McLaren, D. J. Moore, V. Svinti, A. Campbell, S. M. Kerr, M. Tischkowitz, S. E. Hunt, M. G. Dunlop, M. E. Hurles, C. F. Wright, H. V. Firth, F. Cunningham, D. R. FitzPatrick, Flexible and scalable diagnostic filtering of genomic variants using G2P with Ensembl VEP. *Nat. Commun.* **10**, 2373 (2019). doi:10.1038/s41467-019-10016-3 Medline

98. M. Uhlén, L. Fagerberg, B. M. Hallström, C. Lindskog, P. Oksvold, A. Mardinoglu, Å. Sivertsson, C. Kampf, E. Sjöstedt, A. Asplund, I. Olsson, K. Edlund, E. Lundberg, S. Navani, C. A.-K. Szigyarto, J. Odeberg, D. Djureinovic, J. O. Takanen, S. Hober, T. Alm, P.-H. Edqvist, H. Berling, H. Tegel, J. Mulder, J. Rockberg, P. Nilsson, J. M. Schwenk, M. Hamsten, K. von Feilitzen, M. Forsberg, L. Persson, F. Johansson, M. Zwahlen, G. von Heijne, J. Nielsen, F. Pontén, Tissue-based map of the human proteome. *Science* **347**, 1260419 (2015). doi:10.1126/science.1260419 Medline

99. J. Lonsdale, J. Thomas, M. Salvatore, R. Phillips, E. Lo, S. Shad, R. Hasz, G. Walters, F. Garcia, N. Young, B. Foster, M. Moser, E. Karasik, B. Gillard, K. Ramsey, S. Sullivan, J. Bridge, H. Magazine, J. Syron, J. Fleming, L. Siminoff, H. Traino, M. Mosavel, L. Barker, S. Jewell, D. Rohrer, D. Maxim, D. Filkins, P. Harbach, E. Cortadillo, B. Berghuis, L. Turner, E. Hudson, K. Feenstra, L. Sobin, J. Robb, P. Branton, G. Korzeniewski, C. Shive, D. Tabor, L. Qi, K. Groch, S. Nampally, S. Buia, A. Zimmerman, A. Smith, R. Burges, K. Robinson, K. Valentino, D. Bradbury, M. Cosentino, N. Diaz-Mayoral, M. Kennedy, T. Engel, P. Williams, K. Erickson, K. Ardlie, W. Winckler, G. Getz, D. DeLuca, D. MacArthur, M. Kellis, A. Thomson, T. Young, E. Gelfand, M. Donovan, Y. Meng, G. Grant, D. Mash, Y. Marcus, M. Basile, J. Liu, J. Zhu, Z. Tu, N. J. Cox, D. L. Nicolae, E. R. Gamazon, H. K. Im, A. Konkashbaev, J. Pritchard, M. Stevens, T. Flutre, X. Wen, E. T. Dermitzakis, T. Lappalainen, R. Guigo, J. Monlong, M. Sammeth, D. Koller, A. Battle, S. Mostafavi, M. McCarthy, M. Rivas, J. Maller, I. Rusyn, A. Nobel, F. Wright, A. Shabalin, M. Feolo, N. Sharopova, A. Sturcke, J. Paschal, J. M. Anderson, E. L. Wilder, L. K. Derr, E. D. Green, J. P. Struewing, G. Temple, S. Volpi, J. T. Boyer, E. J. Thomson, M. S. Guyer, C. Ng, A. Abdallah, D. Colantuoni, T. R. Insel, S. E. Koester, A. R. Little, P. K. Bender, T. Lehner, Y. Yao, C. C. Compton, J. B. Vaught, S. Sawyer, N. C. Lockhart, J. Demchok, H. F. Moore, The Genotype-Tissue Expression (GTEx) project. *Nat. Genet.* **45**, 580–585 (2013). doi:10.1038/ng.2653 Medline

100. R. M. Baldarelli, C. M. Smith, J. H. Finger, T. F. Hayamizu, I. J. McCright, J. Xu, D. R. Shaw, J. S. Beal, O. Blodgett, J. Campbell, L. E. Corbani, P. J. Frost, S. C. Giannatto, D. B. Miers, J. A. Kadin, J. E. Richardson, M. Ringwald, The mouse Gene Expression Database (GXD): 2021 update. *Nucleic Acids Res.* **49**, D924–D931 (2021). doi:10.1093/nar/gkaa914 Medline

101. Y. Hao, S. Hao, E. Andersen-Nissen, W. M. Mauck 3rd, S. Zheng, A. Butler, M. J. Lee, A. J. Wilk, C. Darby, M. Zager, P. Hoffman, M. Stoeckius, E. Papalexi, E. P. Mimitou, J. Jain, A. Srivastava, T. Stuart, L. M. Fleming, B. Yeung, A. J. Rogers, J. M. McElrath, C. A. Blish, R. Gottardo, P. Smibert, R. Satija, Integrated analysis of multimodal single-cell data. *Cell* **184**, 3573–3587.e29 (2021). doi:10.1016/j.cell.2021.04.048 Medline

102. W. K. Kroeze, M. F. Sassano, X.-P. Huang, K. Lansu, J. D. McCorvy, P. M. Giguère, N. Sciaky, B. L. Roth, PRESTO-Tango as an open-source resource for interrogation of the druggable human GPCRome. *Nat. Struct. Mol. Biol.* **22**, 362–369 (2015). doi:10.1038/nsmb.3014 Medline

103. K. J. Livak, T. D. Schmittgen, Analysis of relative gene expression data using real-time quantitative PCR and the $2^{-\Delta\Delta C_T}$ Method. *Methods* **25**, 402–408 (2001). doi:10.1006/meth.2001.1262 Medline

104. S. L. Pulit, C. Stoneman, A. P. Morris, A. R. Wood, C. A. Glastonbury, J. Tyrrell, L. Yengo, T. Ferreira, E. Marouli, Y. Ji, J. Yang, S. Jones, R. Beaumont, D. C. Croteau-Chonka, T. W. Winkler, GIANT Consortium, A. T. Hattersley, R. J. F. Loos, J. N. Hirschhorn, P. M. Visscher, T. M. Frayling, H. Yaghootkar, C. M. Lindgren, Meta-analysis of genome-wide association studies for body fat distribution in 694 649 individuals of European ancestry. *Hum. Mol. Genet.* **28**, 166–174 (2019). doi:10.1093/hmg/ddy327 Medline

105. S. E. Graham, S. L. Clarke, K.-H. H. Wu, S. Kanoni, G. J. M. Zajac, S. Ramdas, I. Surakka, I. Ntalla, S. Vedantam, T. W. Winkler, A. E. Locke, E. Marouli, M. Y. Hwang, S. Han, A. Narita, A. Choudhury, A. R. Bentley, K. Ekoru, A. Verma, B. Trivedi, H. C. Martin, K. A. Hunt, Q. Hui, D. Klarin, X. Zhu, G. Thorleifsson, A. Helgadottir, D. F. Gudbjartsson, H. Holm, I. Olafsson, M. Akiyama, S. Sakaue, C. Terao, M. Kanai, W. Zhou, B. M. Brumpton, H. Rasheed, S. E. Ruotsalainen, A. S. Havulinna, Y. Veturi, Q. Feng, E. A. Rosenthal, T. Lingren, J. A. Pacheco, S. A. Pendergrass, J. Haessler, F. Giulianini, Y. Bradford, J. E. Miller, A. Campbell, K. Lin, I. Y. Millwood, G. Hindy, A. Rasheed, J. D. Faul, W. Zhao, D. R. Weir, C. Turman, H. Huang, M. Graff, A. Mahajan, M. R. Brown, W. Zhang, K. Yu, E. M. Schmidt, A. Pandit, S. Gustafsson, X. Yin, J. Luan, J.-H. Zhao, F. Matsuda, H.-M. Jang, K. Yoon, C. Medina-Gomez, A. Pitsillides, J. J. Hottenga, G. Willemsen, A. R. Wood, Y. Ji, Z. Gao, S. Haworth, R. E. Mitchell, J. F. Chai, M. Aadahl, J. Yao, A. Manichaikul, H. R. Warren, J. Ramirez, J. Bork-Jensen, L. L. Kårhus, A. Goel, M. Sabater-Lleal, R. Noordam, C. Sidore, E. Fiorillo, A. F. McDaid, P. Marques-Vidal, M. Wielscher, S. Trompet, N. Sattar, L. T. Møllehave, B. H. Thuesen, M. Munz, L. Zeng, J. Huang, B. Yang, A. Poveda, A. Kurbasic, C. Lamina, L. Forer, M. Scholz, T. E. Galesloot, J. P. Bradfield, E. W. Daw, J. M. Zmuda, J. S. Mitchell, C. Fuchsberger, H. Christensen, J. A. Brody, M. F. Feitosa, M. K. Wojczynski, M. Preuss, M. Mangino, P. Christofidou, N. Verweij, J. W. Benjamins, J. Engmann, R. L. Kember, R. C. Slieker, K. S. Lo, N. R. Zilhao, P. Le, M. E. Kleber, G. E. Delgado, S. Huo, D. D. Ikeda, H. Iha, J. Yang, J. Liu, H. L. Leonard, J. Marten, B. Schmidt, M. Arendt, L. J. Smyth, M. Cañadas-Garre, C. Wang, M. Nakatochi, A. Wong, N. Hutri-Kähönen, X. Sim, R. Xia, A. Huerta-Chagoya, J. C. Fernandez-Lopez, V. Lyssenko, M. Ahmed, A. U. Jackson, N. A. Yousri, M. R. Irvin, C. Oldmeadow, H.-N. Kim, S. Ryu, P. R. H. J. Timmers, L. Arbeeva, R. Dorajoo, L. A. Lange, X. Chai, G. Prasad, L. Lorés-Motta, M. Pauper, J. Long, X. Li, E. Theusch, F. Takeuchi, C. N. Spracklen, A. Loukola, S.

Bollepalli, S. C. Warner, Y. X. Wang, W. B. Wei, T. Nutile, D. Ruggiero, Y. J. Sung, Y.-J. Hung, S. Chen, F. Liu, J. Yang, K. A. Kentistou, M. Gorski, M. Brumat, K. Meidtner, L. F. Bielak, J. A. Smith, P. Hebbar, A.-E. Farmaki, E. Hofer, M. Lin, C. Xue, J. Zhang, M. P. Concas, S. Vaccargiu, P. J. van der Most, N. Pitkänen, B. E. Cade, J. Lee, S. W. van der Laan, K. N. Chitrala, S. Weiss, M. E. Zimmermann, J. Y. Lee, H. S. Choi, M. Nethander, S. Freitag-Wolf, L. Southam, N. W. Rayner, C. A. Wang, S.-Y. Lin, J.-S. Wang, C. Couture, L.-P. Lyytikäinen, K. Nikus, G. Cuellar-Partida, H. Vestergaard, B. Hildalgo, O. Giannakopoulou, Q. Cai, M. O. Obura, J. van Setten, X. Li, K. Schwander, N. Terzikhan, J. H. Shin, R. D. Jackson, A. P. Reiner, L. W. Martin, Z. Chen, L. Li, H. M. Highland, K. L. Young, T. Kawaguchi, J. Thiery, J. C. Bis, G. N. Nadkarni, L. J. Launer, H. Li, M. A. Nalls, O. T. Raitakari, S. Ichihara, S. H. Wild, C. P. Nelson, H. Campbell, S. Jäger, T. Nabika, F. Al-Mulla, H. Niinikoski, P. S. Braund, I. Kolcic, P. Kovacs, T. Giardoglou, T. Katsuya, K. F. Bhatti, D. de Kleijn, G. J. de Borst, E. K. Kim, H. H. H. Adams, M. A. Ikram, X. Zhu, F. W. Asselbergs, A. O. Kraaijeveld, J. W. J. Beulens, X.-O. Shu, L. S. Rallidis, O. Pedersen, T. Hansen, P. Mitchell, A. W. Hewitt, M. Kähönen, L. Pérusse, C. Bouchard, A. Tönjes, Y. I. Chen, C. E. Pennell, T. A. Mori, W. Lieb, A. Franke, C. Ohlsson, D. Mellström, Y. S. Cho, H. Lee, J.-M. Yuan, W.-P. Koh, S. Y. Rhee, J.-T. Woo, I. M. Heid, K. J. Stark, H. Völzke, G. Homuth, M. K. Evans, A. B. Zonderman, O. Polasek, G. Pasterkamp, I. E. Hoefer, S. Redline, K. Pahkala, A. J. Oldehinkel, H. Snieder, G. Biino, R. Schmidt, H. Schmidt, Y. E. Chen, S. Bandinelli, G. Dedoussis, T. A. Thanaraj, S. L. R. Kardia, N. Kato, M. B. Schulze, G. Girotto, B. Jung, C. A. Böger, P. K. Joshi, D. A. Bennett, P. L. De Jager, X. Lu, V. Mamakou, M. Brown, M. J. Caulfield, P. B. Munroe, X. Guo, M. Ciullo, J. B. Jonas, N. J. Samani, J. Kaprio, P. Pajukanta, L. S. Adair, S. A. Bechayda, H. J. de Silva, A. R. Wickremasinghe, R. M. Krauss, J.-Y. Wu, W. Zheng, A. I. den Hollander, D. Bharadwaj, A. Correa, J. G. Wilson, L. Lind, C.-K. Heng, A. E. Nelson, Y. M. Golightly, J. F. Wilson, B. Penninx, H.-L. Kim, J. Attia, R. J. Scott, D. C. Rao, D. K. Arnett, S. C. Hunt, M. Walker, H. A. Koistinen, G. R. Chandak, C. S. Yajnik, J. M. Mercader, T. Tusié-Luna, C. A. Aguilar-Salinas, C. G. Villalpando, L. Orozco, M. Fornage, E. S. Tai, R. M. van Dam, T. Lehtimäki, N. Chaturvedi, M. Yokota, J. Liu, D. F. Reilly, A. J. McKnight, F. Kee, K.-H. Jöckel, M. I. McCarthy, C. N. A. Palmer, V. Vitart, C. Hayward, E. Simonsick, C. M. van Duijn, F. Lu, J. Qu, H. Hishigaki, X. Lin, W. März, E. J. Parra, M. Cruz, V. Gudnason, J.-C. Tardif, G. Lettre, L. M. ’t Hart, P. J. M. Elders, S. M. Damrauer, M. Kumari, M. Kivimaki, P. van der Harst, T. D. Spector, R. J. F. Loos, M. A. Province, B. M. Psaty, I. Brandslund, P. P. Pramstaller, K. Christensen, S. Ripatti, E. Widén, H. Hakonarson, S. F. A. Grant, L. A. L. M. Kiemeney, J. de Graaf, M. Loeffler, F. Kronenberg, D. Gu, J. Erdmann, H. Schunkert, P. W. Franks, A. Linneberg, J. W. Jukema, A. V. Khera, M. Männikkö, M.-R. Jarvelin, Z. Kutalik, F. Cucca, D. O. Mook-Kanamori, K. W. van Dijk, H. Watkins, D. P. Strachan, N. Grarup, P. Sever, N. Poulter, J. I. Rotter, T. M. Dantoft, F. Karpe, M. J. Neville, N. J. Timpson, C.-Y. Cheng, T.-Y. Wong, C. C. Khor, C. Sabanayagam, A. Peters, C. Gieger, A. T. Hattersley, N. L. Pedersen, P. K. E. Magnusson, D. I. Boomsma, E. J. C. de Geus, L. A. Cupples, J. B. J. van Meurs, M. Ghanbari, P. Gordon-Larsen, W. Huang, Y. J. Kim, Y. Tabara, N. J. Wareham, C. Langenberg, E. Zeggini, J. Kuusisto, M. Laakso, E. Ingelsson, G. Abecasis, J. C. Chambers, J. S. Kooner, P. S. de Vries, A. C. Morrison, K. E. North, M. Daviglus, P. Kraft, N. G. Martin, J. B. Whitfield, S. Abbas, D. Saleheen, R. G. Walters, M. V.

Holmes, C. Black, B. H. Smith, A. E. Justice, A. Baras, J. E. Buring, P. M. Ridker, D. I. Chasman, C. Kooperberg, W.-Q. Wei, G. P. Jarvik, B. Namjou, M. G. Hayes, M. D. Ritchie, P. Jousilahti, V. Salomaa, K. Hveem, B. O. Åsvold, M. Kubo, Y. Kamatani, Y. Okada, Y. Murakami, U. Thorsteinsdottir, K. Stefansson, Y.-L. Ho, J. A. Lynch, D. J. Rader, P. S. Tsao, K.-M. Chang, K. Cho, C. J. O'Donnell, J. M. Gaziano, P. Wilson, C. N. Rotimi, S. Hazelhurst, M. Ramsay, R. C. Trembath, D. A. van Heel, G. Tamiya, M. Yamamoto, B.-J. Kim, K. L. Mohlke, T. M. Frayling, J. N. Hirschhorn, S. Kathiresan, VA Million Veteran Program, Global Lipids Genetics Consortium, M. Boehnke, P. Natarajan, G. M. Peloso, C. D. Brown, A. P. Morris, T. L. Assimes, P. Deloukas, Y. V. Sun, C. J. Willer, The power of genetic diversity in genome-wide association studies of lipids. *Nature* **600**, 675–679 (2021). doi:10.1038/s41586-021-04064-3 Medline

106. C. A. de Leeuw, J. M. Mooij, T. Heskes, D. Posthuma, MAGMA: Generalized gene-set analysis of GWAS data. *PLOS Comput. Biol.* **11**, e1004219 (2015). doi:10.1371/journal.pcbi.1004219 Medline

107. Z. Zhu, F. Zhang, H. Hu, A. Bakshi, M. R. Robinson, J. E. Powell, G. W. Montgomery, M. E. Goddard, N. R. Wray, P. M. Visscher, J. Yang, Integration of summary data from GWAS and eQTL studies predicts complex trait gene targets. *Nat. Genet.* **48**, 481–487 (2016). doi:10.1038/ng.3538 Medline

108. C. Giambartolomei, D. Vukcevic, E. E. Schadt, L. Franke, A. D. Hingorani, C. Wallace, V. Plagnol, Bayesian test for colocalisation between pairs of genetic association studies using summary statistics. *PLOS Genet.* **10**, e1004383 (2014). doi:10.1371/journal.pgen.1004383 Medline

109. H. K. Finucane, Y. A. Reshef, V. Anttila, K. Slowikowski, A. Gusev, A. Byrnes, S. Gazal, P.-R. Loh, C. Lareau, N. Shoresh, G. Genovese, A. Saunders, E. Macosko, S. Pollack, Brainstorm Consortium, J. R. B. Perry, J. D. Buenrostro, B. E. Bernstein, S. Raychaudhuri, S. McCarroll, B. M. Neale, A. L. Price, Heritability enrichment of specifically expressed genes identifies disease-relevant tissues and cell types. *Nat. Genet.* **50**, 621–629 (2018). doi:10.1038/s41588-018-0081-4 Medline

110. GTEx Consortium, K. G. Ardlie, D. S. Deluca, A. V. Segrè, T. J. Sullivan, T. R. Young, E. T. Gelfand, C. A. Trowbridge, J. B. Maller, T. Tukiainen, M. Lek, L. D. Ward, P. Kheradpour, B. Iriarte, Y. Meng, C. D. Palmer, T. Esko, W. Winckler, J. N. Hirschhorn, M. Kellis, D. G. MacArthur, G. Getz, A. A. Shabalin, G. Li, Y.-H. Zhou, A. B. Nobel, I. Rusyn, F. A. Wright, T. Lappalainen, P. G. Ferreira, H. Ongen, M. A. Rivas, A. Battle, S. Mostafavi, J. Monlong, M. Sammeth, M. Mele, F. Reverter, J. M. Goldmann, D. Koller, R. Guigó, M. I. McCarthy, E. T. Dermitzakis, E. R. Gamazon, H. K. Im, A. Konkashbaev, D. L. Nicolae, N. J. Cox, T. Flutre, X. Wen, M. Stephens, J. K. Pritchard, Z. Tu, B. Zhang, T. Huang, Q. Long, L. Lin, J. Yang, J. Zhu, J. Liu, A. Brown, B. Mestichelli, D. Tidwell, E. Lo, M. Salvatore, S. Shad, J. A. Thomas, J. T. Lonsdale, M. T. Moser, B. M. Gillard, E. Karasik, K. Ramsey, C. Choi, B. A. Foster, J. Syron, J. Fleming, H. Magazine, R. Hasz, G. D. Walters, J. P. Bridge, M. Miklos, S. Sullivan, L. K. Barker, H. M. Traino, M. Mosavel, L. A. Siminoff, D. R. Valley, D. C. Rohrer, S. D. Jewell, P. A. Branton, L. H. Sobin, M. Barcus, L. Qi, J. McLean, P. Hariharan, K. S. Um, S. Wu, D. Tabor, C. Shive, A. M. Smith, S. A. Buia, A. H. Undale, K. L. Robinson, N. Roche, K. M. Valentino, A. Britton, R. Burges, D. Bradbury, K. W. Hambright, J.

Seleski, G. E. Korzeniewski, K. Erickson, Y. Marcus, J. Tejada, M. Taherian, C. Lu, M. Basile, D. C. Mash, S. Volpi, J. P. Struewing, G. F. Temple, J. Boyer, D. Colantuoni, R. Little, S. Koester, L. J. Carithers, H. M. Moore, P. Guan, C. Compton, S. J. Sawyer, J. P. Demchok, J. B. Vaught, C. A. Rabiner, N. C. Lockhart, K. G. Ardlie, G. Getz, F. A. Wright, M. Kellis, S. Volpi, E. T. Dermitzakis, The Genotype-Tissue Expression (GTEx) pilot analysis: Multitissue gene regulation in humans. *Science* **348**, 648–660 (2015). doi:10.1126/science.1262110 Medline

111. U. Võsa, A. Claringbould, H.-J. Westra, M. J. Bonder, P. Deelen, B. Zeng, H. Kirsten, A. Saha, R. Kreuzhuber, S. Yazar, H. Brugge, R. Oelen, D. H. de Vries, M. G. P. van der Wijst, S. Kasela, N. Pervjakova, I. Alves, M.-J. Favé, M. Agbessi, M. W. Christiansen, R. Jansen, I. Seppälä, L. Tong, A. Teumer, K. Schramm, G. Hemani, J. Verlouw, H. Yaghootkar, R. Sönmez Flitman, A. Brown, V. Kukushkina, A. Kalnapenkis, S. Rüeger, E. Porcu, J. Kronberg, J. Kettunen, B. Lee, F. Zhang, T. Qi, J. A. Hernandez, W. Arindrarto, F. Beutner, BIOS Consortium, i2QTL Consortium, J. Dmitrieva, M. Elansary, B. P. Fairfax, M. Georges, B. T. Heijmans, A. W. Hewitt, M. Kähönen, Y. Kim, J. C. Knight, P. Kovacs, K. Krohn, S. Li, M. Loeffler, U. M. Marigorta, H. Mei, Y. Momozawa, M. Müller-Nurasyid, M. Nauck, M. G. Nivard, B. W. J. H. Penninx, J. K. Pritchard, O. T. Raitakari, O. Rotzschke, E. P. Slagboom, C. D. A. Stehouwer, M. Stumvoll, P. Sullivan, P. A. C. 't Hoen, J. Thiery, A. Tönjes, J. van Dongen, M. van Iterson, J. H. Veldink, U. Völker, R. Warmerdam, C. Wijmenga, M. Swertz, A. Andiappan, G. W. Montgomery, S. Ripatti, M. Perola, Z. Kutalik, E. Dermitzakis, S. Bergmann, T. Frayling, J. van Meurs, H. Prokisch, H. Ahsan, B. L. Pierce, T. Lehtimäki, D. I. Boomsma, B. M. Psaty, S. A. Gharib, P. Awadalla, L. Milani, W. H. Ouwehand, K. Downes, O. Stegle, A. Battle, P. M. Visscher, J. Yang, M. Scholz, J. Powell, G. Gibson, T. Esko, L. Franke, Large-scale *cis*- and *trans*-eQTL analyses identify thousands of genetic loci and polygenic scores that regulate blood gene expression. *Nat. Genet.* **53**, 1300–1310 (2021). doi:10.1038/s41588-021-00913-z Medline

112. T. Qi, Y. Wu, J. Zeng, F. Zhang, A. Xue, L. Jiang, Z. Zhu, K. Kemper, L. Yengo, Z. Zheng, eQTLGen Consortium, R. E. Marioni, G. W. Montgomery, I. J. Deary, N. R. Wray, P. M. Visscher, A. F. McRae, J. Yang, Identifying gene targets for brain-related traits using transcriptomic and methylomic data from blood. *Nat. Commun.* **9**, 2282 (2018). doi:10.1038/s41467-018-04558-1 Medline

113. E. M. Weeks, J. C. Ulirsch, N. Y. Cheng, B. L. Trippe, R. S. Fine, J. Miao, T. A. Patwardhan, M. Kanai, J. Nasser, C. P. Fulco, K. C. Tashman, F. Aguet, T. Li, J. Ordovas-Montanes, C. S. Smillie, M. Biton, A. K. Shalek, A. N. Ananthakrishnan, R. J. Xavier, A. Regev, R. M. Gupta, K. Lage, K. G. Ardlie, J. N. Hirschhorn, E. S. Lander, J. M. Engreitz, H. K. Finucane, Leveraging polygenic enrichments of gene features to predict genes underlying complex traits and diseases. *Nat. Genet.* **55**, 1267–1276 (2023). doi:10.1038/s41588-023-01443-6 Medline

114. E. J. Gardner, K. A. Kentistou, S. Stankovic, S. Lockhart, E. Wheeler, F. R. Day, N. D. Kerrison, N. J. Wareham, C. Langenberg, S. O'Rahilly, K. K. Ong, J. R. B. Perry, Damaging missense variants in *IGF1R* implicate a role for IGF-1 resistance in the etiology of type 2 diabetes. *Cell Genom.* **2**, 100208 (2022). doi:10.1016/j.xgen.2022.100208 Medline

115. C. Bycroft, C. Freeman, D. Petkova, G. Band, L. T. Elliott, K. Sharp, A. Motyer, D. Vukcevic, O. Delaneau, J. O'Connell, A. Cortes, S. Welsh, A. Young, M. Effingham, G. McVean, S. Leslie, N. Allen, P. Donnelly, J. Marchini, The UK Biobank resource with deep phenotyping and genomic data. *Nature* **562**, 203–209 (2018). doi:10.1038/s41586-018-0579-z Medline

116. P. Rentzsch, D. Witten, G. M. Cooper, J. Shendure, M. Kircher, CADD: Predicting the deleteriousness of variants throughout the human genome. *Nucleic Acids Res.* **47**, D886–D894 (2019). doi:10.1093/nar/gky1016 Medline

117. G. Marenne, A. E. Hendricks, A. Perdikari, R. Bounds, F. Payne, J. M. Keogh, C. J. Lelliott, E. Henning, S. Pathan, S. Ashford, E. G. Bochukova, V. Mistry, A. Daly, C. Hayward, INTERVAL, UK10K Consortium, N. J. Wareham, S. O'Rahilly, C. Langenberg, E. Wheeler, E. Zeggini, I. S. Farooqi, I. Barroso, Exome Sequencing Identifies Genes and Gene Sets Contributing to Severe Childhood Obesity, Linking *PHIP* Variants to Repressed *POMC* Transcription. *Cell Metab.* **31**, 1107–1119.e12 (2020). doi:10.1016/j.cmet.2020.05.007 Medline

118. A. E. Hendricks, E. G. Bochukova, G. Marenne, J. M. Keogh, N. Atanassova, R. Bounds, E. Wheeler, V. Mistry, E. Henning, A. Körner, D. Muddyman, S. McCarthy, A. Hinney, J. Hebebrand, R. A. Scott, C. Langenberg, N. J. Wareham, P. Surendran, J. M. Howson, A. S. Butterworth, J. Danesh, B. G. Nordestgaard, S. F. Nielsen, S. Afzal, S. Papadia, S. Ashford, S. Garg, G. L. Millhauser, R. I. Palomino, A. Kwasniewska, I. Tachmazidou, S. O'Rahilly, E. Zeggini, I. Barroso, I. S. Farooqi, Understanding Society Scientific Group, EPIC-CVD Consortium, UK10K Consortium, Rare Variant Analysis of Human and Rodent Obesity Genes in Individuals with Severe Childhood Obesity. *Sci. Rep.* **7**, 4394 (2017). doi:10.1038/s41598-017-03054-8 Medline

119. UniProt Consortium, UniProt: The Universal Protein Knowledgebase in 2023. *Nucleic Acids Res.* **51**, D523–D531 (2023). doi:10.1093/nar/gkac1052 Medline

120. J. Jumper, R. Evans, A. Pritzel, T. Green, M. Figurnov, O. Ronneberger, K. Tunyasuvunakool, R. Bates, A. Žídek, A. Potapenko, A. Bridgland, C. Meyer, S. A. A. Kohl, A. J. Ballard, A. Cowie, B. Romera-Paredes, S. Nikolov, R. Jain, J. Adler, T. Back, S. Petersen, D. Reiman, E. Clancy, M. Zielinski, M. Steinegger, M. Pacholska, T. Berghammer, S. Bodenstein, D. Silver, O. Vinyals, A. W. Senior, K. Kavukcuoglu, P. Kohli, D. Hassabis, Highly accurate protein structure prediction with AlphaFold. *Nature* **596**, 583–589 (2021). doi:10.1038/s41586-021-03819-2 Medline

121. M. Lek, K. J. Karczewski, E. V. Minikel, K. E. Samocha, E. Banks, T. Fennell, A. H. O'Donnell-Luria, J. S. Ware, A. J. Hill, B. B. Cummings, T. Tukiainen, D. P. Birnbaum, J. A. Kosmicki, L. E. Duncan, K. Estrada, F. Zhao, J. Zou, E. Pierce-Hoffman, J. Berghout, D. N. Cooper, N. Deflaux, M. DePristo, R. Do, J. Flannick, M. Fromer, L. Gauthier, J. Goldstein, N. Gupta, D. Howrigan, A. Kiezun, M. I. Kurki, A. L. Moonshine, P. Natarajan, L. Orozco, G. M. Peloso, R. Poplin, M. A. Rivas, V. Ruano-Rubio, S. A. Rose, D. M. Ruderfer, K. Shakir, P. D. Stenson, C. Stevens, B. P. Thomas, G. Tiao, M. T. Tusie-Luna, B. Weisburd, H.-H. Won, D. Yu, D. M. Altshuler, D. Ardissino, M. Boehnke, J. Danesh, S. Donnelly, R. Elosua, J. C. Florez, S. B. Gabriel, G. Getz, S. J. Glatt, C. M. Hultman, S. Kathiresan, M. Laakso, S. McCarroll, M. I. McCarthy, D. McGovern, R. McPherson, B. M. Neale, A. Palotie, S. M. Purcell, D. Saleheen, J. M.

Scharf, P. Sklar, P. F. Sullivan, J. Tuomilehto, M. T. Tsuang, H. C. Watkins, J. G. Wilson, M. J. Daly, D. G. MacArthur, Exome Aggregation Consortium, Analysis of protein-coding genetic variation in 60,706 humans. *Nature* **536**, 285–291 (2016). doi:10.1038/nature19057 Medline

122. H. Wickham, M. Averick, J. Bryan, W. Chang, L. D. McGowan, R. François, G. Grolemund, A. Hayes, L. Henry, J. Hester, M. Kuhn, T. L. Pedersen, E. Miller, S. M. Bache, K. Müller, J. Ooms, D. Robinson, D. P. Seidel, V. Spinu, K. Takahashi, D. Vaughan, C. Wilke, K. Woo, H. Yutani, Welcome to the Tidyverse. *J. Open Source Softw.* **4**, 1686 (2019). doi:10.21105/joss.01686

123. J. R. Bedsaul, N. M. Carter, K. E. Deibel, S. M. Hutcherson, T. A. Jones, Z. Wang, C. Yang, Y.-K. Yang, J. L. Pomerantz, Mechanisms of Regulated and Dysregulated CARD11 Signaling in Adaptive Immunity and Disease. *Front. Immunol.* **9**, 2105 (2018). doi:10.3389/fimmu.2018.02105 Medline

124. C. Lu, J. Zhang, B. Wang, Q. Gao, K. Ma, S. Pei, J. Li, S. Cui, Casein kinase 1α is required to maintain murine hypothalamic pro-opiomelanocortin expression. *iScience* **26**, 106670 (2023). doi:10.1016/j.isci.2023.106670 Medline

125. P. Xu, P. Fischer-Posovszky, J. Bischof, P. Radermacher, M. Wabitsch, D. Henne-Bruns, A.-M. Wolf, A. Hillenbrand, U. Knippschild, Gene expression levels of Casein kinase 1 (CK1) isoforms are correlated to adiponectin levels in adipose tissue of morbid obese patients and site-specific phosphorylation mediated by CK1 influences multimerization of adiponectin. *Mol. Cell. Endocrinol.* **406**, 87–101 (2015). doi:10.1016/j.mce.2015.02.010 Medline

126. R. E. Mesías, Y. Zaki, C. A. Guevara, L. G. Friedman, A. Hussein, K. Therrien, A. R. Magee, N. Tzavaras, P. Del Valle, M. G. Baxter, G. W. Huntley, D. L. Benson, Development and cadherin-mediated control of prefrontal corticostriatal projections in mice. *iScience* **26**, 108002 (2023). doi:10.1016/j.isci.2023.108002 Medline

127. A. A. van der Klaauw, S. Croizier, E. Mendes de Oliveira, L. K. J. Stadler, S. Park, Y. Kong, M. C. Banton, P. Tandon, A. E. Hendricks, J. M. Keogh, S. E. Riley, S. Papadia, E. Henning, R. Bounds, E. G. Bochukova, V. Mistry, S. O'Rahilly, R. B. Simerly, INTERVAL, UK10K Consortium, J. E. N. Minchin, I. Barroso, E. Y. Jones, S. G. Bouret, I. S. Farooqi, Human Semaphorin 3 Variants Link Melanocortin Circuit Development and Energy Balance. *Cell* **176**, 729–742.e18 (2019). doi:10.1016/j.cell.2018.12.009 Medline

128. Catalogue of Somatic Mutations in Cancer (Cosmic), Cancer Gene Census; https://cancer.sanger.ac.uk/census.

129. B. Schittek, T. Sinnberg, Biological functions of casein kinase 1 isoforms and putative roles in tumorigenesis. *Mol. Cancer* **13**, 231 (2014). doi:10.1186/1476-4598-13-231 Medline

130. J. Shi, C. Cheng, J. Ma, C.-C. Liew, X. Geng, Gene expression signature for detection of gastric cancer in peripheral blood. *Oncol. Lett.* **15**, 9802–9810 (2018). doi:10.3892/ol.2018.8577 Medline

131. D. Chen, Y. Zhang, L. Meng, L. Lu, G. Meng, circRNA DENND1B inhibits tumorigenicity of clear cell renal cell carcinoma via miR-122-5p/TIMP2 axis. *Open Med.* **17**, 2085–2097 (2022). doi:10.1515/med-2022-0536 Medline

132. Z. Wang, M. Ding, N. Qian, B. Song, J. Yu, J. Tang, J. Wang, Decreased expression of semaphorin 3D is associated with genesis and development in colorectal cancer. *World J. Surg. Oncol.* **15**, 67 (2017). doi:10.1186/s12957-017-1128-1 Medline

133. D. Liu, J. Li, F. Qi, H. You, Semaphorins and their receptors in pancreatic cancer: Mechanisms and therapeutic opportunities. *Front. Oncol.* **12**, 1106762 (2023). doi:10.3389/fonc.2022.1106762 Medline

134. H. G. Parker, D. L. Dreger, M. Rimbault, B. W. Davis, A. B. Mullen, G. Carpintero-Ramirez, E. A. Ostrander, Genomic Analyses Reveal the Influence of Geographic Origin, Migration, and Hybridization on Modern Dog Breed Development. *Cell Rep.* **19**, 697–708 (2017). doi:10.1016/j.celrep.2017.03.079 Medline

135. C. A. Pugh, B. M. C. Bronsvoort, I. G. Handel, K. M. Summers, D. N. Clements, Dogslife: A cohort study of Labrador Retrievers in the UK. *Prev. Vet. Med.* **122**, 426–435 (2015). doi:10.1016/j.prevetmed.2015.06.020 Medline
