# Supplementary material for: Canine genome-wide association study identifies DENND1B as an obesity gene in dogs and humans
Source: Science. Author manuscript; Available in PMC 2026 Feb 2. (PMC7618706; doi:10.1126/science.ads2145)
Supplement: Supplementary Materials [file EMS211953-supplement-Supplementary_Materials.zip › science.ads2145_tables_s1_to_s15.zip!/science.ads2145_table_legends.pdf]

Table S1 – S15

Table S1: Items included in the Dog Obesity Risk Assessment (DORA) Questionnaire which contributed to owner control and food motivation scores (24). Owners respond on a 4 or 5 point Likert scale according to their level of agreement with each statement and responses are translated into a percentage. Overall food motivation score or owner control score are calculated as the mean response to the items within that category. For some items, the scores are reversed (as indicated) to ensure item scores are in the congruent direction for the overall factor score.

Table S2: Summary statistics for phenotypes in the Labrador populations reported in the study. In the discovery GWAS all dogs were kept as companions. In the wider population (used for examining the utility of PRS) there were also assistance dogs in the population. For each group, the summary statistics are provided for all dogs, and subgroups by role and color. 'Red' is not a recognized color for this breed in the Kennel Club registration system (considered officially as yellow) but was reported by owners and so is listed separately where it occurred. PRS, polygenic risk score; SD, standard deviation; BCS, body condition score.

Table S3: Regions of interest from BCS GWAS. Regions of interest tagged by lead SNP variant (in format chromosome:position) from GWAS for BCS via two methods: r2 measure of LD regions ≥ 0.8 (80%) or haplotype mapping using PLINK. The 'refined' region was either the mapped haplotype plus in-phase SNPs or, if the SNP did not map to a haploblock, the r2 ≥ 0.8 region.

Table S4: Candidate causative variants identified through analysis of high coverage whole genome sequencing data from 25 Labrador retrievers. Labrador WGS (>30x) BAM files were visualized in IGV to detect structural variants using the 'soft clip' function which identified multiple regions suggestive of the presence of a moderate to large insertion or deletion mutation which are denoted below as 'poorly defined indels'. GWAS region of interest is categorized by top SNP from each locus. Distance from tagging SNP is determined by position of SNP or first bp of insertion/deletion. Ref/Alt indicates reference or alternative base on the Canfam3.1 genome. Control/risk label is determined based on whether the variant is inherited with the risk or non-risk allele for the lead GWAS SNP for BCS in the Labrador GWAS (for >70% of alleles).

Table S5: Results of investigation of positional candidate genes for evidence of effects related to obesity. PheWAS data were interrogated in GWAS Atlas (https://atlas.ctglab.nl/PheWAS). To investigate the effect of gene knockout or other genetic manipulation in mouse, we used web portals of the International Mouse Phenotyping Consortium (https://www.mousephenotype.org/), the Mouse Phenome Database (https://phenome.jax.org/) and performed searches using PubMed. The Online Mendelian Inheritance in Man (OMIM) database was searched to determine whether mutations in each gene were known to cause Mendelian disease in humans. The GTEx portal (https://www.gtexportal.org/) and Human Protein Atlas (https://www.proteinatlas.org/) were interrogated to determine tissues in which gene expression was enriched. - indicates no phenotype of interest. N/A indicates data not available.

Table S6: Summary of human BMI associations for genes identified on the canine GWAS.

Table S7: Predicted causative gene for obesity-related traits in human loci syntenic to canine obesity GWAS loci. We interrogated human GWAS data on body mass index (BMI) and waist-hip ratio (WHR) adjusted for BMI in up to 806,834 and 694,649 individuals respectively from the GIANT study and circulating high density lipoprotein (HDL) and triglyceride levels in up to 1,253,277 European individuals from the Global Lipids Genetics Consortium (GLGC) study (up to n = 1,253,277). G2G uses a variety of information to predict causative gene at each locus. G2G – GWAS to Genes; GWAS – genome wide association study; BMI – body mass index; HDL – high density lipoprotein; WHR – waist-hip ratio; PoPS – polygenic priority score; pQTL – protein quantitative trait loci; eQTL – expression quantitative trait loci; ABC – activity by contact; MAGMA – Multi-marker Analysis of GenoMic Annotation; LD – linkage disequilibrium.

Table S8: Canine GWAS obesity candidate genes are expressed in a range of canine tissues. Data obtained from the BarkBase database *(41)* and expressed in fragments per kilobase of transcript per million read pairs (FPKM). NA not in database.

Table S9: Candidate gene expression in single cell RNA sequencing from mouse and human hypothalamus. We interrogated HypoMap, a unified single cell gene expression atlas of the mouse hypothalamus (42) and snRNAseq of the human hypothalamus in data from the human HYPOMAP (43). Results shown for each gene of interest show expression in all cell types, all neurons, Pomc-expressing cells and Mc4r-expressing cells. GHSR is included since it was used, like MC4R, in molecular studies of DENND1B function. Presented as a percentage of all cells in subgroup to 2 decimal places.

Table S10: Co-expression of DENND1B and other candidate genes with POMC, MC4R and GHSR in the human hypothalamus. In snRNAseq data from the human hypothalamus, cells were clustered according to gene expression patterns (42). The table shows cell type and gene markers defining each cluster along with the number of nuclei contributing to that data. Data shown: % <gene>+ (% of cells / cluster expressing gene); AverageExpression_<gene> (Average log-normalized expression of gene / cluster); % <gene1>+<gene2>+ (Percentage of cells / cluster co-expressing both genes).

Table S11: Rare variants predicted to be deleterious and found to be associated with obesity in either SCOOP or SOPP data sets. To test if regions and genes identified on the canine GWAS were also relevant to human obesity, we examined regions orthologous to those mapped in dogs in human data sets. Specifically, we tested for enrichment of rare (MAF <0.0026%), predicted deleterious (CADD ≥ 25) variants in the Severe Childhood Onset Obesity Project (SCOOP-UK) (35) (n = 982), compared to reference exomes of similar ancestry (gnomAD v2.1.1, n = 56,885); and investigated the Severe Obesity in Pakistani Population (SOPP) cohort of patients with severe, early onset obesity from a highly consanguineous population, all with unaffected parents and no prior genetic diagnosis (*44*, *45*), in which we focused on homozygous, predicted deleterious variants. The table shows variants identified using that approach and their frequency in different control populations including gNomad Non-Finnish European (NFE), NCBI Allele Frequency Aggregator (ALFA), Trans-Omics for Precision Medicine (TOPMed) participants and the 1000 Genomes database.

Table S12: Predictive value of PRS demonstrated by improved goodness of fit for obesity-related traits in Labrador retrievers when it is included in the model. The R^2 measure for goodness-of-fit within linear regression models is displayed for the combined Labrador retriever population and subgroups, for models predicting three obesity-related traits: BCS, body weight (kg) and food motivation. R^2 is displayed in its adjusted and unadjusted forms. All numbers rounded to 2sf. Food motivation on a scale of 1-100%. PRS – polygenic risk score; R^2 – linear regression goodness-of-fit measure; BCS – body condition score on a 9-point scale.

Table S13: PRS variant associations across purebred groups. Results of linear regression for each PRS SNP variant that contributed to the Labrador retriever PRS in other breed and population-specific minimal models. Data presented for each purebred cohort and phenotype combination as effect size β and significance p. Phenotypes are BCS, body weight (kg) and DORA food motivation.

Table S14: Clinical information and genotypes of probands and available family members from human patients with variants in CDH8 and CSNK1A1. Families delineated by background color. BMI SDS, body mass index standard deviation score (Z score).

Table S15: Results of tests for purifying selection. Two constraint metrics were used to evaluate the intolerance of a gene to high confidence predicted loss of function (pLoF) mutations in the gnomAD database. Results beyond the thresholds suggested by the developers of each metric (<0.4 for LOUEF and >0.9 for pLI) are in bold text. o/e, observed/expected.
